# Mechanism of Asymmetric Homologation of Alkenylboronic Acids with CF_3_-Diazomethane via Borotropic Rearrangement

**DOI:** 10.1021/acs.joc.3c02785

**Published:** 2024-03-25

**Authors:** Maria Biosca, Kálmán J. Szabó, Fahmi Himo

**Affiliations:** Department of Organic Chemistry, Arrhenius Laboratory, Stockholm University, SE-106 91 Stockholm, Sweden

## Abstract

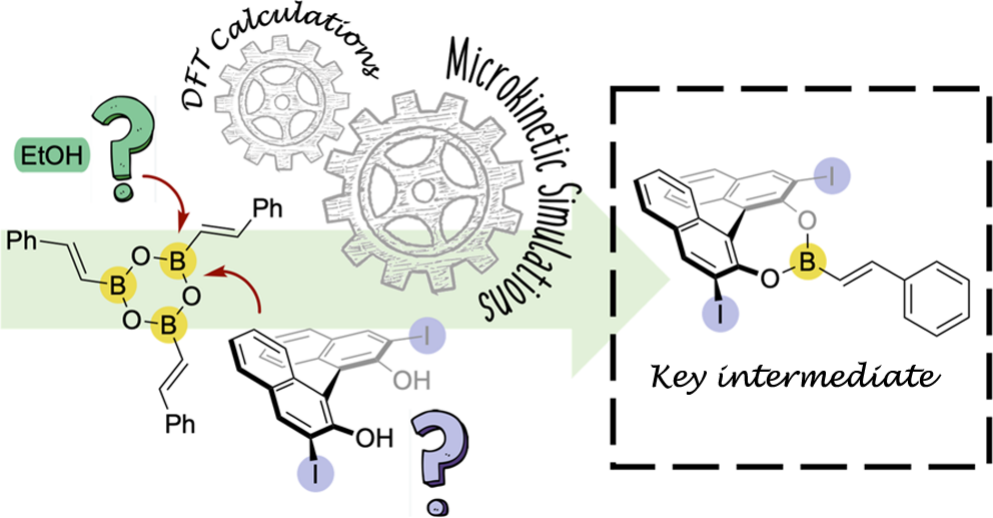

Density functional theory calculations have been performed to investigate the mechanism for the BINOL-catalyzed asymmetric homologation of alkenylboronic acids with CF_3_-diazomethane. The reaction proceeds via a chiral BINOL ester of the alkenylboronic acid substrate. The calculations reveal a complex scenario for the formation of the chiral BINOL-alkenylboronate species, which is the key intermediate in the catalytic process. The aliphatic alcohol additive plays an important role in the reaction. This study provides a rationalization of the stereoinduction step of the reaction, and the enantioselectivity is mainly attributed to the steric repulsion between the CF_3_ group of the diazomethane reagent and the γ-substituent of the BINOL catalyst. The complex potential energy surface obtained by the calculations is analyzed by means of microkinetic simulations.

## 1 Introduction

Asymmetric organocatalysis is an important tool for building molecular complexity in modern organic synthesis, a fact recognized by the 2021 Nobel Prize in chemistry of List and MacMillan.^1,2^ These reactions have distinctly different mechanisms compared with metal- and enzyme-catalyzed reactions. The exploration of their modes of action, including formation of catalytic intermediates, stereoinduction, and recovery of catalysts, is therefore indispensable for further development of this field.

Within asymmetric organocatalysis, a highly active and dynamically developing field is based on the homologation of organoboron compounds with carbenoid reagents. This methodological principle has attracted considerable attention in recent years as several valuable chiral organoboron reagents can be obtained through this approach.^3−20^

An interesting method for the homologation of organoboronates is based on the application of diazomethane derivatives. The first reactions were reported by the groups of Barluenga,^3^ Ley,^21,22^ Molander,^18^ and Wang^23^ on the homologation of alkenyl- and other boronic acids with diazomethane derivatives for the formation of a wide variety of allyl- and other boronic acids.^18,21−23^ Due to their high versatility for the synthesis of complex target molecules (e.g., via enantioselective allylboration or cross-coupling reactions), organoboronic acids are obviously some of the most used reagents in advanced organic synthesis.^24^ Noteworthily, the above-mentioned methodologies for the homologation of organoboronic acids with diazomethane derivatives provide only achiral products or racemates. The group of Arnold has reported the asymmetric homologation of organoboron compounds with CF_3_-diazo derivatives using enzyme catalysis to provide chiral alkyl- and benzylboron products.^25,26^

**Scheme 1:**
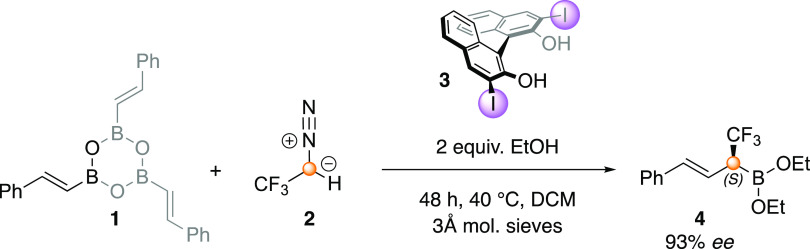
BINOL-Catalyzed Asymmetric Synthesis of α-CF_3_-Allylboronic Acid **4** by Homologation with the Diazo Derivative **2** Investigated in the Present Study

Recently, the group of Szabó reported a new asymmetric organocatalytic methodology for the synthesis of α-CF_3_ and α-SiMe_3_ organoboronic acids.^27−29^ One of these methods is based on the asymmetric homologation of alkenylboronic acids (e.g., cinnamyl boronic acid) with stabilized CF_3_-diazomethane derivatives using BINOL derivatives as organocatalysts and alcohol as an additive (Scheme 1).

The use of the CF_3_-diazo carbenoid reagent allows for the formation of α-CF_3_ allylboronic acids. De novo synthesis of chiral CF_3_ compounds leads to a considerable added value since the CF_3_ motif is of high interest for the pharmaceutical and agrochemical industries.^30−37^ With this approach, several α-CF_3_ and α-SiMe_3_ allylboronic acids could be obtained in good yields and high enantioselectivities using the (*R*)-I-BINOL catalyst (**3**) and ethanol (48–78% yield and 86–99% ee).^27^

**Scheme 2:**
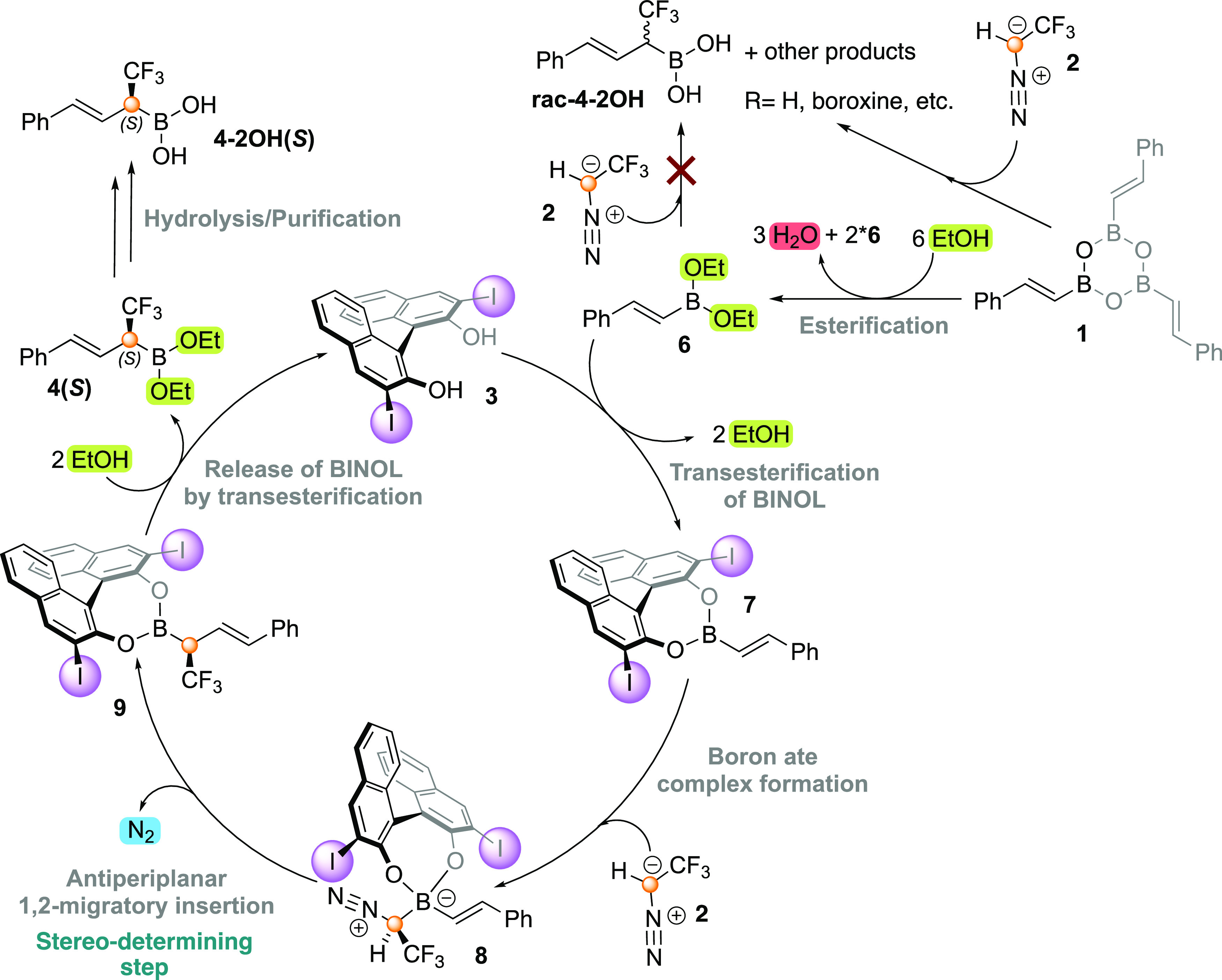
Previously Proposed Catalytic Cycle for the BINOL-Catalyzed Asymmetric Homologation of Alkenylboronic Acids with CF_3_-Diazomethane Derivatives Based on ref (27). Copyright 2020 American Chemical Society.

The mechanism shown in Scheme 2 has been proposed for the reaction.^27^ In analogy to other BINOL-catalyzed allylboration reactions developed by the group of Szabo,^38−40^ it was suggested that the ethanol additive helps in the prevention of undesired side reactions,^27^ namely, while alkenylboroxine **1** readily reacts with diazo compounds,^21,22,41^ the alkyl boronic ester **6**, formed by the addition of ethanol, was not expected to be reactive toward these derivatives. Due to the dynamic covalent bonding ability of boron,^42^ the alkyl boronic ester **6** is expected to undergo transesterification with (*R*)-I-BINOL (**3**) to form chiral alkenylboronate **7**. Next, **7** reacts with the diazomethane derivative (**2**) to form the boron ate complex **8**, which undergoes an antiperiplanar 1,2-migratory insertion to generate **9**. In the last step of the suggested catalytic cycle, the release of chiral BINOL by transesterification of **9** gives the desired product **4(*****S*****)**, which could be further hydrolyzed to obtain **4–2OH(*****S*****)**.

In the present work, we report a density functional theory (DFT) investigation to gain insight into the detailed mechanism of the chiral homologation reaction, as shown in Scheme 1. We start the study from boroxine and pay special attention to the key chiral alkenylboronate intermediate, considering different possible pathways for its formation.

Very recently, Zhang et al. reported a DFT study on the same reaction.^43^ However, in this study, the exploration of the requisite esterification/transesterification steps (such as **1** → **6**, **6** → **7**, and **9** → **4(*****S*****)**) was not comprehensive. Yet, the understanding of the mechanism of the complex esterification/transesterification steps is at least as important as the analysis of the stereoselection step of the homologation reaction. Although a stereoselection model was presented in the original experimental paper,^27^ the esterification/transesterification steps have not been fully explored previously, neither computationally nor experimentally. In previous computational studies involving BINOL catalysts in conjunction with organoboron compounds, the transition states for the ligand exchange processes were not explicitly located.^44^ In some cases, the thermodynamics of the transesterification was considered, but not the barriers of the process.^45−48^ The elucidation of the details of these esterification and transesterification processes will therefore provide more information about the intermediates formed in this transformation, giving very important new insights into this reaction mechanism, which is essential for the design of new related reactions.

There are important differences between our results and conclusions and those of Zhang et al.^43^ We will, for example, show below that the direct reaction between boroxine and BINOL is, unexpectedly, the most productive pathway for the formation of the chiral alkenylboronate intermediate. It should also be mentioned that the homologation part of the catalytic cycle was previously studied computationally for the nonasymmetric approach with TMS-diazo derivatives.^41,49^

## 2 Results and Discussion

We selected the asymmetric homologation of cinnamyl boronic acid with CF_3_-diazomethane (Scheme 1) as the model reaction for the present DFT study. We started the mechanistic investigation by examining possible pathways for the formation of the chiral BINOL-alkenylboronate key intermediate (**7** in Scheme 2). Since both ethanol and BINOL can perform the esterification of alkenylboroxine, two alternative pathways have to be considered for this step. As mentioned in the introduction, the esterification/transesterification processes for these kinds of reactions have not been explored in detail previously, and we therefore study all intermediates and transition states that are possible for both options. This is then followed by a presentation of the details of the homologation step and a discussion of the origins of the observed stereoselectivity. Next, we investigate the ethanolysis and hydrolysis reactions of the chiral BINOL-allylboronate intermediate (**9** in Scheme 2). Subsequently, we employ microkinetic simulations to gain further insight into the details of the obtained mechanism. Finally, we provide a comparison of the reactivities of different organoborane compounds that appear in the reaction mechanism toward the diazomethane derivative **2**.

### 2.1 Formation of the Chiral BINOL-Alkenylboronate Intermediate

**Scheme 3:**
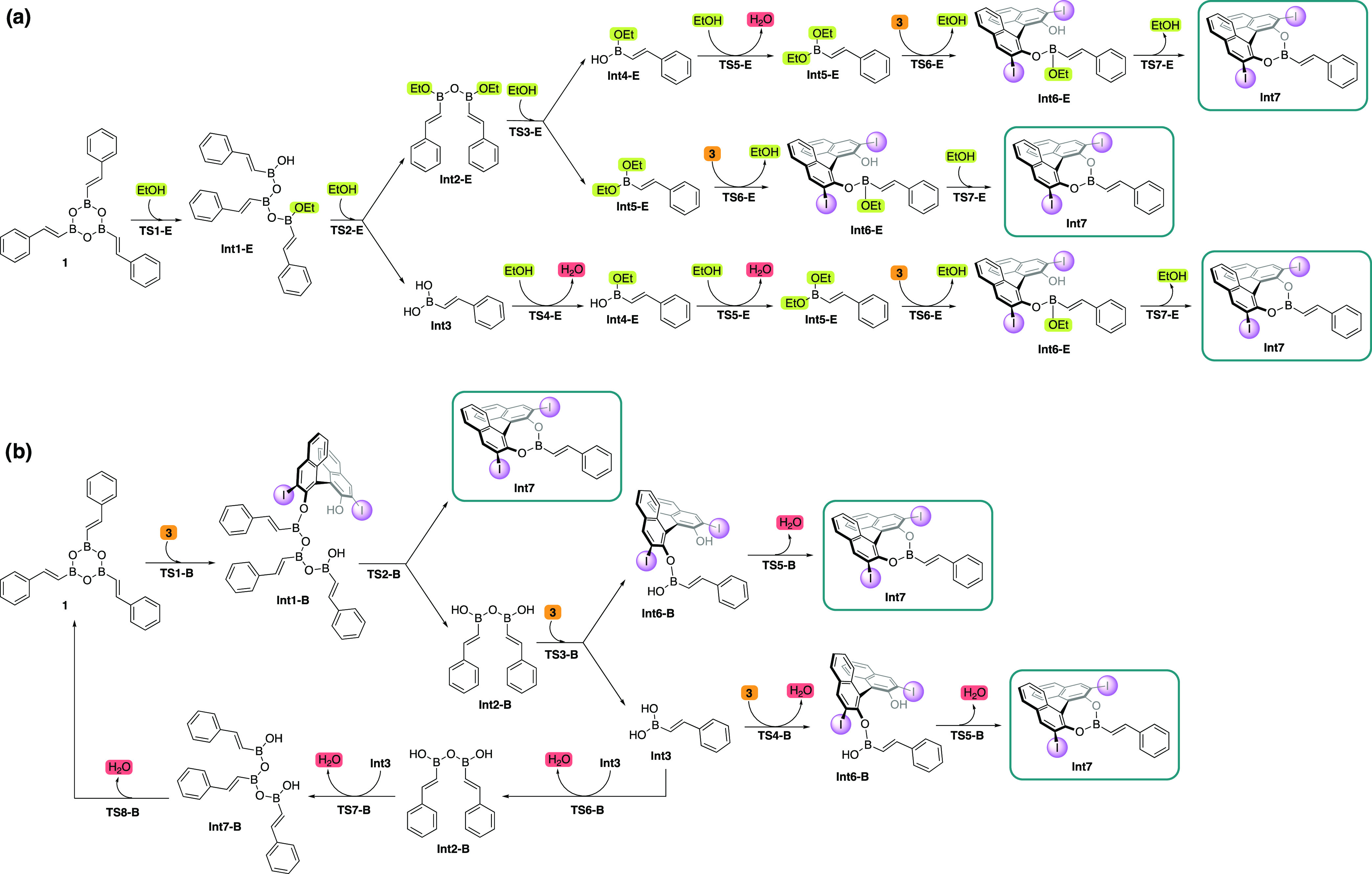
Summary of Different Possibilities for the Formation of Chiral BINOL-Alkenylboronate **Int7** (a) Opening of boroxine **1** by ethanol. (b) Opening of boroxine **1** by (*R*)-I-BINOL (**3**).

We start our investigation by studying the various pathways for the formation of the chiral BINOL-alkenylboronate intermediate from the starting alkenylboroxine (**1** → **Int7**, see Scheme 3). This reaction involves the alcoholysis of alkenylboroxine **1**. Boroxine **1**, the anhydride of cinnamyl boronic acid, may initially react either with ethanol (as previously proposed by Szabó and co-workers^27^) or with (*R*)-I-BINOL **3**, both of which are present in the reaction mixture. We considered both types of esterification reactions of **1**, and a summary of the possible pathways is given in Scheme 3. In the figures and schemes below, the transition states (**TS**s) and intermediates (**Int**s) that arise from the first alternative (Scheme 3a) are labeled with an “**E**” (esterification by ethanol) and the ones stemming from the second scenario (Scheme 3b) are labeled with a “**B**” (esterification by BINOL), while the intermediates common for both routes are not labeled with “**B**” or “**E**”. The calculated free energy profiles and optimized geometries of the intermediates and TSs are given in the Supporting Information. It is important to note here that since each boroxine molecule has three cinnamyl boronic acid units, its esterification/transesterification will lead to three molecules of **Int7**.

In the reaction of **1** with ethanol (Scheme 3a), the first step is the esterification of boroxine **1** to give **Int1-E**. The calculations show that the nucleophilic attack takes place via the six-membered transition state **TS1-E** with a calculated barrier of 20.6 kcal/mol. Interestingly, **TS1-E** involves an external proton-shuttling ethanol molecule. Nucleophilic attack without the assistance of a second ethanol molecule has a barrier of 34.7 kcal/mol (Supporting Information). This means that an external EtOH molecule is needed to catalyze the esterification of **1**. The involvement of a second alcohol molecule as a proton shuttle is consistent with the results of Zhang et al.^43^ In fact, all esterification/transesterification steps of this part of the reaction were found to be assisted by an additional ethanol molecule in order to obtain six-membered transition states.

Next, the further esterification of **Int1-E** can follow five different pathways because the three boron centers now have different substituents (see the Supporting Information). We optimized the TSs for all five possibilities, and the lowest-energy TS turned out to be **TS2-E**, in which esterification takes place at the central boron atom, resulting in **Int2-E** and an alkenylboronic acid (**Int3**), as shown in Scheme 3a. The barrier for this step is calculated to be 26.3 kcal/mol relative to boroxine **1** (again involving a second ethanol molecule as a proton shuttle).

Ester-anhydride **Int2-E** and cinnamyl boronic acid **Int3** can react with EtOH via different pathways to form the boronate diester **Int5-E**. **Int2-E** provides one molecule of **Int5-E** and one molecule of the boronate monoester **Int4-E** through **TS3-E**, while **Int3** undergoes two consecutive esterifications with EtOH via **TS4-E** and **TS5-E** to give **Int5-E**. These three transition states are associated with very similar energies (28.0, 27.9, and 29.3 kcal/mol for **TS3-E**, **TS4-E**, and **TS5-E**, respectively, each calculated relative to its own reactants; see the Supporting Information). From **Int5-E**, two transesterification steps with (*R*)-I-BINOL **3** take place, through transition states **TS6-E** and **TS7-E**, to yield chiral BINOL-alkenylboronate **Int7**. The overall barrier for this step is 24.2 kcal/mol (Supporting Information).

For the alternative reaction of boroxine **1** with (*R*)-I-BINOL **3** (Scheme 3b), the first step occurs via **TS1-B**, with a barrier of 16.1 kcal/mol (again assisted with an ethanol molecule). Next, **Int1-B** undergoes an attack by the second alcohol group of BINOL to form **Int7** and **Int2-B**. The barrier is calculated to be 18.9 kcal/mol relative to boroxine **1**. **Int2-B** can react with another molecule of BINOL with a barrier of 18.9 kcal/mol to form **Int3** and **Int6-B**. **Int6-B** can convert into **Int7** via **TS5-B** with a barrier of 17.3 kcal/mol, while from **Int3** (which also appears in the reaction with ethanol as discussed above), two pathways are possible, namely, the previously mentioned pathway that provides **Int5-E** (Scheme 3a) or the scenario where **Int3** is attacked by a third molecule of BINOL forming **Int6-B** that then is converted into **Int7**. From **Int3**, we also considered the possibility where this intermediate reacts with itself, leading to the reformation of boroxine **1** through three consecutive transition states (**TS6-B**, **TS7-B**, and **TS8-B**). The barriers for this pathway were, however, found to be higher than those for the formation of **Int7**. The energies for all of these pathways are given in the Supporting Information.

Since the different pathways branch at different precursors and intermediates and also share some common intermediates (such as **Int3**) and elementary steps, it is not straightforward to determine which of the two pathways shown in Scheme 3 is operative solely based on the calculated energies. We therefore use microkinetic simulations to resolve this issue. The results will be discussed in detail below (Section 2.4) after we present the entire reaction mechanism.

### 2.2 Homologation and Stereoinduction Steps

**Figure 1:**
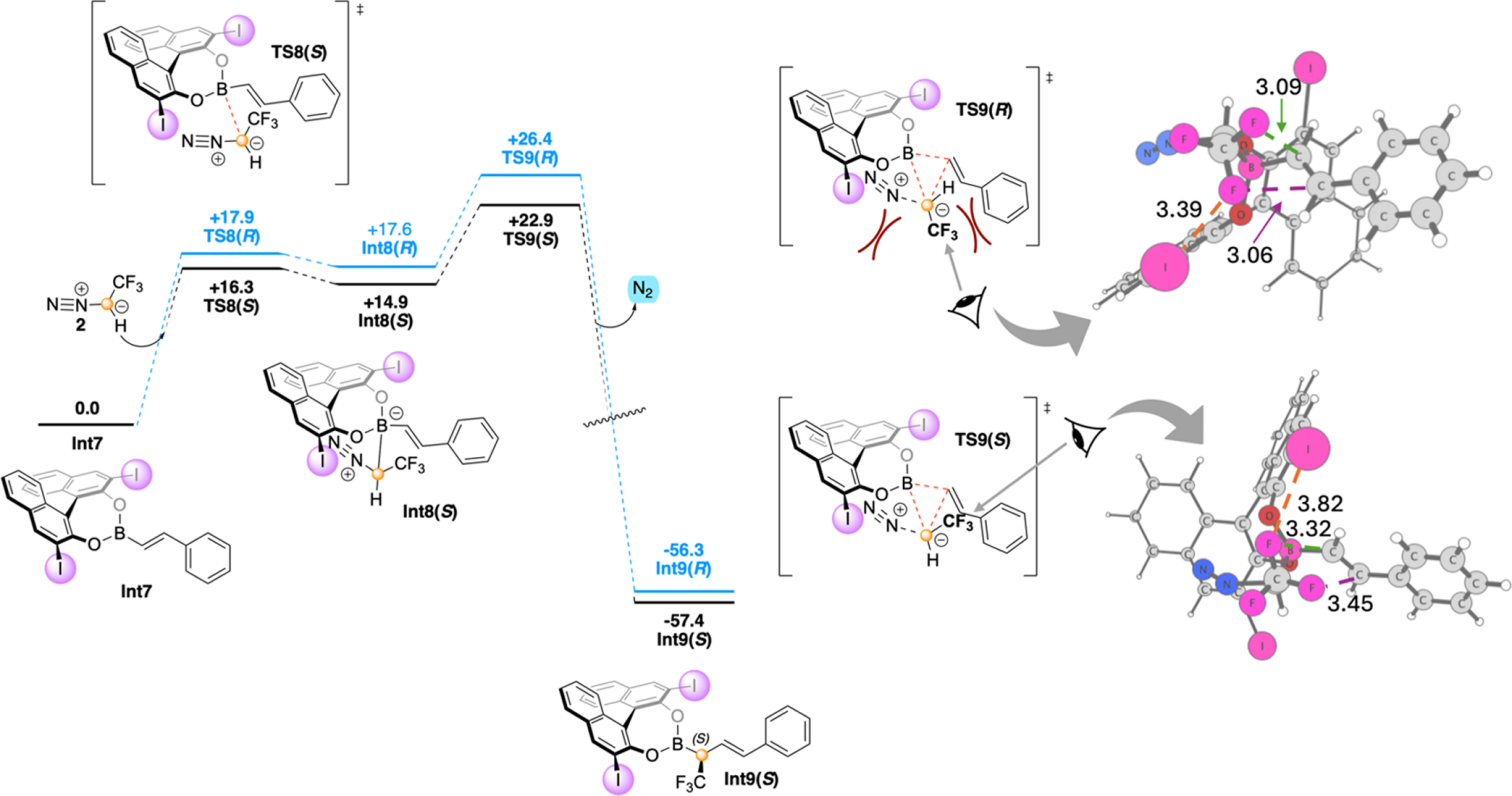
Calculated free energy profile (kcal/mol) for the stereoinduction in the homologation reaction starting from **Int7**, and optimized structures of 1,2-borotropic migration transition states **TS9(*S*)** and **TS9(*R*)** with relevant distances indicated in Ångström. Note that the energy of **Int7** is set to zero in this figure.

Once chiral BINOL-alkenylboronate **Int7** is formed (by either of the two mechanisms discussed above, Scheme 3), it reacts with the CF_3_-diazomethane derivative **2** via **TS8** to form the boron ate complex **Int8**, which is located in a shallow minimum on the energy surface (Figure 1). Subsequently, the 1,2-borotropic migration of the alkenyl (styryl) group to the carbenoid reagent takes place via **TS9**, releasing dinitrogen and forming chiral BINOL-allylboronate **Int9**. Since the attack of the diazo compound can take place with either the *Si*- or *Re*-face of the carbenoid, the resulting ate complex intermediate including a tetravalent boron can have either *S-* or *R*-configuration, respectively (**Int8(*****S*****)** or **Int8(*****R*****)**). The chiral carbon is maintained in the following steps, leading eventually to the *S*- or *R*-enantiomers of the allylboronate product **4**. The optimized geometries of the intermediates and transition states of this part of the reaction are given in the Supporting Information.

The stepwise mechanism obtained by the present calculations is consistent with the previous calculations on the achiral reaction,^41^ but different compared to the results of Zhang et al., where a concerted mechanism was found.^43^

The present calculations reproduce the experimental outcome, in that the pathway proceeding through the attack of the *Si*-face, thus leading to the *S*-product, is associated with lower energy barriers (Figure 1). The formation of the ate complexes (**Int7** → **Int8(*****R*****)** and **Int8(*****S*****)**) is a reversible process. Although the two pathways diverge at **TS8**, the 1,2-borotropic migration of **TS9** is higher in energy and is irreversible, making it the selectivity-determining step. The barrier for the *S*-pathway is 22.9 kcal/mol relative to that for **Int7**, while for the *R*-pathway, it is 26.4 kcal/mol. The calculated difference of 3.5 kcal/mol is consistent with the measured 93% ee in favor of **4(*****S*****)**.^27^

Our analysis of the optimized geometries of **TS9(*****S*****)** and **TS9(*****R*****)** shows that the main difference is that **TS9(*****R*****)** is destabilized by steric repulsions that are not present in **TS9(*****S*****)** (see Figure 1). In **TS9(*****R*****)**, the CF_3_ group clashes with the *ortho*-iodo-substituent of the BINOL catalyst. The F···I nonbonding distance is 3.39 Å in **TS9(*****R*****)**, while in **TS9(*****S*****)**, it is 3.82 Å. Another destabilizing repulsive interaction in **TS9(*****R*****)** is between the CF_3_ group and the substituent of the boronic substrate, with F···C distances of 3.06 and 3.09 Å. The corresponding distances in **TS9(*****S*****)** are 3.45 and 3.32 Å.

This analysis confirms that the substituent at the *ortho* position of BINOL is of key importance for the stereochemical outcome, as suggested by the experiments showing that bromo-substituted BINOL yields a lower *S*-selectivity and unsubstituted BINOL yields a low *R*-selectivity (both results obtained with a slightly different alkenylboroxine).^27^

It is interesting to point out that the conclusions obtained here regarding the origins of the selectivity are different from those suggested by Zhang et al., where weak attractive interactions were identified as the main difference between the *S*- and *R*-pathways.^43^ According to that study, one π···π, two C–H···I, and one C–H···F weak attractive interactions were observed in the TS leading to the *S*-product, while only one π···π, one C–H···F, and one C–H···I interactions were present in the TS leading to the *R*-product. Both the number and the strength of the C–H···I interactions were found to be different between the two TSs, and the C–H···I interaction was thus proposed to be important for the stereoselectivity.

### 2.3 Ethanolysis of the BINOL-Allylboronate Intermediate and Turning over the Catalyst

**Figure 2:**
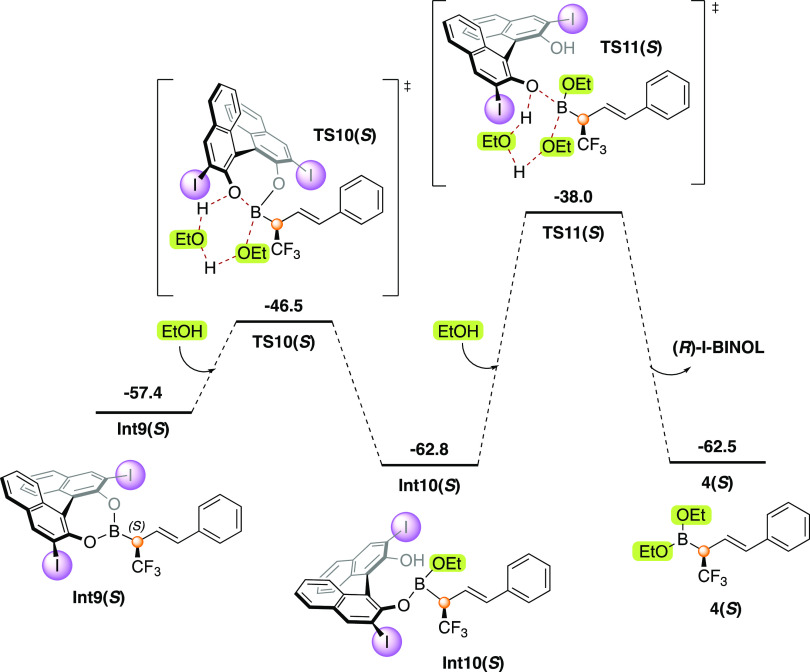
Calculated free energy profile (kcal/mol) for the ethanolysis of **Int9(*S*)** to give the final product **4(*S*)**. Note that the energies in this figure are given relative to **Int7** (see Figure 1).

The dynamic nature of the covalent bonding of boronic acid and the BINOL fragments is also crucial to the recovery of the catalyst. From chiral BINOL-derived allylboronate **Int9(*****S*****)**, the homologated product **4** and free BINOL **3** is accomplished by two transesterification steps via six-membered transition states **TS10(*****S*****)** and **TS11(*****S*****)**, with barriers of 10.9 and 24.8 kcal/mol, respectively (Figure 2). The optimized geometries of the intermediates and transition states of this part of the reaction are given in the Supporting Information.

The further hydrolysis of the allylboronate diester product **4(*****S*****)** to yield the corresponding allylboronate monoester **4-OH(*****S*****)** and further to allylboronic acid **4–2OH(*****S*****)** is hindered by the presence of molecular sieves in the reaction mixture. Nevertheless, we have computed these hydrolysis reactions, and the results are given in the Supporting Information.

### 2.4 Microkinetic Simulations

The DFT calculations described in the previous sections show a complex reaction scenario for the formation of the key chiral BINOL-alkenylboronate intermediate **Int7**. Two general pathways were identified, starting by an attack at the boroxine by either an ethanol molecule or a molecule of the catalyst (*R*)-I-BINOL **3** (Scheme 3), each alternative resulting in three equivalents of **Int7**. However, the mechanisms overlap partially, sharing a common intermediate in **Int3**, and therefore also common steps. Moreover, the overall barriers arising from the two pathways are calculated to be rather close in energy, making it very difficult to determine which of them is the operative one. Therefore, we used microkinetic simulations to gain better insight into the reaction mechanism.

A kinetic network was set up consisting of all elementary reactions. The rate constants for the forward and backward directions of each reaction were obtained from the calculated barriers using transition state theory, and the starting concentrations were set according to the experimental conditions (see the Supporting Information for the exact definition of the kinetic network).

Experimentally, product **4(*****S*****)** was obtained with 54% isolated yield after 2 days, which constitutes a lower limit for the conversion to **4(*****S*****)**. Using the energies obtained by the current calculations, the kinetic simulations show that 50% yield is achieved after ca. 230 days (see the Supporting Information), which means that the reaction time is overestimated compared to the experiments. Nevertheless, this can be considered quite satisfactory, bearing in mind that a small error in the barriers leads to a large error in the reaction time due to the exponential nature of the relationship between the two.

A noteworthy result from the kinetic simulations is that even at very long reaction times, the reaction was not able to reach 100% formation of product **4(*****S*****)**, and arrives at only a little more than 80% (see Supporting Information). This is because the last step, **Int10(*****S*****)** to **4(*****S*****)**, is calculated to be slightly endergonic. Making this step irreversible in the kinetic simulations results in the reaction reaching a higher conversion in a shorter time (Supporting Information).

Next, we conducted a sensitivity analysis in which the barriers of the elementary reactions were increased/decreased by 1.4 or 2.8 kcal/mol, corresponding to a decrease/increase in the individual rates by a factor of 10 or 100, respectively (see the Supporting Information). This analysis shows that **TS9**, i.e., the 1,2-borotropic migration step, and **TS11**, i.e., the final step of the reaction, which is the transesterification of **Int10**, are both rate-determining, i.e., an increase in the energy of any of these two TSs leads to a slower overall reaction, while a decrease in the energy of one of them does not lead to a significantly faster overall reaction because the other barrier becomes rate-limiting.

Very interestingly, the simulations show that the boroxine molecules are almost entirely opened by reaction with the BINOL catalyst (see the Supporting Information for details). More precisely, at 50% conversion, 99.98% of **Int7** stems from the reaction of **1** with BINOL (Scheme 3b), and only less than 0.02% originates from the reaction with ethanol via intermediates, such as **Int4-E** or **Int5-E** (Scheme 3a). These are unexpected results, as the boroxine has previously been assumed to be opened by an ethanol molecule, in particular considering that ethanol is used in large excess.^27^

### 2.5 Reactivity of Various Boronic Acid Esters/Anhydrides

As shown above (Scheme 3), due to the dynamic covalent bonding of boron to alcohols, a large number of boronic esters form in the reaction mixture. Except for **Int7**, most of these species would give racemic homologation products or low enantioselectivity. In order to gain further insight into why chiral BINOL-alkenylboronate **Int7** reacts favorably with the diazomethane compound **2** compared to other organoboron compounds, we have calculated the barriers for the 1,2-migratory insertion step (corresponding to **TS9**) starting from various other organoboron intermediates that appear in the reaction, namely, **1**, **Int1-B**, **Int2-B**, **Int3**, **Int6-B**, and **Int5-E**. The obtained barriers are given in Table 1. Among the different intermediates, the reactivities of **Int1-B** and **Int6-B** are of special interest since they could also provide a chiral final product. The reactivity of **Int3** is also important since the transesterification of this compound has the highest barrier of the catalytic cycle. The reaction of **Int5-E** is included in order to examine the initial assumption that this intermediate is unreactive toward **2** (see Scheme 2).

**Table 1:**
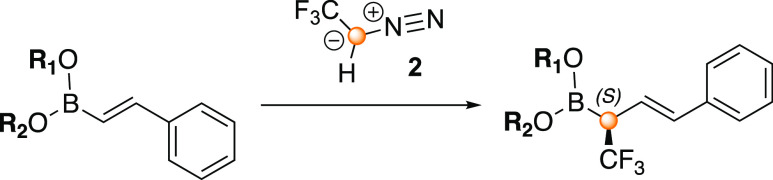

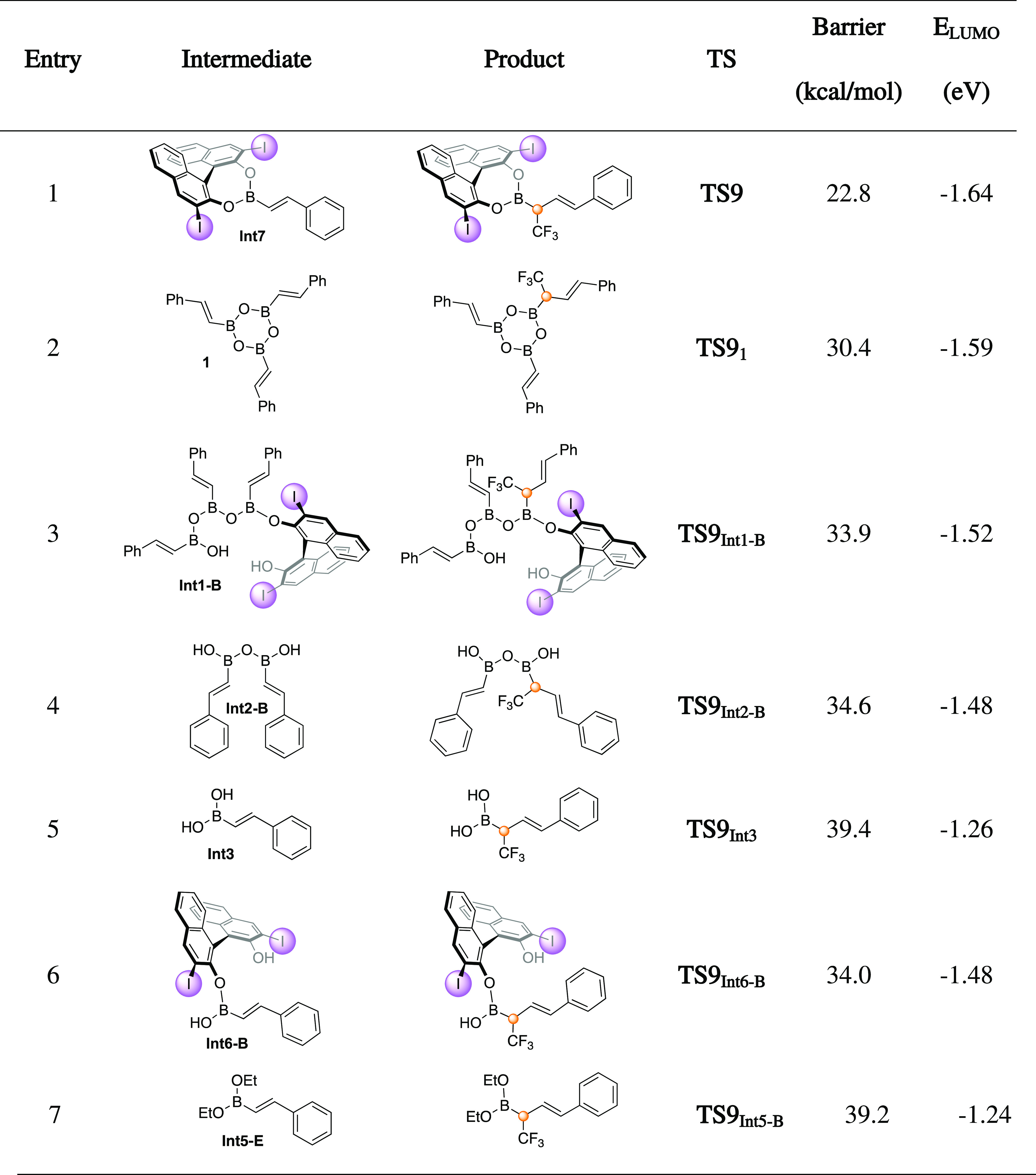
Computed Barriers for the 1,2-Migratory Insertion Step (**TS9**) of the Key Organoboron Compounds (**Int7**, **1**, **Int1-B**, **Int2-B**, **Int3**, **Int6-B**, and **Int5-E**)^a^

a Each barrier is given relative to its intermediate.

The calculations show that BINOL-derived alkenylboronate **Int7** is by far the most reactive species with a barrier of 22.8 kcal/mol. The reaction with boroxine **1** has a barrier of 30.4 kcal/mol, while the reactions with the other organoboron intermediates are associated with even higher barriers (Table 1). These results demonstrate that the less stable intermediate **Int7** reacts faster than more stable intermediates.

Zhang et al. conducted a similar analysis, comparing **Int7** with **1** and **Int5-E**. It was concluded that the lowest unoccupied molecular orbital (LUMO) orbital of the former is lower than the other two, causing the reaction to be faster.^43^ In this study, we also find a very good correlation between the LUMO energy and the reactivity of the various species (Table 1).

The results show that the esterifying groups (BINOL, O-bridge, OH, OEt, etc.) influence the LUMO energy significantly, thus affecting the accessibility of the LUMO toward nucleophiles, such as **2**. The reason for the low LUMO energy of **Int7** is the partial delocalization of the lone-pair of oxygen to the aromatic system of BINOL. As a consequence, the conjugation of the lone-pair of oxygen to the empty p orbital of boron is less efficient, which increases the electrophilicity of boron.

The fact that the highest reactivity is calculated for **Int7** is essential to the high enantioselectivity of the reaction. Achiral boronates, such as **1**, **Int1**, **Int2-B**, and **Int5-E**, would give racemic products, and the steric repulsion in other forms of boronate, such as **Int1-B** and **Int6-B**, is less than that in **Int7** and would likely not lead to high enantioselectivity. This type of activation effect of BINOL in **Int7** is analogous to chiral ligand acceleration, which is a basic principle in asymmetric catalysis with transition metal complexes.^50^

## 3 Conclusions

**Scheme 4:**
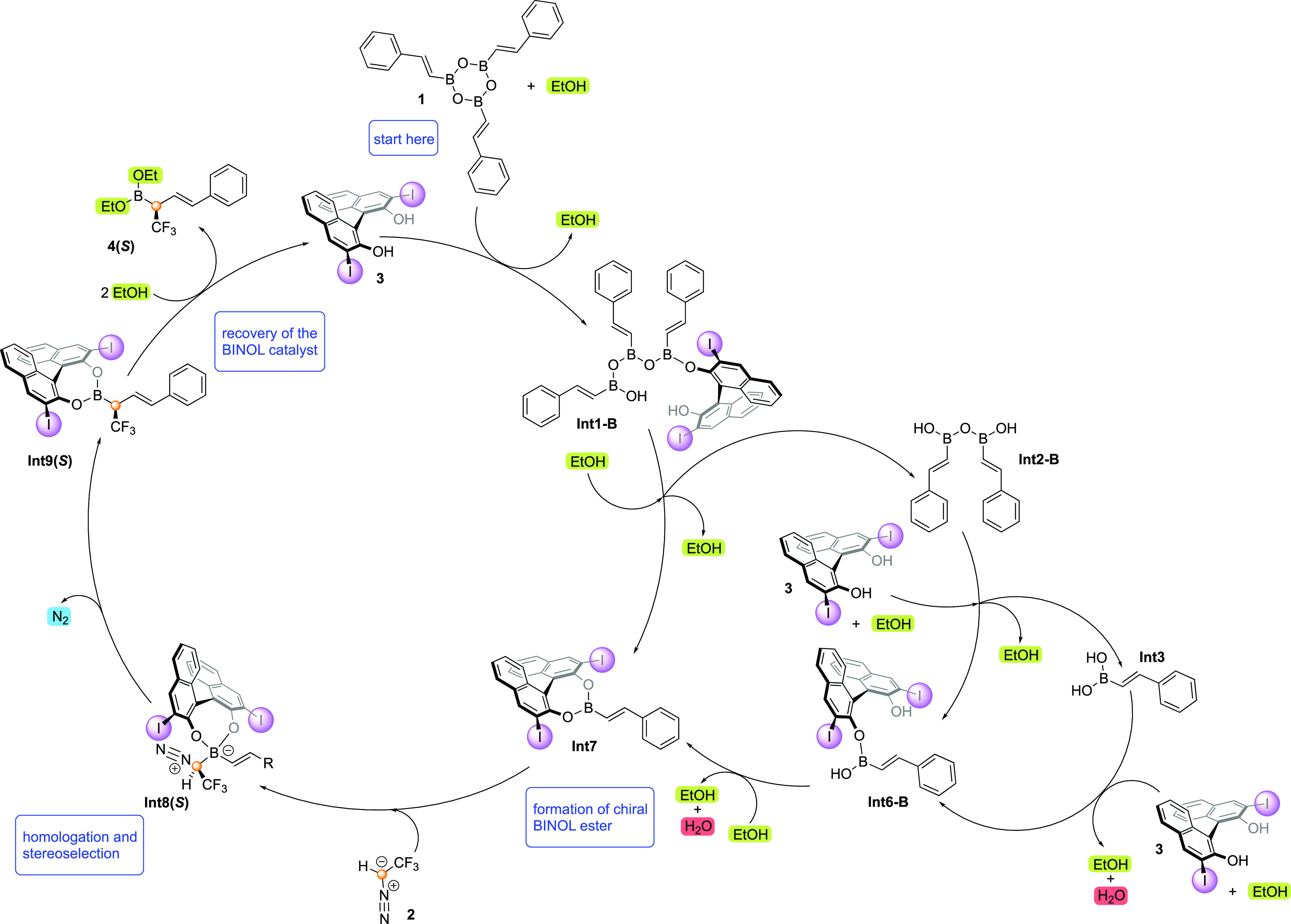
Summary of the Catalytic Cycle Obtained from the Calculations and the Microkinetic Simulations

In the present work, we report a mechanistic study on the BINOL-catalyzed asymmetric homologation of alkenylboronic acids with the CF_3_-diazomethane derivative by means of a combination of DFT calculations and microkinetic simulations. The mechanism that emerges from this study is summarized in Scheme 4.

A thorough analysis of the dynamic covalent bonding of boron with a number of available reaction partners is first conducted showing that the boroxine molecule is opened by reaction with the BINOL catalyst and not by reaction with ethanol as previously proposed.

Consistent with the previous computational work by Zhang et al.,^43^ the ethanol additive is shown to play an important role in promoting the proton transfer events of the reaction, thus lowering the barriers for the formation of **Int7** and for the ethanolysis steps of the catalytic cycle to generate the final product. This role of ethanol is also indirectly important for the enantioselectivity of the reaction, as the lower barriers that result from adding ethanol suppress the alternative reactivity of boroxine with the carbenoid reagent, which gives a racemic product.

The calculations reproduce well the stereochemical outcome of the reaction, and the factors determining the enantioselectivity are mainly attributed to steric repulsions of the CF_3_ group of diazomethane with both the *ortho*-substituents of the BINOL catalyst and the substituent of the substrate.

We also considered the reactivity of various organoboron intermediates toward the 1,2-migratory insertion step and found a good correlation with their LUMO energies. The high enantioselectivity of the homologation reaction relies on the formation of chiral alkenylboronate **Int7** (Scheme 3), which has a much higher reactivity toward the nucleophile **2** than the achiral boronate species (Table 1).

The insights obtained by the present work will be of value when setting up new reactions for the asymmetric homologation of practically all types of organoboronates and also other species.

## 4 Computational Details

The calculations were carried out using the B3LYP-D3(BJ) functional^51−54^ as implemented in the Gaussian 16 program package.^55^ For the geometry optimizations, the LANL2DZ pseudopotential^56^ was used for iodine, and the 6-31G(d,p) basis set was used for all other atoms. Implicit solvation using the SMD model^57^ with the parameters for dichloromethane was included in the geometry optimization. To obtain better accuracy, single-point calculations were carried out on the basis of the optimized structures with the same basis set for iodine and the 6-311+G(2d,2p) basis set for all other elements and using implicit solvation as in the geometry optimization. Frequencies were calculated at the level of theory of the geometry optimization using the temperature of the experiments of 313.15 K. The reported energies are Gibbs free energies in solution. A thorough conformational search was carried out on all stationary points in order to make sure that the structure with the lowest energy was located. The kinetic simulations were carried out using COPASI software (version 4.34).^58^

## Data Availability

The data underlying this study are available in the published article and its Supporting Information.

## Supporting Information Available

Additional computational results discussed in the text; microkinetic simulation analysis; absolute energies and energy corrections; and Cartesian coordinates (PDF)
