# Supplementary material for: Mechanism of Asymmetric Homologation of Alkenylboronic Acids with CF3-Diazomethane via Borotropic Rearrangement
Source: J Org Chem. 2024 Mar 25;89(7):4538–48. doi: 10.1021/acs.joc.3c02785 (PMC11002940; doi:10.1021/acs.joc.3c02785)
Supplement: Supplementary file 1 — jo3c02785_si_001.pdf [file jo3c02785_si_001.pdf]

Mechanism of Asymmetric Homologation of Alkenylboronic Acids with CF_3-diazomethane via Borotropic Rearrangement

Maria Biosca, Kálmán J. Szabó, Fahmi Himo**

Department of Organic Chemistry, Arrhenius Laboratory, Stockholm University, SE-106 91 Stockholm, Sweden.

E-mail: kalman.j.szabo@su.se; fahmi.himo@su.se

Contents

1. Optimized structures of intermediates and transition states S2

2. Formation of **Int7** by the opening of boroxine **1** with EtOH S6

3. Esterification of boroxine **1** with ethanol: 6-membered vs 4-membered TSs........ S8

4. Alternative pathways for the esterification of **Int1-E** S9

5. Formation of **Int7** by the opening of boroxine **1** with (*R*)-I-BINOL **3** S10

6. Formation of boroxine **1** from **Int3** S12

7. Hydrolysis of **4(*S*)** to **4-2OH(*S*)** S13

8. Optimized transition states for 1,2-migratory insertion S14

9. Details of the kinetic network used in the simulations S15

10. Absolute energies and energy corrections S21

11. Cartesian coordinates S23

1. Optimized structures of intermediates and transition states

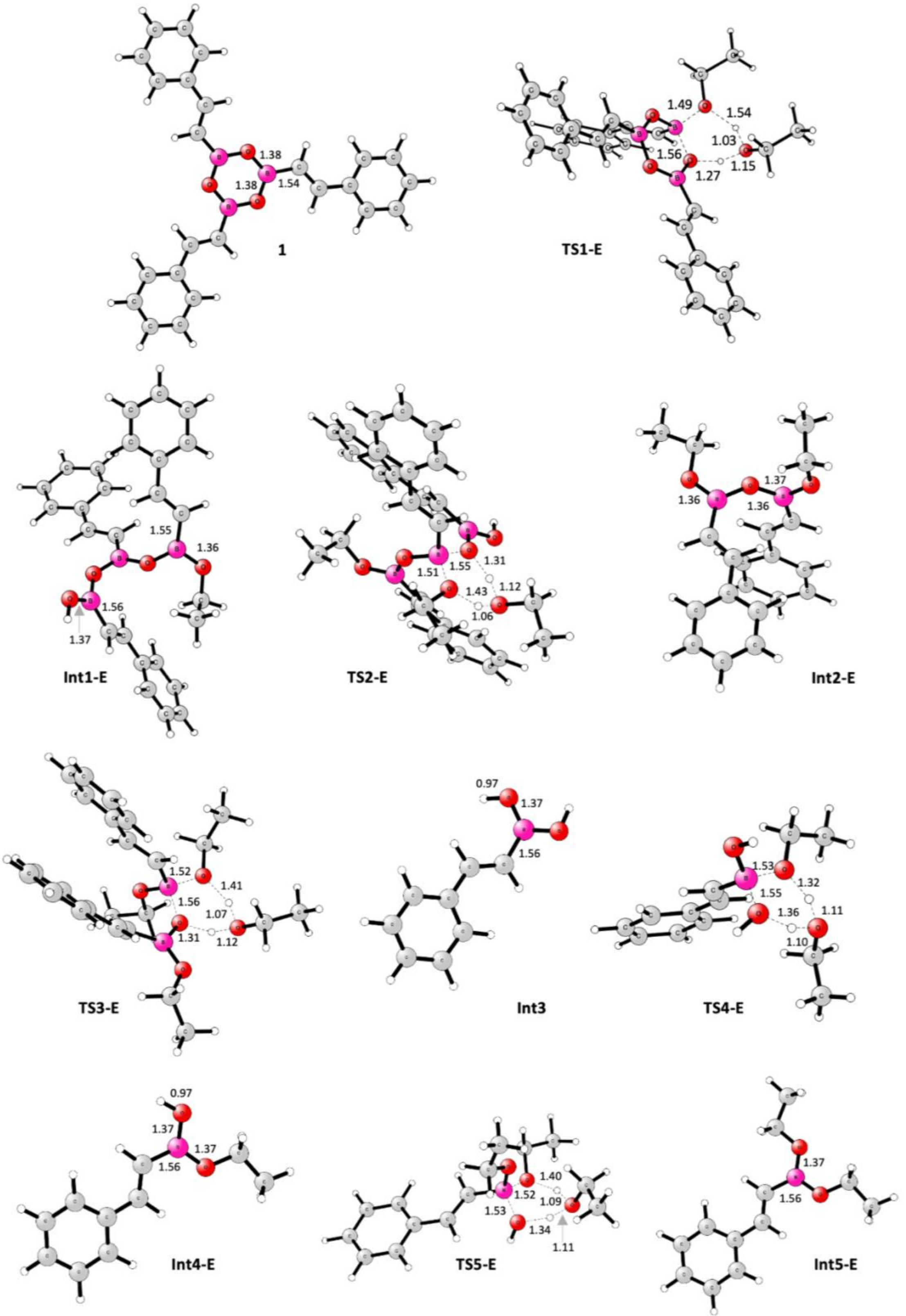

Figure S1-1. *Optimized structures of intermediates and transition states along the reaction pathway. Selected bond distances are indicated in Å.*

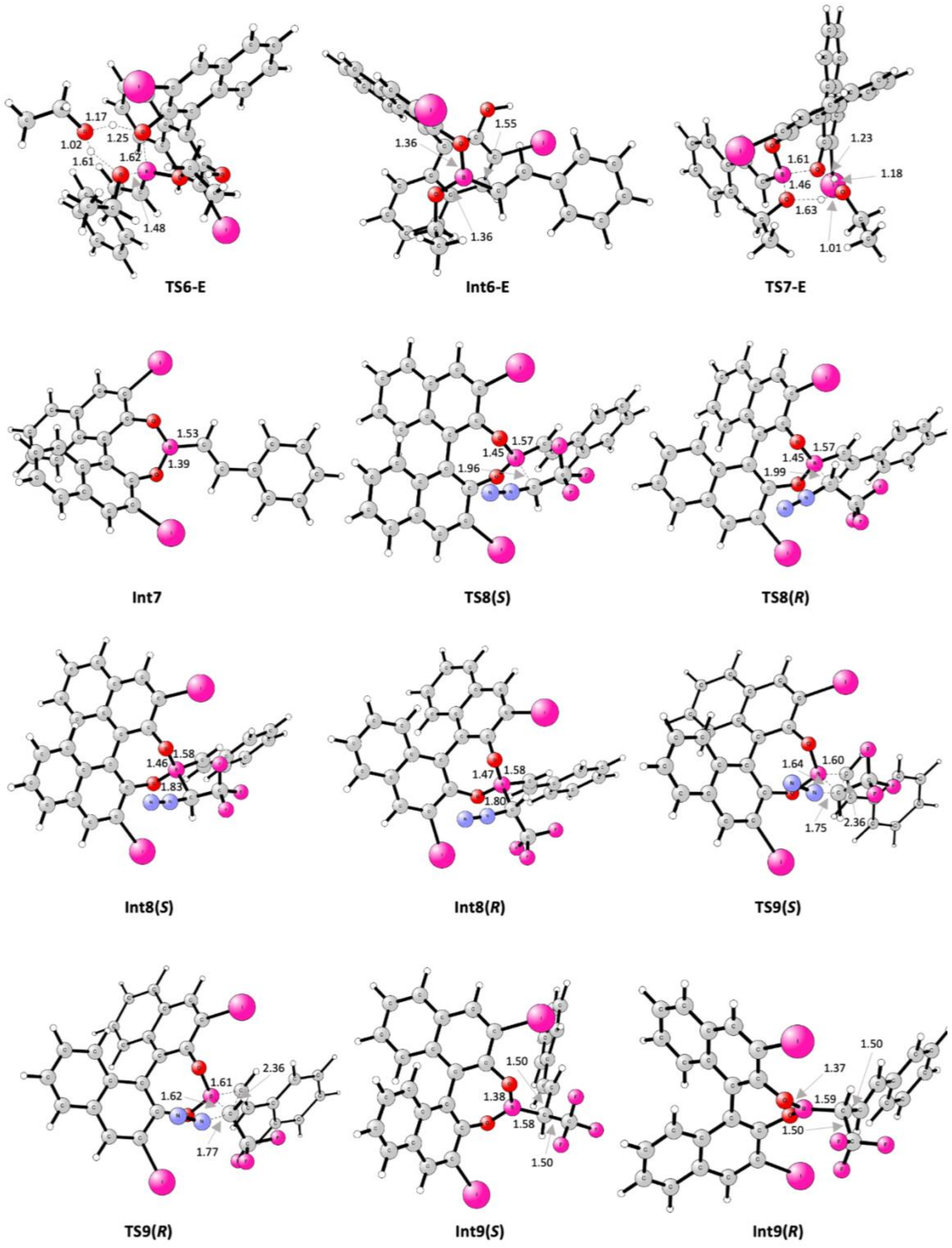

Figure S1-2. *Optimized structures of intermediates and transition states along the reaction pathway. Selected bond distances are indicated in Å.*

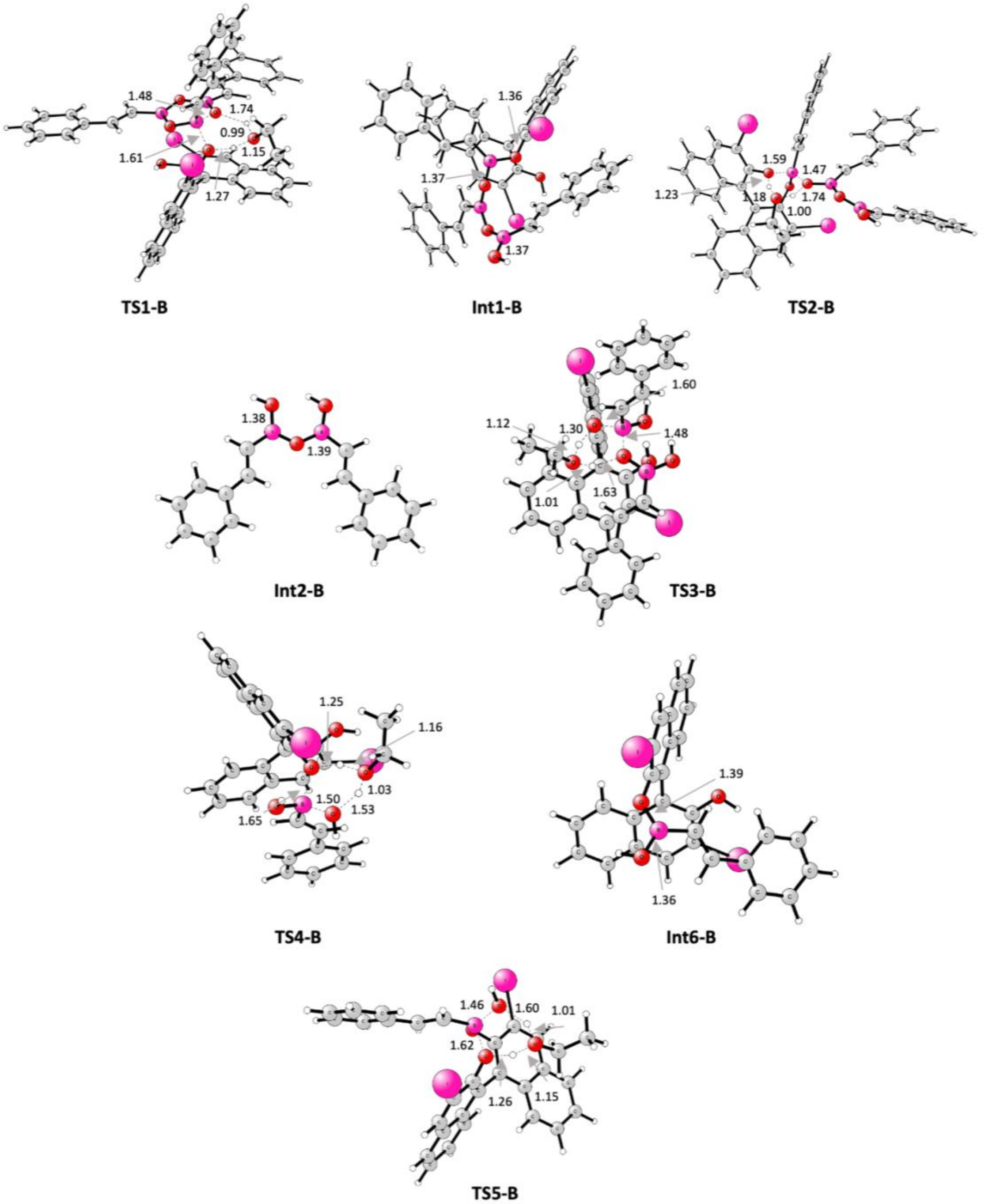

Figure S1-3. *Optimized structures of intermediates and transition states along the reaction pathway. Selected bond distances are indicated in Å.*

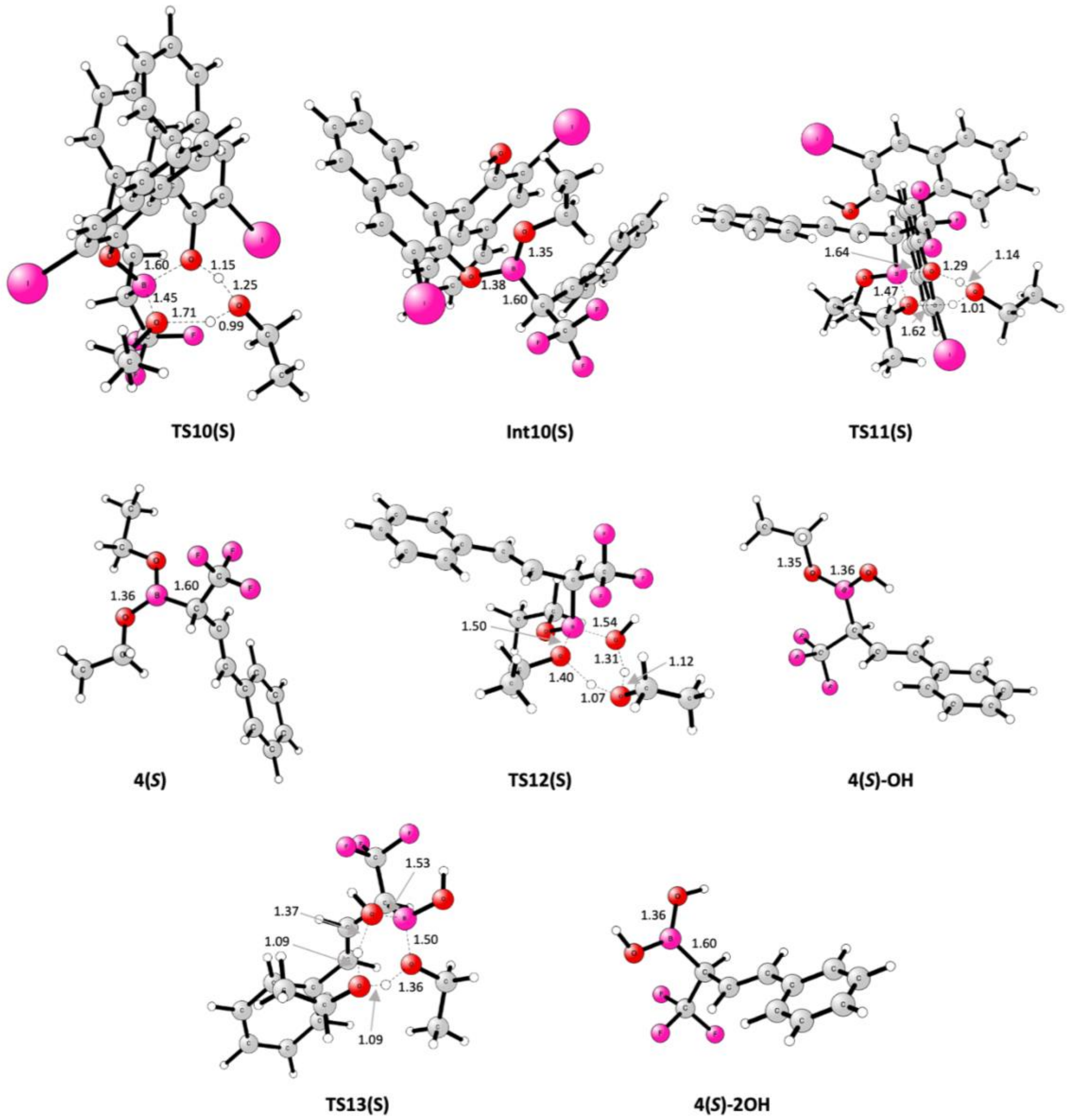

Figure S1-4. *Optimized structures of intermediates and transition states along the reaction pathway. Selected bond distances are indicated in Å.*

2. Formation of Int7 by the opening of boroxine 1 with EtOH

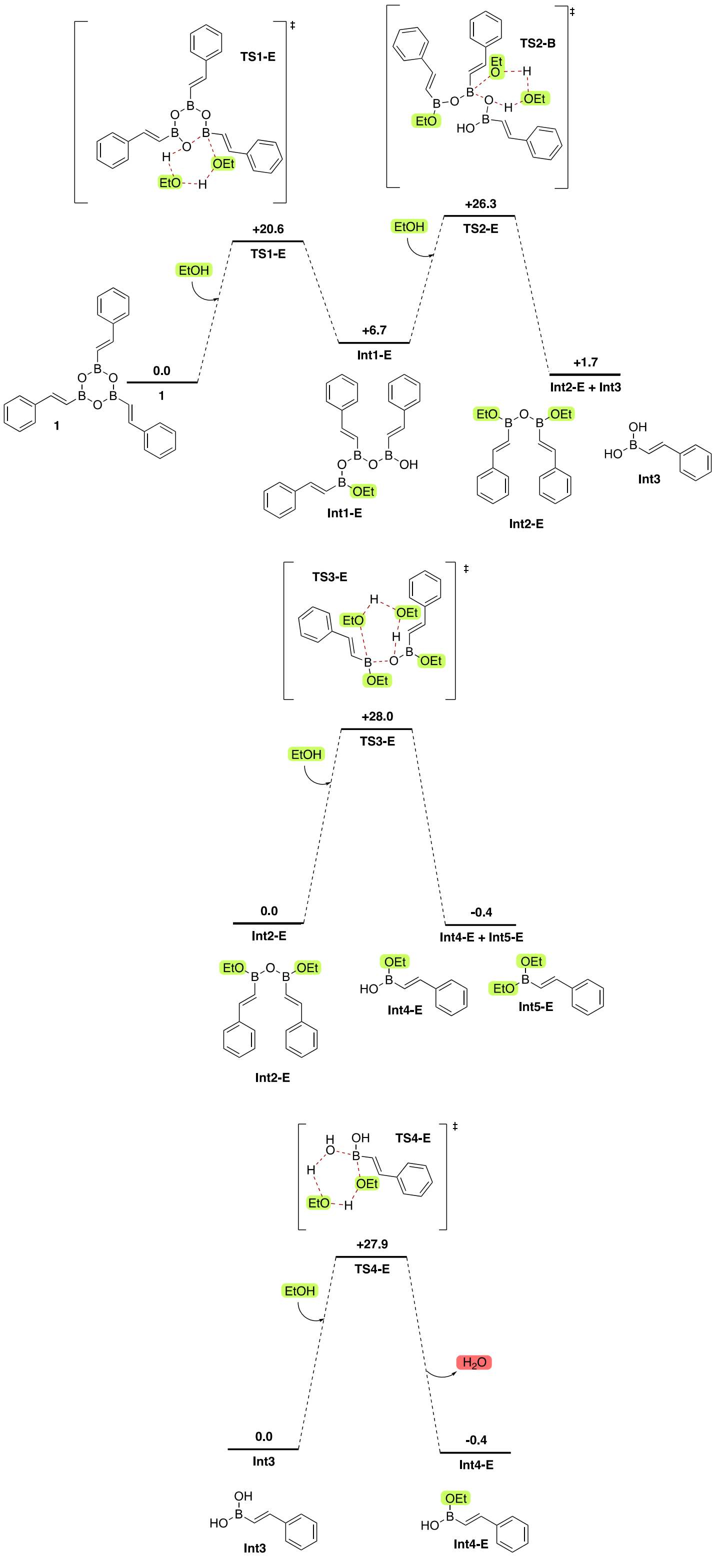

Figure S2-1. *Calculated free energy profiles (kcal/mol) for the formation of* ***Int7*** *by the opening of the boroxine* ***1*** *with ethanol.*

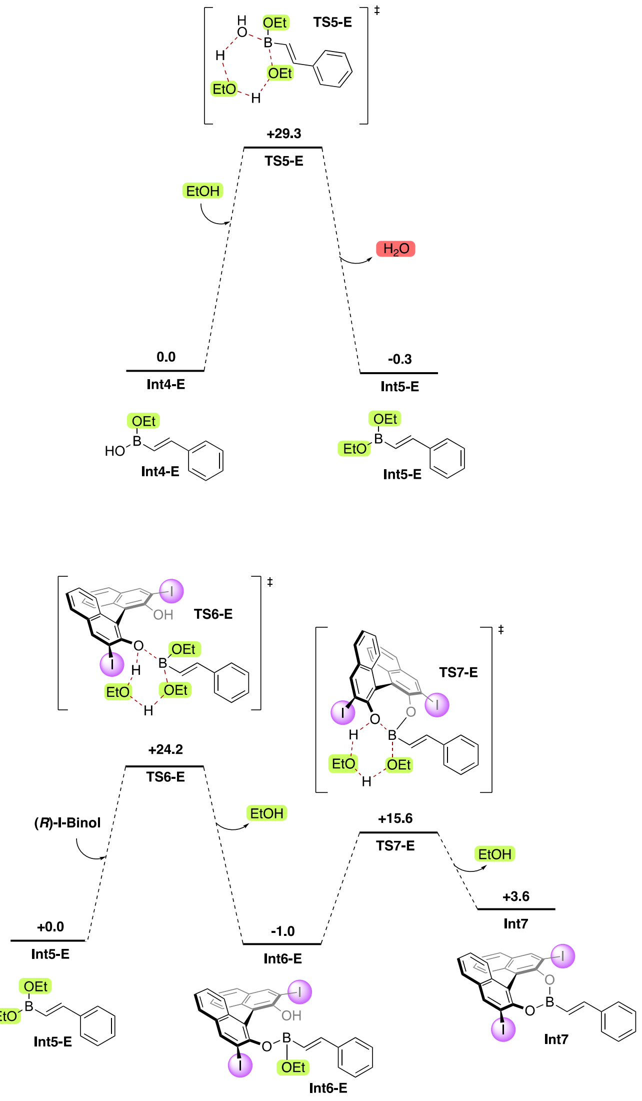

Figure S2-2. *Calculated free energy profiles (kcal/mol) for the formation of* ***Int7*** *by the opening of the boroxine* **1** *with ethanol.*

3. Esterification of boronxine 1 with ethanol: 6-membered vs 4-membered TSs

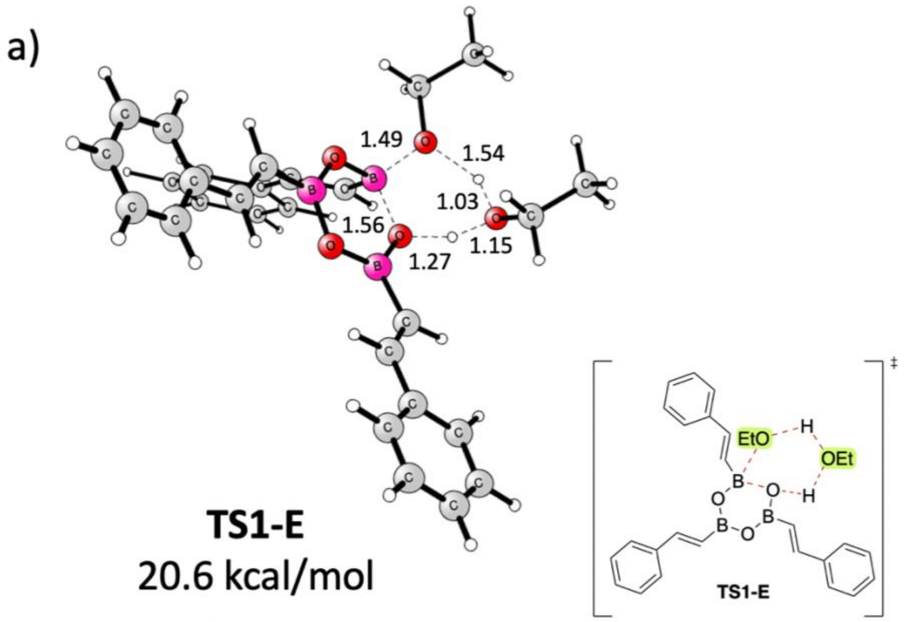

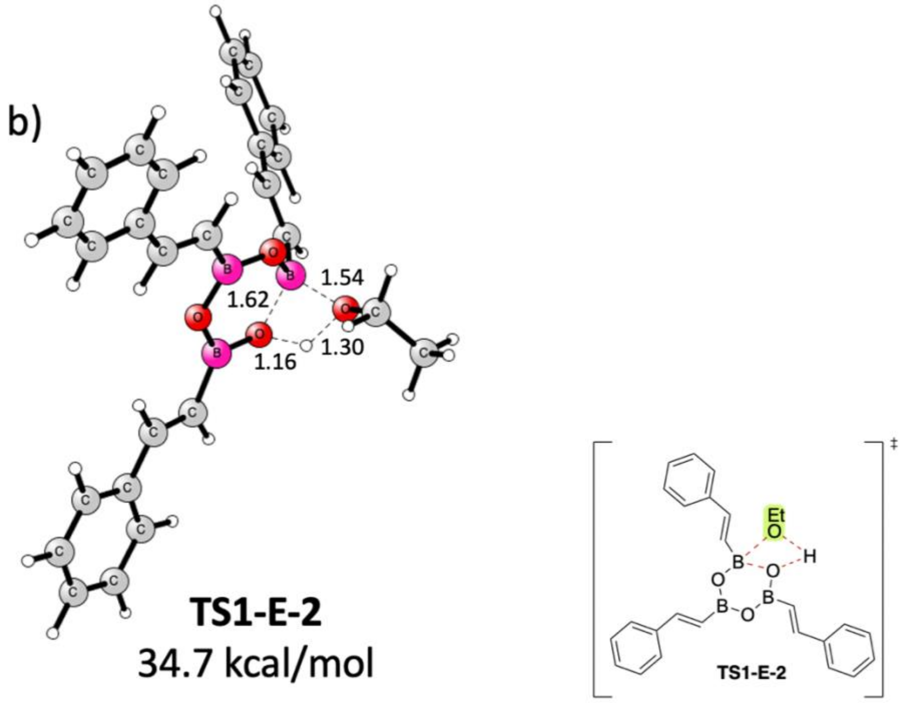

Figure S3. *Optimized structures of transition states for the esterification of boroxine* **1** *with ethanol. a) Two molecules of ethanol involved in the TS; b) One molecules of ethanol involved in the transition state. Selected bond distances are indicated in Å.*

4. Alternative pathways for the esterification of Int1-E

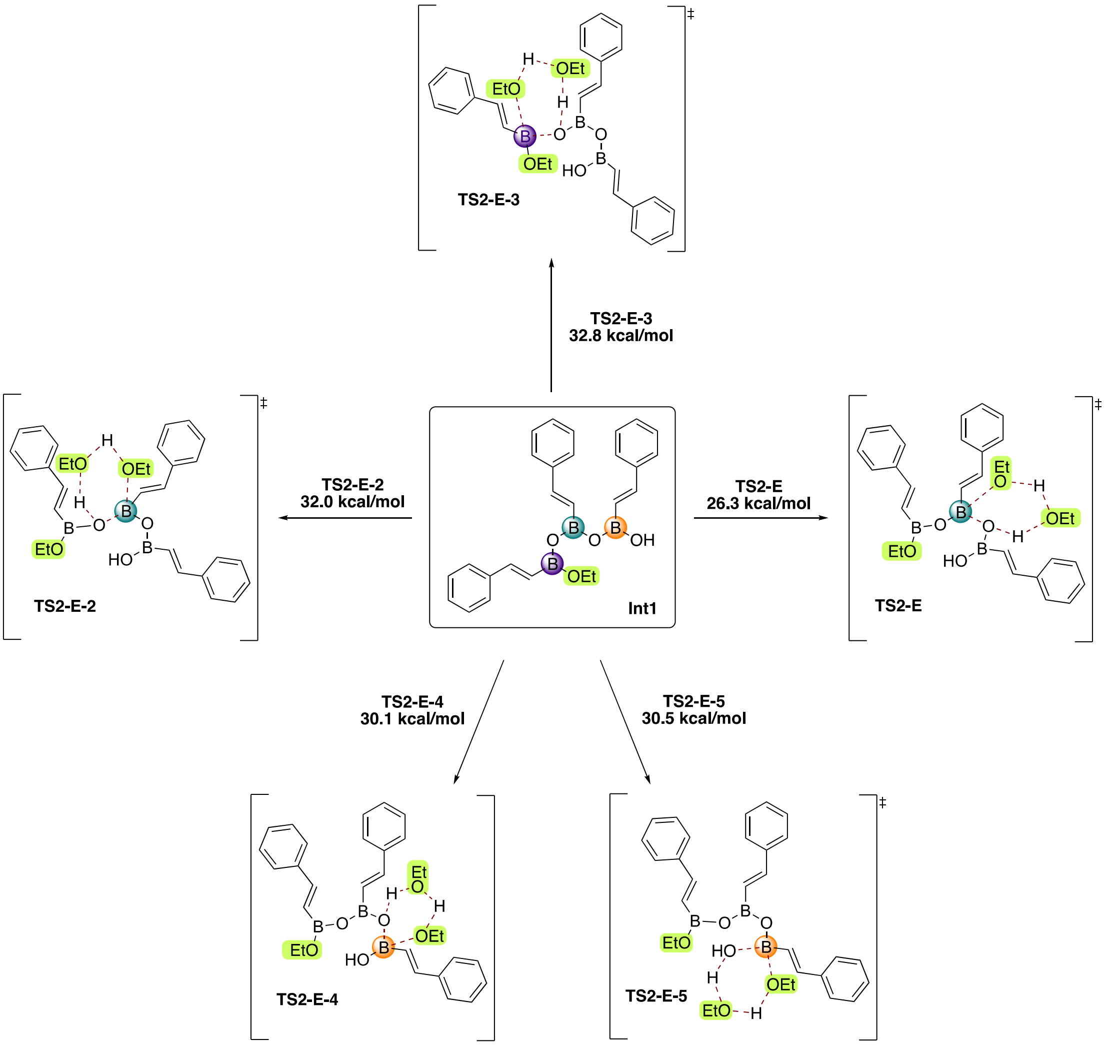

Scheme S1. *Alternative pathways for the esterification of* ***Int1-E*** *with ethanol.*

5. Formation of Int7 by the opening of boroxine 1 with (*R*)-I-BINOL 3

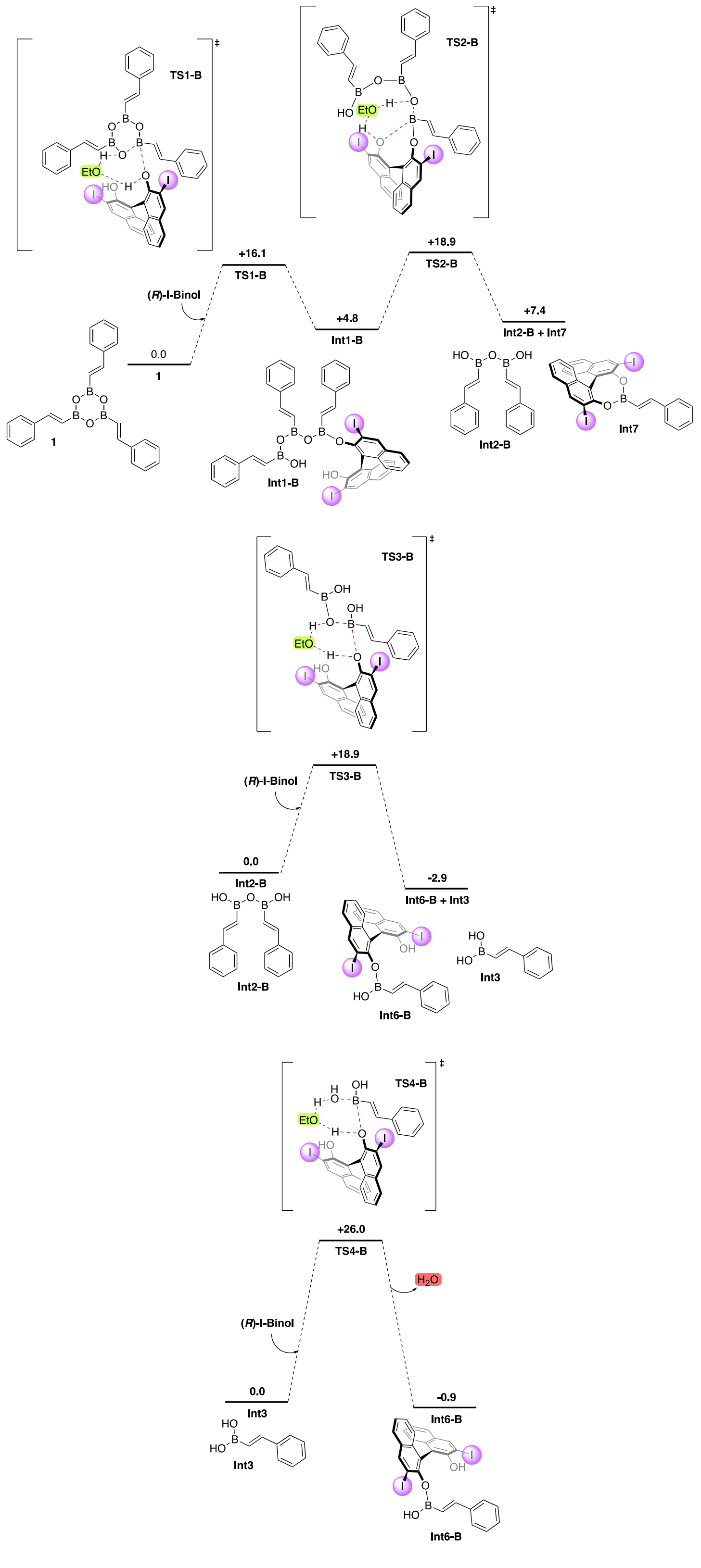

Figure S4-1. *Calculated free energy profiles (kcal/mol) for the formation of* ***Int7*** *by the opening of the boroxine* ***1*** *with (R)-I-BINOL* ***3****.*

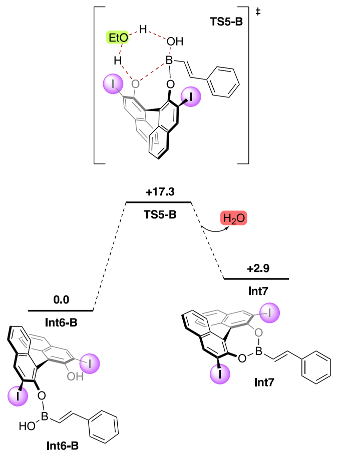

Figure S4-2. *Calculated free energy profiles (kcal/mol) for the formation of* ***Int7*** *by the opening of the boroxine* ***1*** *with (R)-I-BINOL* ***3****.*

6. Formation of boroxine 1 from Int3

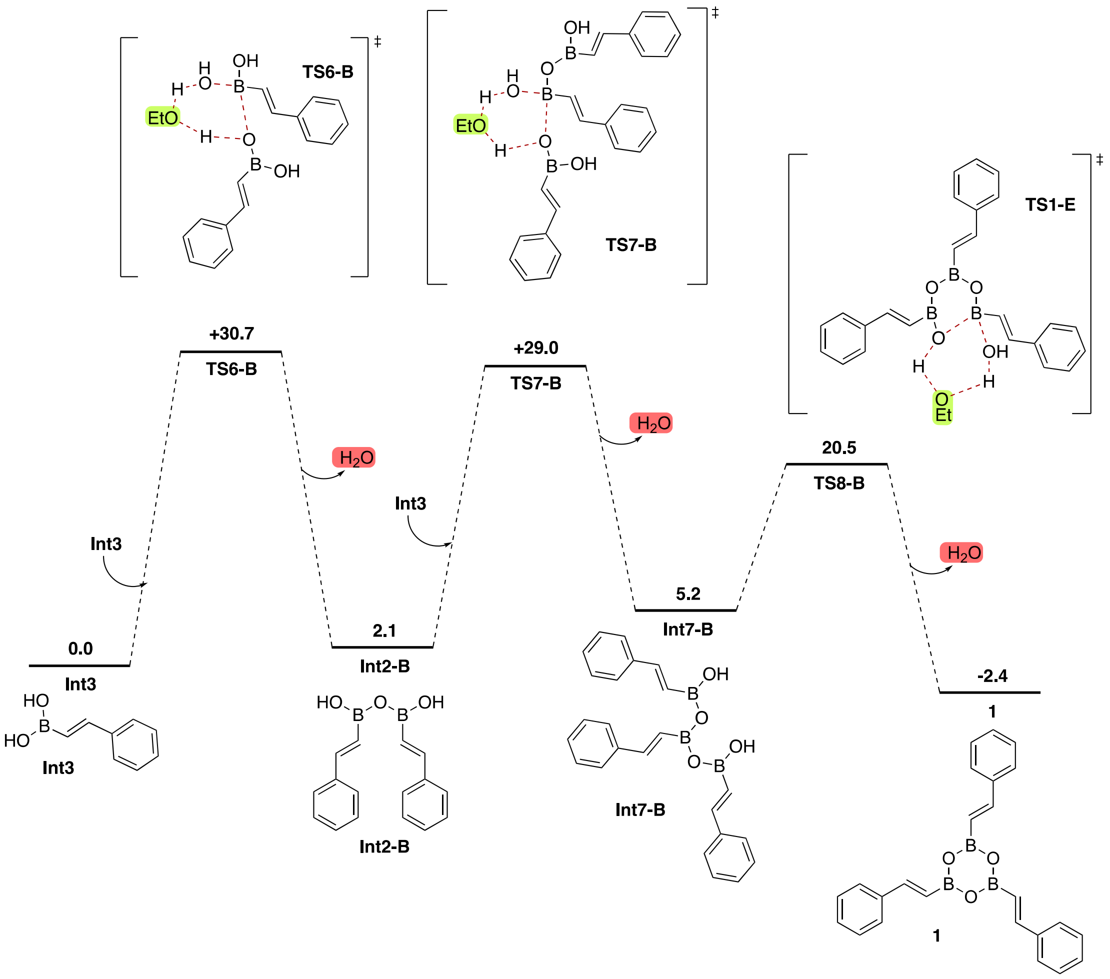

Figure S5. *Calculated free energy profile (kcal/mol) for the formation of **1** from **Int3**.*

7. Hydrolysis of 4(*S*) to 4-2OH(*S*)

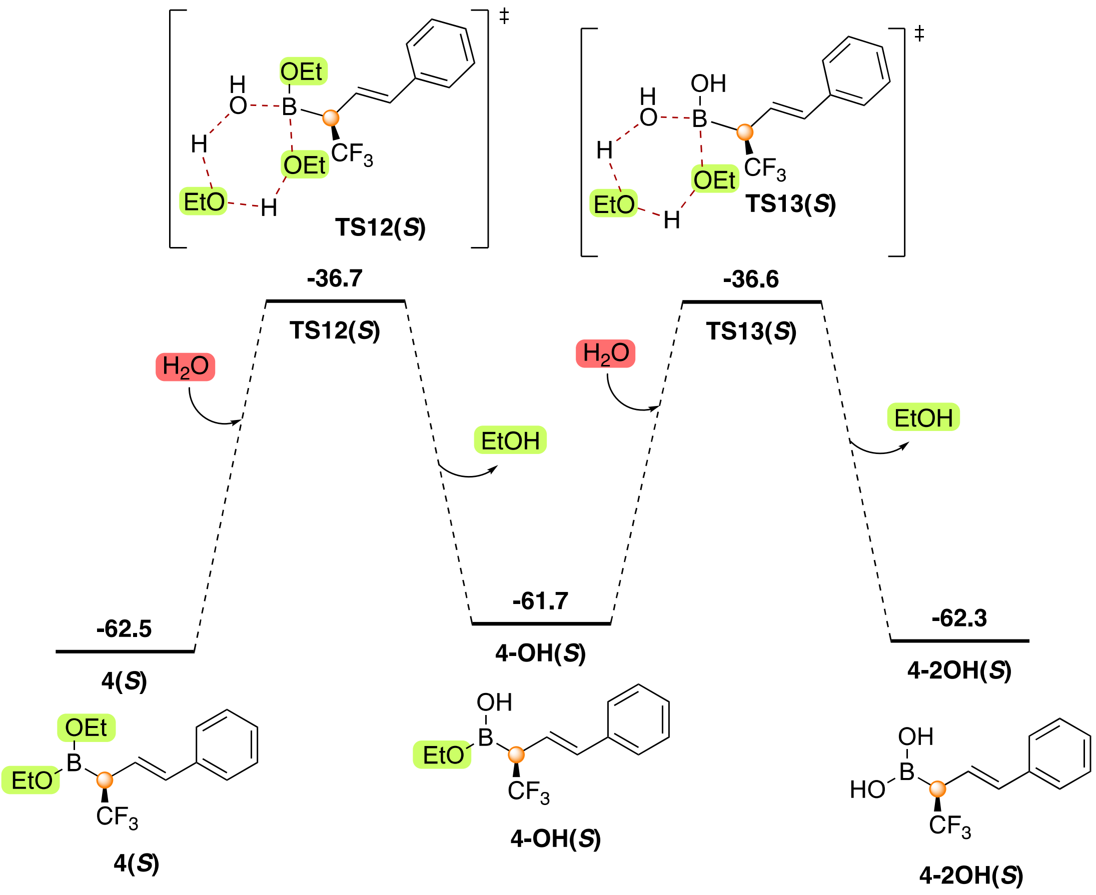

Figure S6. *Calculated free energy profile (kcal/mol) for the hydrolysis of* ***4(S)*** *to the final product* ***4-2OH(S)****.*

8. Optimized transition states for 1,2-migratory insertion

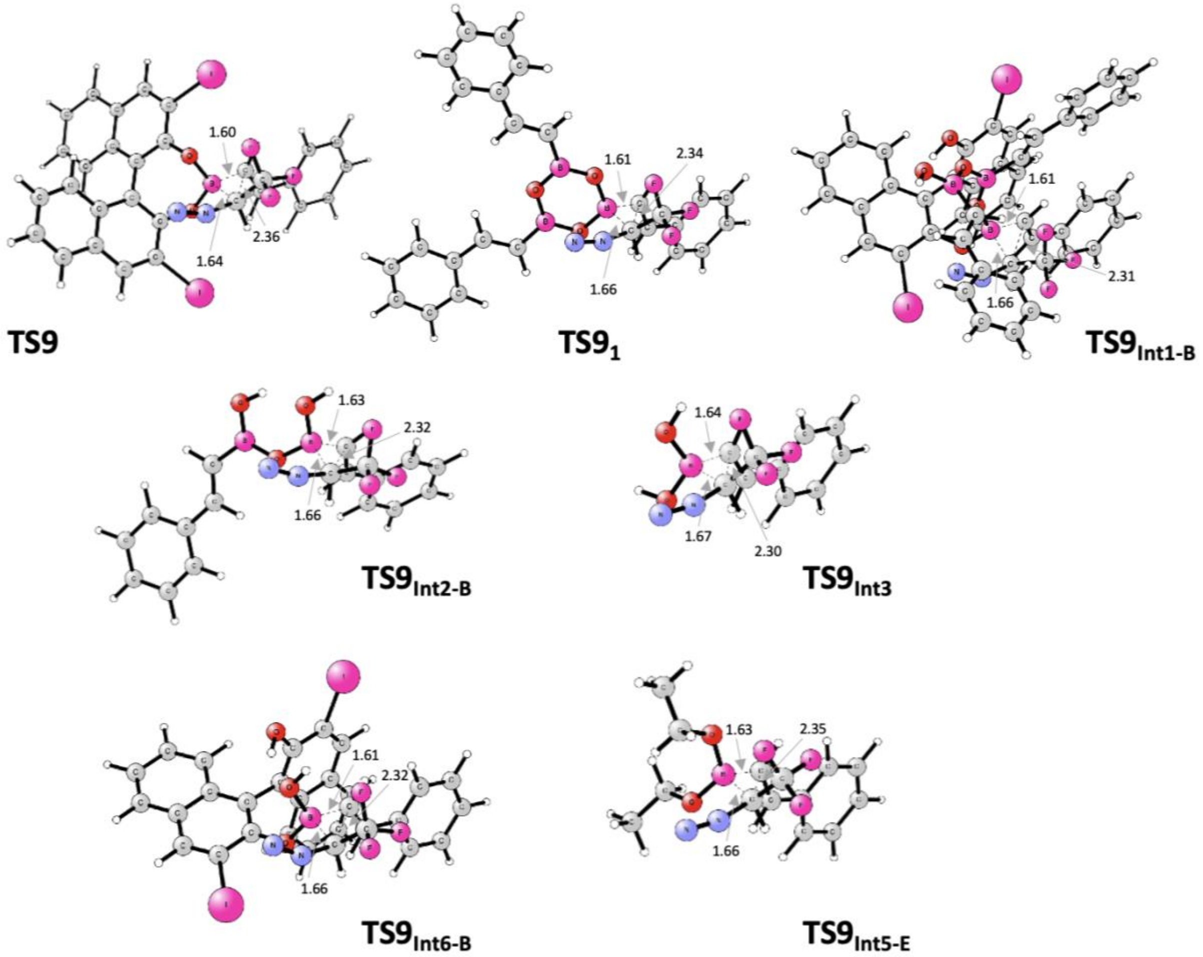

Figure S7. *Optimized TSs for the 1,2-migratory insertion step (**TS9**) of the key organoboron compounds (**Int7**, **1**, **Int1-B**, **Int2-B**, **Int3**, **Int6-B** and **Int5-E**). Selected bond distances are indicated in Å.*

9. Details of the kinetic network used in the simulations

Table S1 contains the list of the reactions constituting the kinetic network used in the simulations, along with the barriers for the forward and backward directions, and the corresponding rate constants calculated using the Eyring equation, at 40 °C (313.15 K):

$$k = \frac{k_B T}{h} e^{\frac{-\Delta G^{\neq}}{RT}}$$

Initial concentrations used in the simulations are based on the experimental data:

[**1**]$_0$ = 0.041 M
[**EtOH**]$_0$ = 0.25 M
[(***R***)-**I-BINOL**]$_0$ = 0.025 M
[**2a**]$_0$ = 0.38 M

Table S1. *Details of the kinetic network used in the simulations.*

Entry	Reaction	ΔG forward (kcal/mol)	ΔG backward (kcal/mol)	k forward[a]	k backward[a]
1	**1** + EtOH + EtOH ⇌ **Int1-E** + EtOH	20.6	13.9	2.73E-02	1.30E+03
2	**Int1-E** + EtOH + EtOH ⇌ **Int2-E** + **Int3** + EtOH	19.6	24.6	0.14E+00	4.41E-05
3[b]	**Int3** + EtOH + EtOH ⇌ **Int4-E** + EtOH + H_2O	27.9	-	2.19E-07	-
4[b]	**Int4-E** + EtOH + EtOH ⇌ **Int5-E** + EtOH + H_2O	29.4	-	1.97E-08	-
5	**Int2-E** + EtOH + EtOH ⇌ **Int4-E** + **Int5-E** + EtOH	28.0	28.4	1.87E-07	9.83E-08
6	**Int5-E** + **3** + **EtOH** ⇌ **Int6-E** + EtOH + EtOH	24.2	25.2	8.39E-05	1.68E-05
7	**Int6-E** + EtOH ⇌ **Int7** + EtOH + EtOH	16.6	12.1	1.70E+01	2.34E+04
8	**Int7** + **2** ⇌ **Int8**	16.3	1.4	2.74E+01	6.88E+11
9	**Int8** ⇌ **Int9** + N_2	8.0	80.3	1.70E+07	5.86E-44
10	**Int9** + EtOH + EtOH ⇌ **Int10** + EtOH	10.9	16.3	1.61E+05	2.74E+01
11	**Int10** + EtOH + EtOH ⇌ **4(*S*)** + EtOH + **3**	24.8	24.5	3.20E-05	5.18E-05
12	**1** + **3** + EtOH ⇌ **Int1-B** + EtOH	16.1	11.3	3.78E+01	8.46E+04
13	**Int1-B** + EtOH ⇌ **Int7** + **Int2-B** + EtOH	14.1	11.5	9.40E+02	6.14E+04
14	**Int2-B** + **3** + EtOH ⇌ **Int3** + **Int6-B** + EtOH	18.9	21.8	4.20E-01	3.97E-03
15[b]	**Int3** + **3** + EtOH ⇌ **Int6-B** + EtOH + H_2O	26.0	-	4.65E-06	-
16[b]	**Int6-B** + EtOH ⇌ **Int7** + EtOH + H_2O	17.4	-	4.68E+00	-
17[b]	**Int3 + Int3** ⇌ **Int2-B** + H_2O	30.7	-	2.44E-09	-
18[b]	**Int2-B + Int3** ⇌ **Int2-B** + H_2O	26.9	-	1.09E-06	-
19[b]	**Int7-B** ⇌ **1** + H_2O	15.3	-	1.36E+02	-

[a] Rate constants are given in $s^{-1}M^{-n}$, where n = (total order of the reaction – 1).

[b] Reactions producing water are assumed to be irreversible due to the presence of molecular sieves in the reaction mixture.

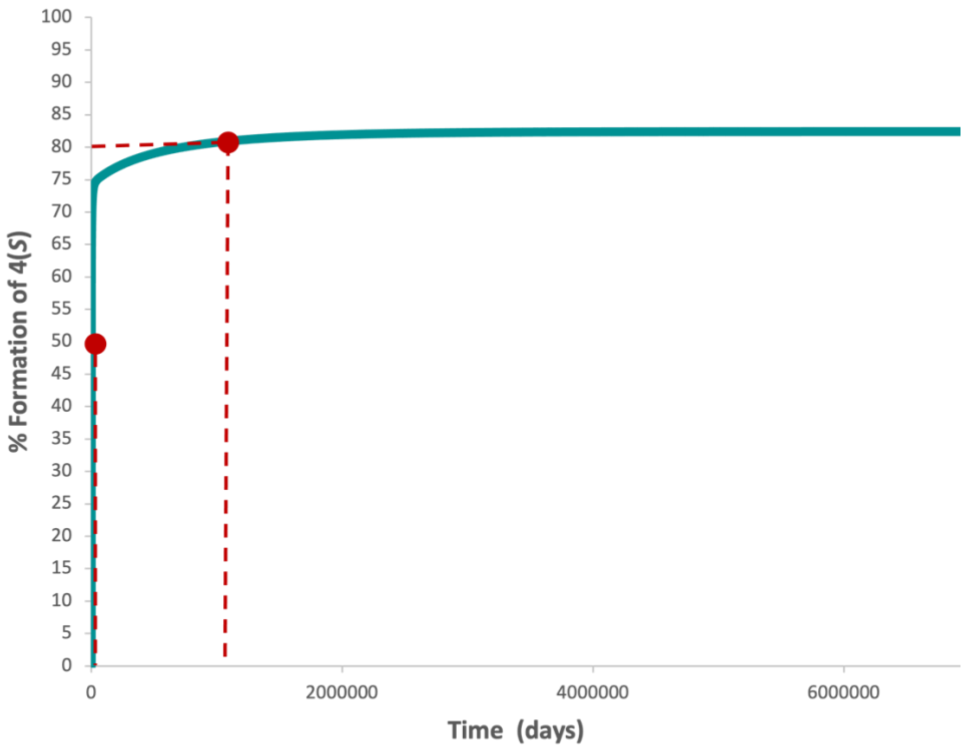

Figure S8. *Formation of **4(S)** vs time.*

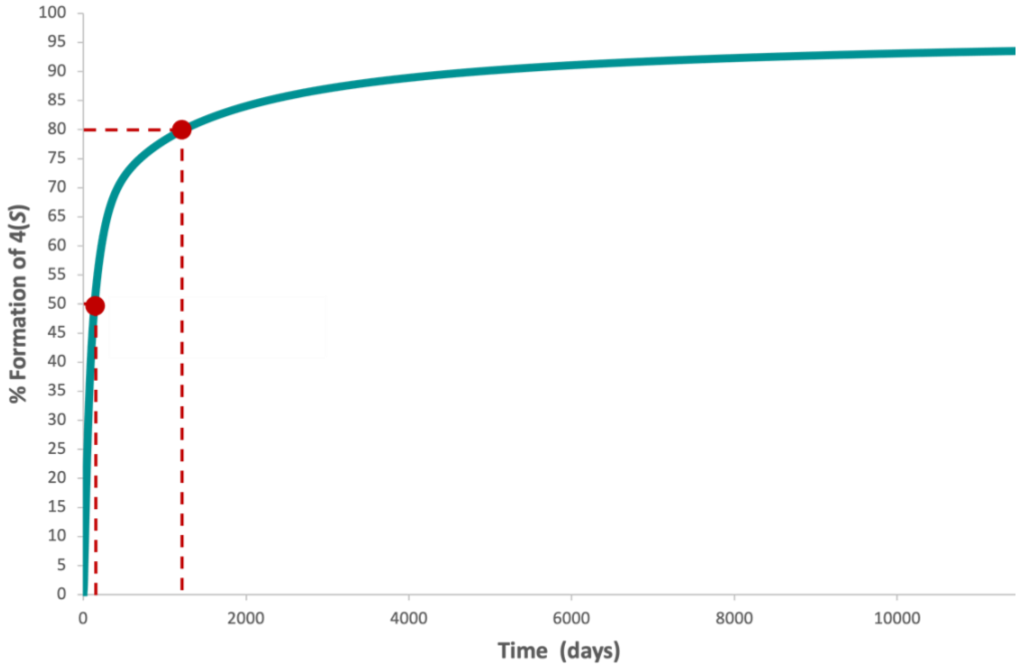

Figure S9. *Formation of **4(S)** vs time, assuming that the final step of reaction, **Int10(S)** to **4(S)**, is irreversible.*

Sensitivity analysis was conducted by increasing and decreasing the barriers of the elementary reactions by 1.4 or 2.8 kcal/mol. The time for reaching 50% formation of product **4(*S*)** was monitored and is listed in Table S2.

Table S2. *Results of sensitivity analysis.*

Reaction	Barrier change	Time (s) at 50% of formation of 4(*S*)	Relative time
Unmodified		19084500	1.0
TS1-E	+1.4	19084500	1.0
	+2.8	19084500	1.0
	-1.4	19084500	1.0
	-2.8	19084500	1.0
TS2-E	+1.4	19229000	1.0
	+2.8	18839500	1.0
	-1.4	19098500	1.0
	-2.8	18812500	1.0
TS4-E	+1.4	18770500	1.0
	+2.8	18737500	1.0
	-1.4	22127500	1.2
	-2.8	41786000	2.2
TS5-E	+1.4	19085000	1.0
	+2.8	19077000	1.0
	-1.4	19085500	1.0
	-2.8	19013000	1.0
TS3-E	+1.4	19066000	1.0
	+2.8	19064000	1.0
	-1.4	19239500	1.0
	-2.8	19764000	1.0
TS6-E	+1.4	10465000	0.5
	+2.8	9491000	0.5
	-1.4	13498000	0.7
	-2.8	12419500	0.7

Table S2. *Continued.*

Reaction	Barrier change	Time (s) at 50% of formation of 4(*S*)	Relative time
TS7-E	+1.4	19101500	1.0
	+2.8	19084500	1.0
	-1.4	19085000	1.0
	-2.8	19084500	1.0
TS8	+1.4	19081000	1.0
	+2.8	19234500	1.0
	-1.4	19077500	1.0
	-2.8	19083500	1.0
TS9	+1.4	57858500	3.0
	+2.8	257250000	13.5
	-1.4	5818000	0.3
	-2.8	3986500	0.2
TS10	+1.4	19084500	1.0
	+2.8	19084500	1.0
	-1.4	19084500	1.0
	-2.8	19084500	1.0
TS11	+1.4	73022500	3.8
	+2.8	747650000	39.2
	-1.4	13354000	0.7
	-2.8	12412500	0.7
TS1-B	+1.4	19084500	1.0
	+2.8	19084500	1.0
	-1.4	19084500	1.0
	-2.8	19084500	1.0
TS2-B	+1.4	19080500	1.0
	+2.8	19044000	1.0
	-1.4	19085000	1.0
	-2.8	19085000	1.0

Table S2. *Continued.*

Reaction	Barrier change	Time (s) at 50% of formation of 4(*S*)	Relative time
TS3-B	+1.4	19175000	1.0
	+2.8	29827500	1.6
	-1.4	19489000	1.0
	-2.8	19631000	1.0
TS4-B	+1.4	20920000	1.1
	+2.8	21202000	1.1
	-1.4	14723500	0.8
	-2.8	12749500	0.7
TS5-B	+1.4	19084500	1.0
	+2.8	19084500	1.0
	-1.4	19084500	1.0
	-2.8	19084500	1.0
TS6-B	+1.4	19112000	1.0
	+2.8	19115000	1.0
	-1.4	18835000	1.0
	-2.8	17174000	0.9
TS7-B	+1.4	19085500	1.0
	+2.8	19085500	1.0
	-1.4	19075000	1.0
	-2.8	18983500	1.0
TS8-B	+1.4	19084500	1.0
	+2.8	19084500	1.0
	-1.4	19084500	1.0
	-2.8	19084500	1.0

To find out where the final product **4(*S*)** stems from, the kinetic network was split into six pathways deriving from the six different **Int7** intermediates as shown in the figure below. Simulations where run to 50% formation of the product and the origins of the product with respect to the different **Int7** intermediates are listed in Table S3.

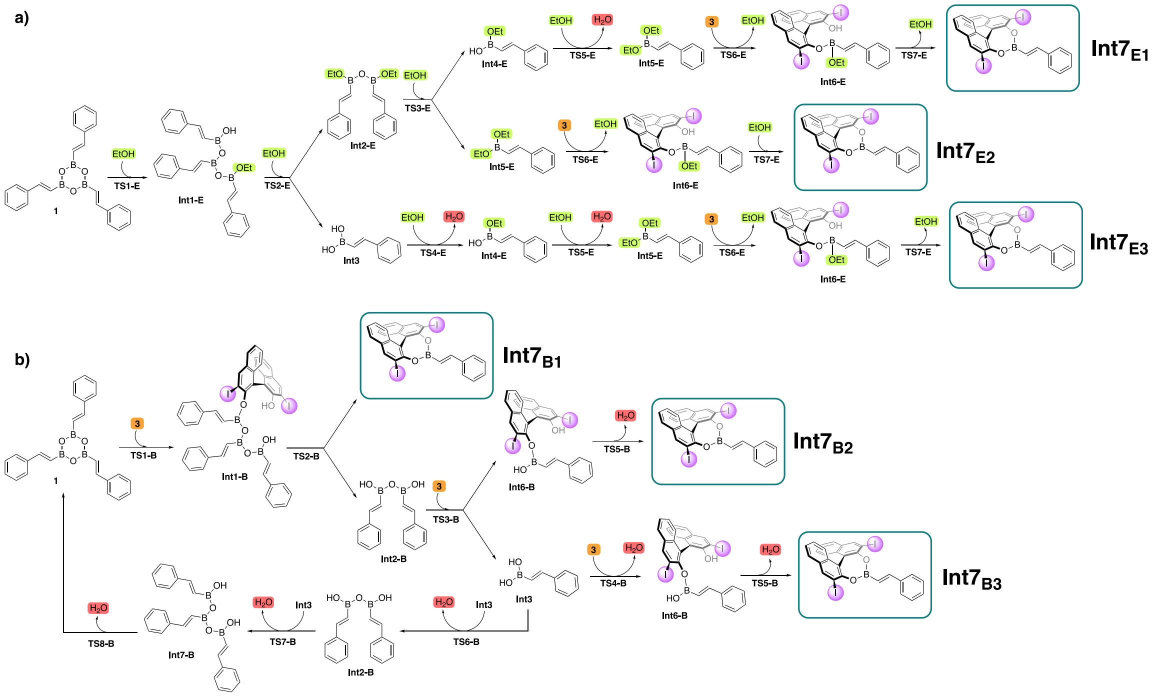

***Table S3**. Origins of product **4(S)** at 50% formation.*

Intermediate	4(*S*) (%)
**Int7$_{B1}$**	49.71
**Int7$_{B2}$**	49.69
**Int7$_{B3}$**	0.58
**Int7$_{E1}$**	0.00
**Int7$_{E2}$**	0.00
**Int7$_{E3}$**	< 0.02

10. Absolute energies and energy corrections

Table S4. *Absolute energies and energy corrections.*

Stationary point	Electronic energy 6-31G(d,p)/LANL2DZ (a.u.)	Thermal correction to Gibbs free energy (a.u)	Single-point energy 6-311+G(2d,2p)/LANL2DZ (a.u.)
**1**	-1227.91417	0.33327	-1228.24854
**TS1-E**	-1538.05931	0.47840	-1538.48610
**TS1-E-2**	-1382.94859	0.40488	-1383.32748
**Int1-E**	-1382.98955	0.40716	-1383.37443
**TS2-E**	-1693.14046	0.55322	-1693.61459
**TS2-E-2**	-1693.13141	0.55471	-1693.60689
**TS2-E-3**	-1693.13174	0.55516	-1693.60615
**TS2-E-4**	-1693.13440	0.55367	-1693.60899
**TS2-E-5**	-1693.13666	0.55475	-1693.60939
**Int2-E**	-1052.31434	0.33828	-1052.60483
**Int3**	-485.73865	0.11831	-485.88958
**TS3-E**	-1362.45370	0.48624	-1362.83344
**TS4-E**	-795.87177	0.26201	-796.11418
**Int4-E**	-564.37134	0.17011	-564.53723
**TS5-E**	-874.50741	0.31638	-874.76203
**Int5-E**	-643.00381	0.22153	-643.18428
**TS6-E**	-1740.79523	0.52918	-1741.29694
**Int6-E**	-1430.65313	0.37853	-1431.06121
**TS7-E**	-1585.71759	0.45148	-1586.17031
**Int7**	-1275.56245	0.30485	-1275.91756
**TS1-B**	-2325.70423	0.64021	-2326.37340
**Int1-B**	-2170.63296	0.56988	-2171.25845
**TS2-B**	-2325.69757	0.64052	-2326.36923
**Int2-B**	-895.04890	0.23693	-895.30874
**TS3-B**	-1992.84512	0.54512	-1993.43030
**TS4-B**	-1583.52433	0.42243	-1583.99588
**Int6-B**	-1352.02227	0.32783	-1352.41394
**TS5-B**	-1507.08598	0.40049	-1507.52155
**TS6-B**	-1126,55903	0,33833	-1126,89463
**TS7-B**	-1535,86826	0,45097	-1.536,31381
**Int7-B**	-1.304,35267	0,35282	-1.304,72349
**TS8-B**	-1.459,42366	0,42721	-1.459,83606
**TS8(*S*)**	-1761.34786	0.33457	-1761.87391
**TS8(*R*)**	-1761.34480	0.33383	-1761.87059
**Int8(*S*)**	-1761.34805	0.33255	-1761.87409

Table S4. *Continued.*

Stationary point	Electronic energy 6-31G(d,p)/LANL2DZ (a.u.)	Thermal correction to Gibbs free energy (a.u)	Single-point energy 6-311+G(2d,2p)/LANL2DZ (a.u.)
**Int8(*R*)**	-1761.34557	0.33409	-1761.87125
**TS9(*S*)**	-1761.33535	0.33275	-1761.86153
**TS9(*R*)**	-1761.33032	0.33306	-1761.85635
**$TS9_{Int3}$**	-971.48805	0.14330	-971.80426
**$TS9_{Int2\text{-}B}$**	-1380.80896	0.26416	-1381.23331
**$TS9_{Int6\text{-}B}$**	-1837.78175	0.35708	-1838.3401
**$TS9_{Int1\text{-}B}$**	-2656.39211	0.59727	-2657.18441
**$TS9_{1}$**	-1713.67965	0.35978	-1714.17904
**Int9(*S*)**	-1651.92140	0.32837	-1652.41272
**Int9(*R*)**	-1651.91982	0.32984	-1652.41144
**TS10(*S*)**	-1962.09150	0.48397	-1962.67613
**Int10(*S*)**	-1807.02359	0.40865	-1807.56420
**TS11(*S*)**	-2117.16642	0.55785	-2117.79912
**4(*S*)**	-1 019.36988	0.24640	-1 019.68317
**TS12(*S*)**	-1250.88270	0.34195	-1251.26767
**4-OH(*S*)**	-940.73806	0.19533	-941.03565
**TS13(*S*)**	-1172.25009	0.29214	-1172.62243
**4-2OH(*S*)**	-862.10349	0.14019	-862.38613

11. Cartesian coordinates

1

C	-3.79561100	-0.48008300	0.00012700
H	-3.68980300	0.60464400	0.00044600
C	-2.66694000	-1.22064100	-0.00022800
H	-2.73901000	-2.30761400	-0.00051800
C	-5.18304600	-0.95254100	0.00010300
C	-6.22020100	-0.00252800	0.00096400
C	-5.52725200	-2.31803700	-0.00076600
C	-7.55639600	-0.39823600	0.00101200
H	-5.96690900	1.05410100	0.00161200
C	-6.86086300	-2.71281500	-0.00072500
H	-4.74637500	-3.07151800	-0.00151400
C	-7.88125400	-1.75554800	0.00017500
H	-8.34242600	0.35093700	0.00168900
H	-7.10924100	-3.76995900	-0.00140700
H	-8.92106300	-2.06817200	0.00019700
B	-1.26696300	-0.57636300	-0.00029700
O	-1.12024000	0.80134500	0.00038200
B	0.13343700	1.38429200	0.00021700
O	1.25326800	0.56838200	-0.00004600
O	-0.13529300	-1.37069100	-0.00072300
B	1.13120100	-0.80885000	-0.00030600
C	2.38958000	-1.69873400	-0.00011300
H	3.36653500	-1.21662500	-0.00011300
C	0.27538800	2.91896200	0.00036000
H	-0.63028900	3.52450900	0.00060900
C	1.48146100	3.52610800	0.00002500
H	2.36788800	2.89197100	-0.00029900
C	2.31360900	-3.04672500	0.00003600
H	1.32173400	-3.49833000	-0.00039100
C	3.41702500	-4.01117500	0.00032100
C	3.11366500	-5.38460000	-0.00265700
C	4.77124700	-3.62570500	0.00345000
C	4.12496800	-6.34339600	-0.00277900
H	2.07212100	-5.69420400	-0.00498900
C	5.78048100	-4.58267700	0.00339500
H	5.03292100	-2.57262600	0.00611500
C	5.46259700	-5.94520700	0.00022400
H	3.86972900	-7.39879400	-0.00518000
H	6.81997200	-4.26858800	0.00589900
H	6.25384100	-6.68882800	0.00019500
C	1.76632300	4.96371800	-0.00009100
C	3.10772000	5.38661700	-0.00179500
C	0.75617600	5.94468400	0.00139100
C	3.43350100	6.74155700	-0.00210600
H	3.89595300	4.63861700	-0.00290900
C	1.08142800	7.29694300	0.00111700
H	-0.28694000	5.64570800	0.00283200
C	2.42066800	7.70180200	-0.00064700
H	4.47537200	7.04728900	-0.00346400
H	0.29016500	8.04074000	0.00230600
H	2.66992600	8.75860400	-0.00085100

TS1-E

C	-1.14313400	3.26085400	0.50028600
H	-1.88866200	2.68904100	-0.05211900
C	-0.06568700	2.59802500	0.96686100
H	0.69634800	3.14425800	1.52334400
B	0.14966700	1.07749200	0.73509700
O	-0.82992600	0.36674300	0.02733400
B	-0.78287200	-1.00007100	-0.07159000
O	0.36151800	-1.66335900	0.33271900
O	1.24614900	0.45228500	1.20929000
B	1.60956200	-0.91736900	0.89281100
C	2.78104800	-1.02343900	-0.18995900
H	3.08815900	-2.02878800	-0.49406500
C	-1.98819600	-1.81671000	-0.59330900
H	-1.88039000	-2.89420400	-0.71996100
C	-3.16801300	-1.23297300	-0.88977300
H	-3.24525100	-0.15405200	-0.75670000
C	3.40775700	0.02425300	-0.75062000
H	3.09775300	1.02405900	-0.44400700
O	1.89359700	-1.69224200	2.13175200
C	3.08060700	-1.37082000	2.85485700
H	3.07816700	-0.29749100	3.08182100
H	3.96458500	-1.57254600	2.23450200
H	1.28200600	-3.09619200	2.00630100
O	0.56668400	-3.68664600	1.55386400
H	0.33445700	-2.83378400	0.81532300
C	-0.55644400	-3.84257800	2.48016000
H	-0.82024400	-2.85522200	2.87194000
H	-1.38636000	-4.21744400	1.87824000
C	3.12526600	-2.18494100	4.13437900
H	3.15195000	-3.25791800	3.91365800
H	4.02096100	-1.93630100	4.71285100
H	2.24665100	-1.97897900	4.75447300

C	-0.18059500	-4.80462600	3.58437700
H	0.65575800	-4.41856800	4.17557700
H	-1.03549400	-4.94067200	4.25402600
H	0.09775100	-5.77952300	3.17404100
C	-4.39342400	-1.87082900	-1.38027300
C	-5.51800900	-1.06543900	-1.63424100
C	-4.49825300	-3.25600300	-1.60992400
C	-6.70777300	-1.61977600	-2.10255900
H	-5.44898100	0.00498000	-1.46054600
C	-5.68572300	-3.80943400	-2.07712300
H	-3.64592600	-3.90088300	-1.42209300
C	-6.79559700	-2.99464300	-2.32571000
H	-7.56427200	-0.98020500	-2.29329400
H	-5.74944500	-4.87966300	-2.24988300
H	-7.72043200	-3.43085400	-2.69081300
C	-1.45823100	4.68769100	0.63681100
C	-2.65524500	5.17196100	0.07940600
C	-0.61667300	5.60029400	1.30115600
C	-3.00417400	6.51758300	0.18070300
H	-3.31331600	4.47846300	-0.43745800
C	-0.96440800	6.94354100	1.40233900
H	0.31455200	5.25538900	1.73862000
C	-2.15935100	7.40918800	0.84338900
H	-3.93348500	6.86965600	-0.25717600
H	-0.30237800	7.63295600	1.91805400
H	-2.42667400	8.45847600	0.92442500
C	4.48757100	0.00808000	-1.75119800
C	4.99234700	1.23080000	-2.22844100
C	5.04886200	-1.17892800	-2.25919000
C	6.01550000	1.27179200	-3.17496300
H	4.57108300	2.15739200	-1.84693600
C	6.07009400	-1.13991100	-3.20409600
H	4.68279300	-2.13885800	-1.90849900
C	6.55992200	0.08527400	-3.66849700
H	6.38682900	2.23001900	-3.52697500
H	6.48817500	-2.06884500	-3.58164700
H	7.35710900	0.11209300	-4.40538600

TS1-E-2

C	-1.08490500	2.89468100	0.08167500
H	-1.82937100	2.23853700	-0.36876500
C	-0.00224800	2.32162300	0.64685500
H	0.75969100	2.95380400	1.10234300
C	-1.40359700	4.32306500	-0.01257600
C	-2.59070400	4.70933400	-0.66021300
C	-0.57466400	5.33022000	0.51755800
C	-2.94103600	6.05313200	-0.77811500
H	-3.23917400	3.94115000	-1.07303000
C	-0.92434100	6.67145800	0.40033900
H	0.34638300	5.06020100	1.02390700
C	-2.10818900	7.03963900	-0.24799600
H	-3.86178200	6.32967400	-1.28302700
H	-0.27325300	7.43529700	0.81508500
H	-2.37699700	8.08783600	-0.33750800
B	0.21095200	0.78967000	0.67349000
O	-0.75502400	-0.04056900	0.09849700
B	-0.73625000	-1.40549000	0.22484600
O	0.44786700	-2.01003000	0.67034200
O	1.31511700	0.25967800	1.26428900
B	1.68776300	-1.10802000	1.19796600
C	3.00815200	-1.45168500	0.41411700
H	3.33187100	-2.49412300	0.39394200
C	-1.96667900	-2.27127400	-0.09838200
H	-1.88409600	-3.35399600	-0.01175700
C	-3.13921900	-1.72223100	-0.48320100
H	-3.18834000	-0.63650300	-0.56228600
C	3.74978800	-0.52970800	-0.22324500
H	3.41289300	0.50676300	-0.18790900
C	4.99342000	-0.73567200	-0.98136500
C	5.61982100	0.37505300	-1.57367200
C	5.59258800	-1.99874600	-1.14576600
C	6.79970600	0.23473900	-2.30320700
H	5.16955200	1.35726300	-1.45589900
C	6.77023300	-2.14012800	-1.87375300
H	5.13238200	-2.87481900	-0.69989500
C	7.38080900	-1.02477300	-2.45673000
H	7.26453700	1.10817500	-2.75128000
H	7.21598700	-3.12403800	-1.98852300
H	8.29973600	-1.13893000	-3.02386300
C	-4.38780000	-2.40995300	-0.81970900
C	-5.49566300	-1.64062000	-1.21854300
C	-4.53033700	-3.80960300	-0.76137400
C	-6.70659400	-2.24497100	-1.55029800
H	-5.39636700	-0.55965800	-1.26677200
C	-5.73909100	-4.41246200	-1.09225700
H	-3.69130300	-4.42554200	-0.45441800
C	-6.83195900	-3.63364200	-1.48829700
H	-7.55041900	-1.63403400	-1.85631400
H	-5.83336800	-5.49307400	-1.04218600
H	-7.77371600	-4.10883100	-1.74565100

O	1.57646400	-1.86989400	2.52858600
C	1.17006400	-1.17339200	3.72494100
H	0.24428600	-0.61680100	3.53731200
H	1.95182900	-0.44831900	3.97107800
C	0.98298100	-2.18083500	4.84082900
H	1.90920800	-2.73377900	5.02429300
H	0.69726400	-1.66635100	5.76397300
H	0.19429400	-2.89832000	4.59083200
H	0.63733200	-2.34534200	1.76540500

Int1-E

C	-0.81928143	2.84519172	1.36172074
H	-1.25108106	2.17602461	2.10608491
C	-0.07469800	2.29457800	0.38115800
H	0.37288957	2.93985820	-0.37455265
C	-1.13355443	4.26281199	1.56892584
C	-1.91737366	4.62610630	2.67863999
C	-0.68605514	5.28174172	0.70634956
C	-2.24392567	5.95878517	2.92323665
H	-2.26965123	3.84858036	3.35117579
C	-1.01208587	6.61191872	0.95007780
H	-0.08160413	5.02930553	-0.15883128
C	-1.79183447	6.95723402	2.05920212
H	-2.85042477	6.21727235	3.78611596
H	-0.65879597	7.38500065	0.27409143
H	-2.04407769	7.99674367	2.24557551
B	0.18251200	0.76839100	0.28977400
O	-0.68595900	-0.01735600	-0.43554300
B	-1.80416747	0.28956924	-1.17890098
O	-2.45452103	-0.75080785	-1.78736036
O	1.24646900	0.24123800	0.96309400
B	2.28172303	0.69561332	1.75801822
C	2.35008393	2.19127512	2.16463033
H	3.04610625	2.83886945	1.63127191
C	-2.27201147	1.76415225	-1.28870915
H	-2.03948618	2.32999251	-2.19167285
C	-2.97061737	2.36525326	-0.30324698
H	-3.18603451	1.77978890	0.59053451
C	1.58879845	2.71014633	3.14802950
H	0.90136295	2.04002274	3.66439775
C	1.55840121	4.09554953	3.63111287
C	0.67252458	4.43053654	4.67070242
C	2.37773660	5.11106023	3.10211344
C	0.60324160	5.73138700	5.16654234
H	0.03460932	3.65599520	5.08806897
C	2.30920514	6.40951706	3.59685017
H	3.07177962	4.88091698	2.30027445
C	1.42205322	6.72667956	4.63105048
H	-0.08840038	5.96754246	5.96977222
H	2.94947857	7.17961606	3.17657250
H	1.37182624	7.74128611	5.01459196
C	-3.49333813	3.73494904	-0.27012136
C	-4.18146264	4.16603291	0.87820260
C	-3.34071234	4.64181925	-1.33638592
C	-4.69876762	5.45724827	0.96411525
H	-4.30656117	3.47510220	1.70750124
C	-3.85725861	5.93053325	-1.25085046
H	-2.81713143	4.33614726	-2.23644409
C	-4.53815353	6.34462660	-0.10098544
H	-5.22630886	5.76995662	1.86020579
H	-3.73073415	6.61706714	-2.08268241
H	-4.93988571	7.35142531	-0.03891710
O	3.21321570	-0.14703262	2.29243915
C	3.17905956	-1.57158568	2.11928244
H	4.18894818	-1.92676885	2.34558703
H	2.96955073	-1.82761264	1.07686261
C	2.16362260	-2.21644788	3.04777087
H	1.14867259	-1.88794877	2.80492625
H	2.20457930	-3.30648980	2.94977090
H	2.37367775	-1.95530385	4.08977706
H	-3.24864117	-0.46822410	-2.26233947

TS2-E

C	2.99929000	-1.18360100	0.18531900
H	2.83226900	-0.38775600	0.91148500
C	1.91712800	-1.83877800	-0.26922700
H	2.06268000	-2.63472400	-1.00517200
C	4.41022400	-1.37904800	-0.18393400
C	5.37685500	-0.50375600	0.34313400
C	4.84826300	-2.40064500	-1.04775700
C	6.72656500	-0.63530100	0.01877200
H	5.05607100	0.28923100	1.01414000
C	6.19541600	-2.53393600	-1.37194700
H	4.12724200	-3.09681500	-1.46483800
C	7.14304900	-1.65205200	-0.84192100
H	7.45248600	0.05537400	0.43837700
H	6.51077300	-3.33057000	-2.03993000
H	8.19317400	-1.75955300	-1.09656900
B	0.41722600	-1.52654200	0.18997500
O	-0.45861500	-1.24885900	-1.06042600

B	-0.58928800	-0.04547100	-1.71930000
O	-1.65829400	0.01884700	-2.57364400
O	0.36983300	-0.42590900	1.12230300
B	-0.65462900	0.38553900	1.48292300
C	-2.14458100	0.09266300	1.10658500
H	-2.44226800	-0.93573800	0.91166300
C	0.40070300	1.12507200	-1.46460200
H	1.42393200	0.87561800	-1.19455100
C	0.01415100	2.41474700	-1.43434000
H	-1.02652100	2.65708900	-1.65587800
C	-3.05782300	1.06184900	0.90798800
H	-2.77120600	2.09218600	1.11917900
C	-4.43157100	0.89032000	0.41546500
C	-5.34150500	1.95548300	0.53377800
C	-4.87424300	-0.29999600	-0.19313700
C	-6.65601600	1.83184700	0.08625700
H	-5.00881500	2.88438100	0.98948200
C	-6.18603700	-0.42198000	-0.64199900
H	-4.18252800	-1.12454200	-0.33269300
C	-7.08470200	0.64055600	-0.50162200
H	-7.34410600	2.66530400	0.19357000
H	-6.50881400	-1.34613000	-1.11285500
H	-8.10631600	0.54220000	-0.85630300
C	0.83395700	3.57610500	-1.06610400
C	0.22624300	4.84084800	-0.98867800
C	2.20520800	3.47568700	-0.76215900
C	0.95678600	5.96865600	-0.61708000
H	-0.83226200	4.93154300	-1.21752800
C	2.93452500	4.60094400	-0.39195700
H	2.70191500	2.51235900	-0.81394900
C	2.31449800	5.85282800	-0.31637100
H	0.46600100	6.93572100	-0.56088700
H	3.99112500	4.50386100	-0.16030800
H	2.88690100	6.72839300	-0.02531100
O	-0.39871700	1.54679300	2.17324500
C	0.95311300	1.95870100	2.40239100
H	0.92990400	3.04773500	2.51729500
H	1.56618900	1.72602500	1.52675200
C	1.53251500	1.30198800	3.64501500
H	1.58306500	0.21691200	3.51672700
H	2.54498700	1.67527500	3.83588500
H	0.91481900	1.52042000	4.52251200
H	-1.69174100	0.85963600	-3.05036900
O	-0.25547900	-2.75923700	0.73766800
C	0.10859500	-3.19215800	2.04664300
H	1.20328500	-3.19919400	2.12368700
H	-0.26926800	-2.48032000	2.79150000
C	-0.45451300	-4.57884400	2.29615100
H	-0.17348500	-4.92842100	3.29486800
H	-1.54846200	-4.57268400	2.23584200
H	-0.06867600	-5.29176400	1.56013400
H	-1.48300100	-3.07113800	0.06038000
O	-2.13780400	-2.91749700	-0.75726900
H	-1.47773500	-2.07822900	-1.09719100
C	-2.07252100	-4.04854500	-1.67981800
H	-2.49525200	-3.69084800	-2.62070200
H	-1.02018900	-4.30537000	-1.83845200
C	-2.85796600	-5.20985400	-1.11344000
H	-3.90039400	-4.92644900	-0.94145000
H	-2.83727800	-6.04427700	-1.82135900
H	-2.42604100	-5.55427000	-0.16862400

TS2-E-2

C	2.88249500	-1.05140500	-0.42213000
H	2.61574100	-0.03643400	-0.71852900
C	2.08745800	-1.69911400	0.44638800
H	2.38128500	-2.71042900	0.73145100
C	4.12841500	-1.53934600	-1.03610600
C	4.93564400	-0.63790800	-1.75323200
C	4.56267800	-2.87504600	-0.93814100
C	6.13448700	-1.04502800	-2.33746700
H	4.61368900	0.39661500	-1.84480400
C	5.75895200	-3.28330400	-1.52149700
H	3.95084100	-3.59960500	-0.40987400
C	6.55434400	-2.37130800	-2.22294700
H	6.74020400	-0.32665800	-2.88277400
H	6.07195700	-4.32005300	-1.43405600
H	7.48610600	-2.69375900	-2.67794000
B	0.77147800	-1.10612900	1.14674700
O	-0.42048000	-1.90122700	1.07293800
B	-0.80911400	-2.93194300	0.29010000
O	0.11879400	-3.81039600	-0.23384500
O	0.46288900	0.30158300	0.58469200
B	-0.10560100	0.62557600	-0.63269500
C	-0.37899300	2.12620200	-0.95404000
H	-0.51034800	2.43168700	-1.99134300
C	-2.34073700	-3.14381200	0.02087000
H	-2.68231100	-4.02382400	-0.52885800
C	-3.25533000	-2.23447400	0.41053700
H	-2.88819100	-1.35549700	0.94012700

C	-0.48895000	3.07038600	0.00280000
H	-0.37239300	2.77326500	1.04390800
C	-0.74258100	4.50505700	-0.17301300
C	-0.69591200	5.33930000	0.95814600
C	-1.02080400	5.08960000	-1.42274000
C	-0.90921200	6.71224800	0.84768900
H	-0.48527700	4.89818800	1.92879600
C	-1.23593400	6.45960400	-1.53278200
H	-1.07442600	4.46733700	-2.31024700
C	-1.17977100	7.27766200	-0.39925000
H	-0.86623600	7.33908600	1.73333600
H	-1.45091300	6.89402700	-2.50461700
H	-1.34918700	8.34633100	-0.48974000
C	-4.70669600	-2.25055000	0.18756600
C	-5.45649500	-1.11381500	0.53885900
C	-5.38660200	-3.34702900	-0.37572100
C	-6.83362600	-1.06589200	0.32851100
H	-4.94391900	-0.26109100	0.97603900
C	-6.76163400	-3.30089500	-0.58373400
H	-4.83465900	-4.24154100	-0.64642700
C	-7.49214000	-2.16000000	-0.23476400
H	-7.39181700	-0.17600400	0.60424100
H	-7.26890100	-4.15747500	-1.01810600
H	-8.56498800	-2.12782500	-0.39922500
O	-0.37324200	-0.40088800	-1.48472500
C	-1.02091200	-0.21933200	-2.74875200
H	-0.79776400	-1.11824500	-3.33134100
H	-0.58296600	0.63359600	-3.27992400
C	-2.52187100	-0.04824000	-2.58976000
H	-2.75383300	0.85096300	-2.00970900
H	-2.99614700	0.04476600	-3.57270300
H	-2.95291300	-0.91045600	-2.07466000
H	-0.30585800	-4.49442800	-0.76845200
O	1.62498100	1.54971600	2.24024700
C	3.03050900	1.80245100	1.93806200
H	3.50238500	2.09437800	2.87934600
H	3.48515400	0.87198600	1.58603900
C	3.13089100	2.89760800	0.89861400
H	4.18518500	3.10842100	0.69465800
H	2.65368900	2.59679400	-0.03883400
H	2.65476900	3.81763100	1.25014000
H	1.06977500	1.15302900	1.32454300
H	1.50526100	0.59671200	2.68293200
O	1.03070400	-0.76681700	2.59993500
C	1.46460000	-1.80568400	3.47602600
H	0.90247800	-1.70635200	4.41291800
H	1.19670800	-2.77533600	3.04192000
C	2.95929200	-1.73067000	3.75256800
H	3.22359000	-0.76565200	4.19987500
H	3.25663300	-2.51907200	4.45312600
H	3.53520000	-1.85195300	2.83071800

TS2-E-3

C	-1.93870300	2.16449000	-0.36260800
H	-1.76460500	1.97568600	0.69626200
C	-1.18874500	1.49778300	-1.26459100
H	-1.37231300	1.66144600	-2.32646200
C	-2.99670500	3.14853000	-0.61793800
C	-3.59113800	3.79706200	0.47906600
C	-3.44225400	3.47951700	-1.91135700
C	-4.59097400	4.75035300	0.29469100
H	-3.25720000	3.54757100	1.48266800
C	-4.44136700	4.42995200	-2.09531900
H	-3.00777000	2.98703200	-2.77519400
C	-5.01979800	5.07050400	-0.99427000
H	-5.03541300	5.24118200	1.15533000
H	-4.77401300	4.67254800	-3.10021800
H	-5.79996000	5.81098200	-1.14251700
B	-0.14515800	0.41365900	-0.86639700
O	0.22201200	-0.52522300	-1.78611900
B	0.19255700	-1.88677300	-1.93178100
O	1.26838600	-2.43907600	-2.57813200
O	0.40343000	0.42187100	0.39276100
B	1.39877600	-0.64443100	1.00994200
C	2.69943300	-0.77246600	0.08978300
H	2.69717300	-1.54725300	-0.67686800
C	-1.03573800	-2.72782700	-1.47518200
H	-0.95015300	-3.81517800	-1.45011200
C	-2.20170100	-2.16225200	-1.10696100
H	-2.28361300	-1.07644200	-1.15343900
C	3.77941400	0.02379900	0.16492500
H	3.81569800	0.78134100	0.95029000
C	4.96776700	-0.01157400	-0.70346900
C	6.11610200	0.71093900	-0.33439800
C	5.00385800	-0.73693100	-1.91029700
C	7.26465900	0.69746800	-1.12533000
H	6.10235900	1.28409300	0.58938100
C	6.15015800	-0.75115000	-2.70000200
H	4.12068600	-1.27769100	-2.23654200
C	7.28850200	-0.03665000	-2.31222800

H	8.14021300	1.26110800	-0.81550000
H	6.15480800	-1.31537700	-3.62845100
H	8.17961900	-0.04751600	-2.93277600
C	-3.41547500	-2.81969300	-0.61109000
C	-4.51419600	-2.01811000	-0.25317500
C	-3.52544700	-4.21357600	-0.44994300
C	-5.68422900	-2.58519200	0.24863600
H	-4.43858000	-0.94014800	-0.36974600
C	-4.69335500	-4.77976600	0.05126000
H	-2.69194500	-4.85520900	-0.71636700
C	-5.77795000	-3.96922700	0.40306600
H	-6.52072300	-1.94799500	0.51980200
H	-4.76048000	-5.85710100	0.17078100
H	-6.68702100	-4.41532800	0.79492100
O	0.74186900	-1.90645700	1.13608500
C	-0.46074300	-1.96335500	1.88503900
H	-1.27988700	-1.48602200	1.32868700
H	-0.35468400	-1.40934100	2.82932400
C	-0.80787000	-3.41434500	2.16601000
H	-0.01696400	-3.89426500	2.75262100
H	-1.74787900	-3.48872700	2.72325300
H	-0.92075000	-3.96820000	1.22925600
H	1.16655300	-3.39330700	-2.69707700
O	1.68576600	0.01583400	2.33222400
C	2.44327900	-0.70508400	3.29960400
H	3.27238300	-1.21511600	2.79583600
H	1.81317800	-1.47599000	3.76263500
C	2.96748400	0.25658100	4.35017200
H	2.14191600	0.76985300	4.85604600
H	3.54497300	-0.28374200	5.10721300
H	3.61759200	1.01184700	3.89587300
H	0.34516000	1.46934400	1.15989800
O	0.55565100	2.11947200	2.05800900
H	1.10051700	1.31570500	2.47384500
C	1.47806100	3.19666900	1.70462100
H	1.89255600	3.56575200	2.64545300
H	2.28949800	2.77616200	1.10196400
C	0.73009400	4.27791900	0.95737600
H	0.34321000	3.90308600	0.00603700
H	-0.10675000	4.65604800	1.55194600
H	1.41029300	5.10930900	0.74796100

TS2-E-4

C	-3.23382700	-0.71888700	0.84136600
H	-2.64388500	-1.27646400	1.56891800
C	-2.67214300	0.37597200	0.28905700
H	-3.24398600	0.94547500	-0.44452000
C	-4.56383500	-1.28046400	0.58368400
C	-4.94792600	-2.44594700	1.27028000
C	-5.47626300	-0.70927300	-0.32386700
C	-6.19879700	-3.02403800	1.06175600
H	-4.25129000	-2.89676800	1.97173200
C	-6.72489500	-1.28593600	-0.53207200
H	-5.20522500	0.18934100	-0.86891800
C	-7.09238600	-2.44543100	0.15932700
H	-6.47511500	-3.92435400	1.60230900
H	-7.41658900	-0.83264500	-1.23595800
H	-8.06795200	-2.89244300	-0.00628000
B	-1.22746200	0.82845500	0.64704400
O	-0.69665500	1.90794600	-0.01836200
B	0.71187300	2.57640700	0.19247700
O	0.84902600	3.09651300	1.51519300
O	-0.52632700	0.18770000	1.61952100
B	0.58461100	-0.56756300	1.89167400
C	1.07391500	-1.69610800	0.93925700
H	2.00178200	-2.20296600	1.20067400
C	1.83527300	1.50539400	-0.17178900
H	1.70230900	0.94747700	-1.10239500
C	2.89769800	1.20237700	0.59219400
H	3.01578900	1.73334400	1.53647900
C	0.43140400	-2.05548100	-0.18810200
H	-0.50596300	-1.55622200	-0.43431800
C	0.84691500	-3.06281000	-1.17133600
C	-0.00437400	-3.34868900	-2.25327000
C	2.07421900	-3.74797500	-1.09033700
C	0.35032700	-4.28913800	-3.21926400
H	-0.95299400	-2.82368200	-2.32918800
C	2.42937500	-4.68495800	-2.05560900
H	2.75707000	-3.53527100	-0.27547000
C	1.56977700	-4.96159500	-3.12415300
H	-0.32338600	-4.49543100	-4.04573100
H	3.38230300	-5.20037800	-1.97806800
H	1.85138200	-5.69270500	-3.87599700
C	3.90352200	0.15718000	0.34522000
C	4.78104700	-0.20423700	1.38247200
C	4.00260400	-0.53886800	-0.87411800
C	5.71241000	-1.22903600	1.21747500
H	4.71417800	0.31922400	2.33260000
C	4.93462600	-1.55893800	-1.04249800
H	3.34078400	-0.28161900	-1.69459900

C	5.79335500	-1.91337300	0.00334000
H	6.37356500	-1.49426700	2.03762200
H	4.98796300	-2.08506100	-1.99115400
H	6.51767500	-2.71148200	-0.12950400
O	1.25100200	-0.40493700	3.06993000
C	0.90746500	0.66914900	3.96126000
H	0.68146600	1.56679000	3.37814800
H	1.80559600	0.86388700	4.55723200
C	-0.25351400	0.28682300	4.86512000
H	-0.02659400	-0.62697400	5.42431000
H	-0.45169100	1.08898600	5.58477000
H	-1.16262300	0.11883000	4.28003500
H	0.20707100	3.80544600	1.65341700
O	0.60894400	3.68895500	-0.83621400
C	1.79033700	4.43898100	-1.11272300
H	2.24710600	4.73367000	-0.16047500
H	2.51646700	3.80902200	-1.64399900
C	1.43567100	5.66301600	-1.93660300
H	0.72239100	6.29778900	-1.40051300
H	2.33370300	6.25395100	-2.14413900
H	0.99228600	5.37464400	-2.89607000
H	-0.49324200	3.53516300	-1.71674900
O	-1.44031600	3.10216200	-1.94343300
H	-1.28633500	2.41056000	-1.07555900
C	-2.49117600	4.06199400	-1.61911200
H	-2.26619900	4.50874100	-0.64490200
H	-3.41469800	3.48478100	-1.53556900
C	-2.58324300	5.10732200	-2.70831700
H	-3.39563400	5.80375300	-2.47825400
H	-1.65415400	5.68048500	-2.78420200
H	-2.78920900	4.64201300	-3.67660000

TS2-E-5

C	-0.79434000	2.72298600	0.22547100
H	-0.63269600	2.35326900	1.23826800
C	-0.43719700	1.91441000	-0.79276600
H	-0.58763500	2.25303000	-1.81840100
C	-1.38961900	4.06249700	0.15348000
C	-1.67966000	4.74009600	1.35132100
C	-1.68972500	4.70490400	-1.06308600
C	-2.24693300	6.01330400	1.33968000
H	-1.45367700	4.25524000	2.29736100
C	-2.25563000	5.97579300	-1.07533900
H	-1.47921800	4.20478100	-2.00292900
C	-2.53710100	6.63673000	0.12518600
H	-2.46184900	6.51802600	2.27697400
H	-2.47986800	6.45552100	-2.02360800
H	-2.97898800	7.62848400	0.11117200
B	0.13467600	0.48138000	-0.55487400
O	0.36676600	0.06012500	0.71019900
B	1.14504600	-1.06836500	1.16388700
O	0.53635700	-2.33216900	0.50187600
O	0.38392800	-0.30507500	-1.66264000
B	0.00977000	-1.57546400	-2.02119700
C	-1.47195000	-2.04936200	-1.88293600
H	-1.69771500	-3.10538100	-2.03614600
C	2.70907300	-0.99733600	0.83721300
H	3.30929500	-1.90707400	0.93088500
C	3.33676600	0.12228400	0.43886100
H	2.74730100	1.03633800	0.35559600
C	-2.47303500	-1.20836200	-1.56155400
H	-2.23160200	-0.15574000	-1.41090600
C	-3.88690600	-1.52506600	-1.32657000
C	-4.71882500	-0.52537800	-0.79094100
C	-4.44192600	-2.79508600	-1.56958200
C	-6.05580600	-0.78557600	-0.49418800
H	-4.30114800	0.45981000	-0.60054000
C	-5.77786500	-3.05373300	-1.27699200
H	-3.82382900	-3.58021400	-1.99335900
C	-6.59053500	-2.05222700	-0.73530700
H	-6.67927000	-0.00050600	-0.07667800
H	-6.18997600	-4.03943100	-1.47236800
H	-7.63180100	-2.25882000	-0.50725500
C	4.75228000	0.28719200	0.07187900
C	5.22448600	1.57016400	-0.25680100
C	5.66310100	-0.78476700	0.01778400
C	6.55428300	1.78020900	-0.61961900
H	4.53324300	2.40836200	-0.22528600
C	6.99062300	-0.57690200	-0.34518500
H	5.32663000	-1.78887500	0.25650400
C	7.44495100	0.70669500	-0.66522400
H	6.89387100	2.78159800	-0.86812600
H	7.67570600	-1.41921400	-0.38185800
H	8.48100300	0.86517500	-0.94911500
O	0.89284800	-2.42203400	-2.63071900
C	2.27753100	-2.06465000	-2.75302200
H	2.83002100	-3.00724300	-2.83219100
H	2.60865700	-1.54964300	-1.84647200
C	2.52596300	-1.19937600	-3.97723000
H	2.00180300	-0.24374100	-3.88585800

H	3.59717400	-0.99511700	-4.08217100
H	2.17967400	-1.70218000	-4.88625300
H	1.17095500	-3.05770400	0.41632800
O	0.82915200	-1.16767900	2.63884000
C	1.73077800	-1.88502200	3.48859400
H	1.86653800	-2.91412200	3.12271200
H	2.71019400	-1.39456100	3.45766200
C	1.18300500	-1.89683300	4.90378600
H	0.21963600	-2.41640600	4.95020700
H	1.87820300	-2.41073200	5.57556400
H	1.04078400	-0.87449900	5.26810700
H	-0.52806300	-2.58735800	1.26406900
O	-1.18639500	-2.45963000	2.15593100
H	-0.45792100	-1.82575800	2.60459800
C	-2.40519700	-1.71364100	1.83891900
H	-2.12719900	-0.83495100	1.25254400
H	-3.00921200	-2.37711900	1.21982100
C	-3.11462900	-1.33317500	3.11860400
H	-2.49508200	-0.66867400	3.72968000
H	-4.04081800	-0.80495700	2.87119600
H	-3.36760900	-2.22030900	3.70666900

Int2-E

C	1.55926600	1.25506200	0.11477300
H	1.14076300	1.29978600	-0.89082600
C	0.72559300	1.39282900	1.16454400
H	1.13879700	1.35791000	2.17265800
C	3.01075300	1.04094800	0.13745300
C	3.68993000	0.88974800	-1.08445800
C	3.75868200	0.97618200	1.32832600
C	5.06715500	0.67879900	-1.12062600
H	3.12348000	0.93754400	-2.01067400
C	5.13341900	0.76669000	1.29255100
H	3.26036700	1.09182200	2.28528000
C	5.79453700	0.61665000	0.06877900
H	5.57143900	0.56321800	-2.07536400
H	5.69460200	0.72000600	2.22114500
H	6.86765800	0.45275300	0.04549900
B	-0.80602300	1.61158800	1.02464300
O	-1.51237000	1.87475600	2.16187600
O	-1.45584200	1.59372800	-0.18437200
B	-1.42566200	0.86697400	-1.34881900
C	-1.24289100	-0.67590500	-1.37463200
H	-1.15470300	-1.15544600	-2.34955900
C	-1.20007600	-1.44139400	-0.26656700
H	-1.30208700	-0.95601000	0.70433700
C	-1.02770400	-2.89587200	-0.17994300
C	-0.99696200	-3.49705300	1.09086300
C	-0.88694000	-3.72138500	-1.31147800
C	-0.83054700	-4.87360100	1.23178900
H	-1.10305800	-2.87039500	1.97228300
C	-0.72155000	-5.09549600	-1.17113000
H	-0.90702100	-3.28456100	-2.30461900
C	-0.69237800	-5.67861200	0.10024000
H	-0.80841100	-5.31660200	2.22297500
H	-0.61443500	-5.71704300	-2.05523600
H	-0.56226500	-6.75150300	0.20502100
O	-1.60249000	1.49975900	-2.54518400
C	-1.78645200	2.92159500	-2.61356100
H	-1.43044100	3.22859400	-3.60201600
H	-1.16535700	3.41570500	-1.85904300
C	-3.24850100	3.29287200	-2.43130100
H	-3.59791600	3.01216600	-1.43321400
H	-3.38134400	4.37343100	-2.55156600
H	-3.87148400	2.78425800	-3.17396400
C	-2.93492200	2.06496200	2.11984600
H	-3.15504900	3.02776800	1.64270900
H	-3.39597100	1.28101400	1.50863900
C	-3.47011200	2.03218600	3.53772800
H	-3.01172700	2.81932700	4.14479200
H	-4.55411500	2.18561500	3.53510300
H	-3.25949700	1.06666000	4.00877800

Int3

O	3.95719200	-1.08557000	0.14283100
B	3.04910700	-0.06968500	-0.00574800
C	1.53882600	-0.45267700	-0.03076400
H	1.29402900	-1.51478800	-0.05398900
C	0.52956500	0.43931400	0.00229200
H	0.77021600	1.50344100	0.04175400
C	-0.91424200	0.17215300	0.00104200
C	-1.80387700	1.25921000	0.06447700
C	-1.45272000	-1.12695000	-0.06099700
C	-3.18349100	1.06083600	0.06704700
H	-1.40202800	2.26783200	0.11328700
C	-2.82967400	-1.32518000	-0.05907100
H	-0.78962800	-1.98444100	-0.11144200
C	-3.70189300	-0.23329400	0.00517800

H	-3.85215600	1.91500100	0.11721500
H	-3.22728700	-2.33457100	-0.10802400
H	-4.77587300	-0.39292200	0.00668000
O	3.53424900	1.21156700	-0.11587100
H	2.84440200	1.87070600	-0.27103100
H	4.86629500	-0.75027100	0.14339200

TS3-E

C	-1.17421100	2.21549500	-0.04782600
H	-0.95075300	2.85236700	-0.90357000
C	-0.15644000	1.66114500	0.63996400
H	-0.39219600	1.00499700	1.47478500
C	-2.60914100	1.99108300	0.17174800
C	-3.53664400	2.66233200	-0.64385000
C	-3.09842600	1.08058100	1.12681900
C	-4.90562900	2.43435600	-0.51454300
H	-3.17143600	3.36144100	-1.39167000
C	-4.46417000	0.85088700	1.25454800
H	-2.40469400	0.53059900	1.75275000
C	-5.37463700	1.52447900	0.43410200
H	-5.60452500	2.96059300	-1.15806700
H	-4.82111000	0.13095500	1.98472300
H	-6.43944000	1.33501500	0.53070200
B	1.33153200	1.69305600	0.18677000
O	2.01953100	2.75883100	-0.31845700
O	2.08034400	0.53674500	0.24347100
B	1.58425200	-0.90825100	0.54508000
C	0.22581400	-1.19610900	-0.24913400
H	0.18198100	-0.88698700	-1.29718700
C	-0.86978500	-1.76467300	0.28322800
H	-0.81627900	-2.08981100	1.32254900
C	-2.18237100	-1.95938400	-0.35270400
C	-3.15132900	-2.74763700	0.29260900
C	-2.54201000	-1.33230100	-1.56047300
C	-4.42548900	-2.91491800	-0.24860900
H	-2.89574500	-3.22841800	1.23351800
C	-3.81449000	-1.49438500	-2.09993100
H	-1.82622100	-0.68950200	-2.06242400
C	-4.76440500	-2.28773300	-1.44861400
H	-5.15583900	-3.52950500	0.27020100
H	-4.07330100	-0.98919300	-3.02622200
H	-5.75888100	-2.40670700	-1.86822000
O	1.41034600	-1.10885400	1.95001800
C	2.51499400	-0.90124000	2.80923900
H	2.94095700	0.10246300	2.65643400
H	3.31930200	-1.62122000	2.59137700
C	2.05748300	-1.05509900	4.24981700
H	1.64526300	-2.05616900	4.41652600
H	2.89195000	-0.90536700	4.94353400
H	1.27676700	-0.32362500	4.48466400
C	1.52617800	4.10251200	-0.35781800
H	1.11087900	4.29086200	-1.35533600
H	0.71970100	4.22988900	0.37104800
C	2.67106300	5.05573600	-0.07094900
H	3.47902400	4.92236200	-0.79742400
H	2.32169700	6.09180200	-0.12931600
H	3.07555900	4.88284100	0.93150300
O	2.75960500	-1.69449500	-0.01432200
H	3.84819100	-0.88384500	-0.38443000
O	4.27192200	0.07928300	-0.57325600
H	3.32376500	0.53014300	-0.18442500
C	2.75170400	-3.11553400	0.10876900
H	3.75264200	-3.43052800	0.42822500
H	2.05047300	-3.39632200	0.90184300
C	2.37780900	-3.78421500	-1.20486600
H	2.41537800	-4.87465400	-1.10252000
H	1.36554000	-3.50004700	-1.50622800
H	3.07120600	-3.49101500	-2.00061300
C	4.33230300	0.29553500	-2.01480400
H	3.38455000	-0.03126000	-2.45587600
H	4.43004800	1.37401600	-2.15525400
C	5.50854400	-0.45788800	-2.59475900
H	5.40146400	-1.53587000	-2.43604800
H	5.56491200	-0.27653600	-3.67262300
H	6.44597000	-0.12802500	-2.13760300

TS4-E

O	1.80580800	-2.59015500	-1.25757100
B	1.45501500	-1.24993500	-0.90599200
C	-0.13830900	-1.05136300	-0.92330500
H	-0.68992700	-1.50204400	-1.75402000
C	-0.84366100	-0.36166000	-0.00939300
H	-0.29879700	0.08073000	0.82588300
C	-2.29450600	-0.11215700	0.02550800
C	-2.82727900	0.65968100	1.07357800
C	-3.18621900	-0.60753500	-0.94488000
C	-4.19286500	0.93022000	1.15309000
H	-2.15388300	1.04944000	1.83296100

C	-4.54975900	-0.33864800	-0.86708600
H	-2.80748500	-1.20968100	-1.76481000
C	-5.06231700	0.43194800	0.18169900
H	-4.57756200	1.52930600	1.97357200
H	-5.21860100	-0.73253800	-1.62720200
H	-6.12667900	0.63919600	0.23929000
O	2.05816200	-0.82939600	0.43374500
C	1.97877700	-1.70241800	1.55734200
H	2.68508200	-2.53198700	1.42774400
H	0.97109900	-2.13160800	1.60040700
C	2.28545600	-0.92899600	2.82673000
H	3.28955100	-0.49250200	2.78528800
H	1.56258900	-0.11968900	2.97534300
H	2.23983600	-1.59310800	3.69580100
O	2.13777800	-0.22164800	-1.84154900
H	1.51573700	0.05935000	-2.52668500
H	2.61099100	0.37273700	0.37976100
O	2.91994400	1.29608900	-0.15117200
H	2.62841800	0.79345300	-1.08643500
C	1.97814600	2.36810900	0.13798400
H	2.07119700	2.58383000	1.20526000
H	0.96221800	2.00890200	-0.05773700
C	2.31122700	3.57892400	-0.70641600
H	1.62016100	4.39416300	-0.47011700
H	3.33189500	3.92116400	-0.51181900
H	2.21608100	3.35117700	-1.77327800
H	2.76730500	-2.65637500	-1.33597600

Int4-E

O	2.72571400	-0.87432900	0.00023000
B	1.66550300	-0.00777900	0.00010200
C	0.17171100	-0.45261700	0.00020400
H	-0.07354500	-1.51395200	0.00052300
C	-0.84103900	0.43782400	-0.00010600
H	-0.58561200	1.49725300	-0.00034400
C	-2.28274300	0.16236200	-0.00009200
C	-3.17851000	1.24656600	0.00054900
C	-2.81587600	-1.14081800	-0.00071300
C	-4.55723800	1.04162900	0.00065200
H	-2.78088700	2.25792100	0.00099500
C	-4.19178100	-1.34598600	-0.00062300
H	-2.14874300	-1.99684900	-0.00131500
C	-5.06950600	-0.25650300	0.00007000
H	-5.23031200	1.89386500	0.00116900
H	-4.58456100	-2.35852700	-0.00112500
H	-6.14276600	-0.42117300	0.00012700
O	1.93896100	1.33437100	-0.00011500
C	3.28971800	1.81080900	-0.00002700
H	3.81626900	1.42819700	-0.88280600
H	3.81600900	1.42864600	0.88309900
C	2.55831000	-2.29653500	0.00004900
H	1.98882700	-2.60368000	0.88583600
H	1.98923200	-2.60348300	-0.88606900
C	3.92974200	-2.94341400	0.00030400
H	3.83384400	-4.03397900	0.00010300
H	4.49740100	-2.64591500	-0.88713900
H	4.49694900	-2.64620200	0.88813200
C	3.26651700	3.32695100	-0.00043600
H	2.74982700	3.70618800	-0.88805700
H	2.74962300	3.70665900	0.88686800
H	4.28769100	3.72198700	-0.00041400

TS5-E

O	1.47584500	-0.52260600	1.07641300
B	0.81433900	0.12250200	-0.03326900
C	-0.75977800	0.38119200	0.12762400
H	-1.06566600	1.19291000	0.79319600
C	-1.73819300	-0.33870900	-0.44936500
H	-1.45715500	-1.17277500	-1.09560000
C	-3.19454900	-0.16005900	-0.31786500
C	-4.05424100	-1.08210500	-0.94111500
C	-3.77809300	0.89730900	0.40562600
C	-5.44002500	-0.96145200	-0.84352700
H	-3.62213700	-1.90429900	-1.50597400
C	-5.16115600	1.01953000	0.50387000
H	-3.14284800	1.63171800	0.89081700
C	-6.00142800	0.09082500	-0.11888400
H	-6.08073000	-1.68920700	-1.33336000
H	-5.58833300	1.84449500	1.06707600
H	-7.07995600	0.18984500	-0.04076100
O	1.56796700	1.42218300	-0.27344800
C	1.68976900	2.34191500	0.81514300
H	2.02209700	1.80701200	1.71197100
H	0.70119200	2.76814400	1.02847200
C	0.94984800	-1.74537000	1.55426200
H	-0.05905700	-1.59583200	1.96956400
H	0.84650600	-2.47034600	0.73222400
C	1.87327700	-2.30424800	2.62394200
H	1.47194900	-3.23697700	3.03508500
H	2.86694100	-2.51088800	2.21146300

H	1.98790000	-1.58722700	3.44411000
C	2.66658300	3.44310300	0.44450700
H	3.66876800	3.03635800	0.26801600
H	2.34115500	3.96204300	-0.46295900
H	2.73701800	4.17587900	1.25498100
O	1.11044700	-0.70781600	-1.28811200
H	0.51448000	-0.45433800	-2.00886200
H	2.73050100	0.98674000	-0.92324500
O	3.29558500	0.26921900	-1.50269600
H	2.37822200	-0.35802300	-1.56390300
C	4.29478900	-0.37349200	-0.64939100
H	3.83981500	-0.54726000	0.32955900
H	5.11527000	0.34109200	-0.54882000
C	4.75156700	-1.66359200	-1.29407300
H	5.52422300	-2.12847600	-0.67379200
H	3.91974600	-2.36947700	-1.38609700
H	5.16942400	-1.48053900	-2.28859800

Int5-E

O	2.72556700	-0.87436300	0.00029300
B	1.66541900	-0.00771000	0.00009300
C	0.17162000	-0.45246700	0.00016800
H	-0.07359300	-1.51381300	0.00054300
C	-0.84115300	0.43794300	-0.00019500
H	-0.58575300	1.49738400	-0.00054400
C	-2.28284800	0.16244700	-0.00014000
C	-3.17864900	1.24662300	0.00033600
C	-2.81593800	-1.14075200	-0.00054900
C	-4.55737100	1.04163800	0.00048200
H	-2.78105800	2.25799100	0.00061700
C	-4.19183600	-1.34596600	-0.00041900
H	-2.14877100	-1.99675500	-0.00101000
C	-5.06959500	-0.25651000	0.00010700
H	-5.23047200	1.89385300	0.00087000
H	-4.58458600	-2.35851900	-0.00075400
H	-6.14284900	-0.42121600	0.00019700
O	1.93907500	1.33437700	-0.00018400
C	3.28988500	1.81055700	-0.00021000
H	3.81622300	1.42817400	-0.88322600
H	3.81632200	1.42801300	0.88267400
C	2.55830800	-2.29650900	0.00036000
H	1.98931400	-2.60365500	0.88647600
H	1.98879000	-2.60369400	-0.88540300
C	3.92979300	-2.94332100	-0.00002800
H	3.83395700	-4.03388800	0.00003300
H	4.49694700	-2.64594600	-0.88783800
H	4.49747300	-2.64589500	0.88742900
C	3.26696700	3.32670200	-0.00010100
H	2.75010100	3.70630100	-0.88746700
H	2.75039700	3.70623300	0.88746600
H	4.28821900	3.72154900	-0.00026400

TS6-E

C	3.17070400	1.00522700	0.34071100
C	3.58874200	2.29328600	0.56383500
C	1.79043000	0.65698400	0.38305800
C	2.64289400	3.31688300	0.82504800
C	0.84300500	1.63261700	0.65322100
C	1.25196500	2.98649100	0.86976200
O	1.39151600	-0.62990000	0.14711500
B	0.65655700	-1.08939100	-1.22417500
C	-0.87524800	-1.45699300	-0.93719000
H	-1.60252500	-1.10625400	-1.67221200
C	-1.33714500	-2.17973100	0.09779500
H	-0.63257300	-2.52034000	0.85674900
O	0.72627200	-0.00346700	-2.16896500
O	1.50086900	-2.25545200	-1.55892700
C	1.05769800	-3.08216700	-2.63700800
H	0.10609400	-3.56261800	-2.37342500
H	0.87501600	-2.45715700	-3.52053300
C	1.93785600	0.32946900	-2.85369400
H	2.58357800	-0.55205600	-2.89771200
H	2.47281300	1.10428500	-2.29164500
H	4.64239600	2.54701400	0.52870000
C	-0.59933000	1.27856100	0.78025600
C	-1.09584800	0.76226900	2.01393800
C	-1.45438100	1.47717300	-0.29815500
C	-2.49602100	0.49916400	2.14845700
C	-2.84725300	1.23616100	-0.12570000
C	-3.35960100	0.76753000	1.05555300
H	-4.41849100	0.55972800	1.15922200
O	-1.00040800	1.88648000	-1.50440600
H	-0.25913200	1.27784500	-1.78086500
C	3.05200500	4.65984300	1.04326000
H	4.11234800	4.89314700	1.00423000
C	2.12238300	5.64226200	1.29709600
H	2.44128400	6.66705600	1.46151300
C	0.74471100	5.31806300	1.34564500
H	0.01743800	6.09876600	1.54740300
C	0.31910500	4.02464000	1.13848100

H	-0.73686300	3.78384800	1.17796800
C	-0.24838800	0.47756900	3.11962600
H	0.81391600	0.67807700	3.03399900
C	-0.76014500	-0.05154500	4.28442100
H	-0.09513200	-0.26839200	5.11540900
C	-2.14512300	-0.31869100	4.40999200
H	-2.53277600	-0.74056400	5.33233600
C	-2.99437300	-0.04387500	3.36173100
H	-4.05802700	-0.24971400	3.43873400
C	-2.73558800	-2.53419800	0.38318900
C	-3.05213500	-3.11746000	1.62269200
C	-3.78884700	-2.28534200	-0.51677100
C	-4.36997000	-3.42276600	1.96290800
H	-2.25126600	-3.30991500	2.33181700
C	-5.10473800	-2.58771100	-0.17893400
H	-3.57569000	-1.83890800	-1.48220400
C	-5.40497900	-3.15542200	1.06461600
H	-4.58868300	-3.86459800	2.93117100
H	-5.90178900	-2.37844100	-0.88703900
H	-6.43293300	-3.38910600	1.32606300
C	2.12377100	-4.12050600	-2.93370600
H	2.29653600	-4.76255100	-2.06239000
H	1.81347900	-4.75832300	-3.76784400
H	3.07081900	-3.63940600	-3.19886600
C	1.60001000	0.82864800	-4.24757600
H	1.08038400	0.05542100	-4.82287200
H	0.95332600	1.71152500	-4.19983500
H	2.51415700	1.10480800	-4.78430000
I	4.64164000	-0.47496700	-0.12309200
I	-4.15029200	1.53744700	-1.79447600
H	1.82700400	-2.96479700	-0.14827700
O	1.87518800	-2.81355900	0.85611500
H	1.78558300	-1.65645700	0.73766900
C	3.01614900	-3.44183700	1.50878800
H	3.88095900	-3.37808400	0.84492400
H	3.21529100	-2.84248100	2.39924500
C	2.66657700	-4.87300800	1.85609800
H	3.51400500	-5.34176400	2.36667800
H	1.79624700	-4.91204200	2.51759000
H	2.44812300	-5.45436700	0.95445300

Int6-E

C	-3.15184500	1.08966700	-0.70342700
C	-4.21001300	0.33518200	-1.14543200
C	-1.92073800	0.48711600	-0.33940300
C	-4.08404600	-1.07381400	-1.25299200
C	-1.76572600	-0.89120800	-0.42404600
C	-2.84495300	-1.69799400	-0.89614300
O	-0.87586800	1.27419300	0.02935700
B	-0.26618600	1.20885900	1.28021100
C	1.21185300	1.66249900	1.36616400
H	1.68659700	1.79849800	2.33611200
C	1.94473600	1.85955000	0.25019000
H	1.45171200	1.71329400	-0.70984800
O	-1.03136800	0.76646400	2.31096800
C	-0.51579000	0.60857900	3.63877000
H	0.52006700	0.25473900	3.60209300
H	-1.11437100	-0.17835300	4.10525500
H	-5.15027700	0.80288600	-1.41599100
C	-0.46605600	-1.49345500	-0.01059700
C	-0.35073700	-2.21418500	1.21662000
C	0.65441200	-1.30146700	-0.81351500
C	0.92209400	-2.73771200	1.60972000
C	1.91100700	-1.82889000	-0.40223800
C	2.04495000	-2.52556500	0.77133300
H	3.00959000	-2.91804200	1.07337900
O	0.49215600	-0.60270000	-1.96140900
H	1.34444400	-0.51767400	-2.42187700
C	-5.16247600	-1.87573000	-1.71537700
H	-6.09631000	-1.38708700	-1.97930200
C	-5.02550300	-3.24023700	-1.82827000
H	-5.85446900	-3.84558200	-2.18225800
C	-3.79763900	-3.85889100	-1.48707100
H	-3.69400700	-4.93535500	-1.58659700
C	-2.73534600	-3.10932800	-1.03347100
H	-1.79804500	-3.59029100	-0.77800500
C	-1.45823300	-2.41870900	2.08551200
H	-2.42282500	-2.00914000	1.81251500
C	-1.31060300	-3.11227700	3.26577800
H	-2.16941100	-3.25692400	3.91462400
C	-0.05003000	-3.63430300	3.64852300
H	0.04874100	-4.17725000	4.58348700
C	1.04365500	-3.44681200	2.83552000
H	2.01872300	-3.83565000	3.11552800
C	3.35724100	2.24092900	0.15688100
C	3.90917800	2.47472400	-1.11537600
C	4.19364500	2.37040300	1.28168700
C	5.24813900	2.83179600	-1.26364800
H	3.27465600	2.37202600	-1.99132600
C	5.53132800	2.72448300	1.13428400

H	3.79581500	2.18527000	2.27445200
C	6.06481800	2.95734100	-0.13818100
H	5.65430000	3.00888000	-2.25520100
H	6.16366600	2.81708800	2.01252500
H	7.10963200	3.23204700	-0.24896400
C	-0.62604300	1.90513200	4.42236400
H	-0.01179500	2.69113000	3.97110700
H	-1.66412700	2.25173700	4.44586900
H	-0.28865500	1.75306800	5.45329700
I	-3.34795500	3.21217200	-0.54120000
I	3.63984700	-1.51968600	-1.63329400

TS7-E

C	-2.97010100	-0.84359800	-0.90850700
C	-4.14044600	-0.14308500	-0.76243000
C	-1.70083500	-0.23020600	-0.70816700
C	-4.11255700	1.22058700	-0.37137900
C	-1.64588000	1.13595600	-0.42187000
C	-2.85105900	1.87262800	-0.18685400
O	-0.56809300	-0.94757000	-0.82231900
B	0.21438700	-1.25935300	0.38462100
C	1.68121600	-1.71741800	0.00715400
H	2.29868200	-2.13124100	0.80604800
C	2.24063300	-1.53497600	-1.20073800
H	1.61788600	-1.11954400	-1.99294300
O	-0.51388000	-2.08079900	1.34625600
C	-0.40974400	-3.50207400	1.27479800
H	0.60144400	-3.81228100	1.56949100
H	-0.57282300	-3.83361400	0.24318700
H	-5.09923600	-0.62366800	-0.92395100
C	-0.30590100	1.77756200	-0.34073500
C	0.06347800	2.89474100	-1.15953000
C	0.63229200	1.24038300	0.52685100
C	1.35881200	3.48356000	-0.98940500
C	1.90939600	1.82961500	0.68674400
C	2.26336400	2.93752900	-0.04167700
H	3.23760200	3.39692600	0.08251100
O	0.32188200	0.10385200	1.23438800
C	-5.31639500	1.94266400	-0.15153700
H	-6.26250700	1.43164600	-0.30725800
C	-5.28446700	3.25507100	0.26068600
H	-6.20871400	3.79875600	0.43108900
C	-4.03975500	3.89372100	0.47816700
H	-4.01795700	4.92246000	0.82549200
C	-2.85644500	3.22330100	0.26026700
H	-1.91301300	3.72367800	0.44173100
C	-0.78859000	3.42542300	-2.16658700
H	-1.75487500	2.96711100	-2.33601100
C	-0.39455900	4.49917100	-2.93342800
H	-1.05928300	4.88453000	-3.70072100
C	0.87136700	5.10192200	-2.73710300
H	1.16510200	5.95176500	-3.34566800
C	1.73082600	4.59900600	-1.78812000
H	2.71311400	5.03820600	-1.63916300
C	3.62921100	-1.81371500	-1.59748900
C	4.00304600	-1.64109400	-2.94229100
C	4.61785000	-2.23080800	-0.68563000
C	5.30927300	-1.88357400	-3.36659000
H	3.25330700	-1.31234600	-3.65752500
C	5.92220100	-2.47314100	-1.10766100
H	4.36332500	-2.35742100	0.36177500
C	6.27597200	-2.30238500	-2.45048300
H	5.57221900	-1.74406600	-4.41144700
H	6.66957800	-2.79290300	-0.38673100
H	7.29500100	-2.49041400	-2.77581100
C	-1.44443800	-4.12039200	2.19615300
H	-1.27785400	-3.80716900	3.23304600
H	-2.45370300	-3.81849100	1.89803600
H	-1.38546500	-5.21298800	2.15692200
I	-3.04777700	-2.89711700	-1.50041500
I	3.28128800	0.99915700	2.09746100
H	-1.41863500	-1.17195900	2.35721200
O	-1.46252100	-0.24617500	2.76011800
H	-0.60208000	0.12939300	2.04849000
C	-0.98400400	-0.32094400	4.14164000
H	-0.77522300	0.71021900	4.43224100
H	-0.04851200	-0.88883000	4.15250100
C	-2.04429300	-0.95106700	5.01702000
H	-2.25856300	-1.97721300	4.70115700
H	-1.69115100	-0.98052000	6.05263300
H	-2.97159400	-0.37203000	4.98197900

Int7

C	5.54524100	0.99731700	2.51897600
C	4.86488400	2.04279700	1.93924500
C	3.68820500	1.80952400	1.17729600
C	3.22472700	0.46923900	0.97987100
C	3.93697900	-0.58309500	1.61833100
C	5.06350300	-0.32470800	2.36755400
H	3.31249300	3.90498800	0.78571200

H	6.44012000	1.18362000	3.10476800
H	5.20544000	3.06664400	2.06448800
C	2.95098300	2.89383400	0.63529700
C	2.04939000	0.24414900	0.18378000
H	5.58445400	-1.14480700	2.85239200
C	1.30769800	1.33651500	-0.24098900
C	1.77483900	2.65991300	-0.02955200
C	1.60534600	-1.12977600	-0.17162900
C	2.49951300	-2.06297200	-0.80010000
C	0.30001000	-1.52180800	0.09216200
C	3.78474200	-1.69285400	-1.28367600
C	2.07691800	-3.41767100	-0.98772500
C	-0.10352800	-2.86919100	-0.09953600
C	4.61384700	-2.61988500	-1.87544900
C	2.96207500	-4.35613500	-1.58394200
C	0.76830900	-3.80251800	-0.59896100
C	4.20968500	-3.96906200	-2.01456400
H	5.58688000	-2.31009000	-2.24463100
H	2.62462300	-5.38170200	-1.70376900
H	0.45968700	-4.83320200	-0.73377600
H	4.87886200	-4.68965000	-2.47456500
O	-0.64678500	-0.63161500	0.53320900
O	0.09285300	1.18981100	-0.85949600
B	-0.92879700	0.50446900	-0.21658100
I	-2.09769000	-3.45543100	0.39545900
I	0.61186800	4.30400000	-0.74426600
C	-2.36470100	1.02715700	-0.33423200
H	-2.53195200	1.95441900	-0.87994000
C	-3.40860100	0.38518900	0.23620900
H	-3.20309400	-0.54306600	0.76626600
C	-4.81814700	0.77915300	0.22765800
C	-5.75786200	-0.07518300	0.83329900
C	-5.27795900	1.97253900	-0.36229400
C	-7.11409100	0.24531500	0.84619900
H	-5.41143400	-0.99687500	1.29305100
C	-6.63163100	2.29249500	-0.34814500
H	-4.57135900	2.65164800	-0.82857100
C	-7.55513900	1.43059100	0.25460700
H	-7.82509500	-0.42726200	1.31669500
H	-6.97165100	3.21660200	-0.80625200
H	-8.61092300	1.68465300	0.26332600
H	3.57771900	-1.59992500	1.52458700
H	4.10783400	-0.66328000	-1.19793800

TS1-B

B	-1.07635600	-0.81713500	0.68321800
C	-2.20031100	-1.52904100	1.55799800
O	0.13776500	-1.65137000	0.55375600
C	-2.09465700	-2.75226700	2.10531400
H	-3.11717500	-0.96529500	1.73226700
O	-1.63664800	-0.76536900	-0.82435400
O	-0.79703800	0.53157900	1.09355000
B	1.31240200	-1.27252400	1.14277300
C	-3.10285600	-3.46557800	2.90701900
H	-1.16748600	-3.30706200	1.95387900
C	-1.86692100	0.37549000	-1.54063400
B	0.32676300	0.81952100	1.80908600
C	2.58364200	-2.13525100	0.95277000
O	1.36275300	-0.10424000	1.88448200
C	-2.76177900	-4.69288100	3.50292500
C	-4.41224000	-2.98221400	3.09395400
C	-0.82613700	1.07118500	-2.14435700
C	-3.20570900	0.83049200	-1.69102400
C	0.46620700	2.17526400	2.55121900
C	3.76382900	-1.81479000	1.51968700
H	2.52896900	-3.00223300	0.29366600
C	-3.68527400	-5.40809000	4.26533400
H	-1.75633400	-5.08291500	3.36486000
C	-5.33508500	-3.69521200	3.85447500
H	-4.70944400	-2.04525100	2.63311800
C	-1.10266100	2.27138500	-2.87738800
C	0.56816900	0.54862900	-2.06630700
C	-3.49308900	1.98360100	-2.37731400
I	-4.80872700	-0.33313100	-0.88753900
C	-0.55299100	3.05498600	2.62002300
H	1.41948700	2.41947300	3.01956300
C	5.05744400	-2.47867500	1.32699700
H	3.79048200	-0.95166000	2.18291800
C	-4.97758500	-4.91177500	4.44586200
H	-3.39579700	-6.35253500	4.71765900
H	-6.34035000	-3.30395300	3.98451000
C	-0.07899900	3.02225000	-3.51729900
C	-2.45163500	2.73995500	-2.97298000
C	1.48856400	1.18439900	-1.24297500
C	0.97392700	-0.56508200	-2.86297100
H	-4.51665300	2.32634700	-2.48120600
C	-0.56651400	4.37626800	3.25899200
H	-1.49423600	2.77318500	2.14668700
C	6.15775900	-2.06280300	2.09785700
C	5.25533800	-3.50121400	0.37895700

H	-5.70056500	-5.46610100	5.03707600
C	-0.37754000	4.18067400	-4.19963900
H	0.94450400	2.66923400	-3.46582400
C	-2.72756400	3.93902600	-3.68324700
C	2.82544700	0.70974800	-1.16982200
O	1.16857800	2.26480000	-0.49121800
C	0.10558700	-1.19478700	-3.79509800
C	2.31162500	-1.06058300	-2.74100300
C	-1.76186900	5.11783100	3.25805000
C	0.56768100	4.94345800	3.87147500
C	7.41000500	-2.65511000	1.94114200
H	6.01982400	-1.26700700	2.82493800
C	6.50587400	-4.09107100	0.22058500
H	4.42723400	-3.82607700	-0.24319400
C	-1.71155200	4.64817300	-4.28181600
H	0.41767400	4.74146400	-4.68136100
H	-3.75647400	4.28172700	-3.74441000
C	3.22127200	-0.39566100	-1.87818600
I	4.22251600	1.72556600	0.08051400
C	0.52891100	-2.27600100	-4.53609100
H	-0.90216400	-0.81348500	-3.91870900
C	2.71176100	-2.19086100	-3.50179100
C	-1.82819500	6.37839800	3.85012800
H	-2.64420400	4.69291400	2.78648100
C	0.50216000	6.20178900	4.46222800
H	1.50537800	4.39705600	3.88013100
C	7.58860100	-3.67301600	1.00224400
H	8.24598600	-2.32092000	2.54870300
H	6.64106700	-4.87684300	-0.51718200
H	-1.92932800	5.56459500	-4.82170300
H	4.23576000	-0.76877800	-1.79505200
C	1.83807300	-2.79131800	-4.38052900
H	-0.14936100	-2.74003000	-5.24603600
H	3.72569100	-2.56304700	-3.38311000
C	-0.69547300	6.92558400	4.45549700
H	-2.76210800	6.93290800	3.83836100
H	1.38779100	6.62349500	4.92894100
H	8.56340800	-4.13444900	0.87482800
H	2.15277300	-3.65173200	-4.96318100
H	-0.74211300	7.90756300	4.91700100
C	-1.88561500	-3.95720500	-1.31874100
C	-2.69824100	-3.94982300	-2.59250100
H	-2.49273900	-3.73385200	-0.43868100
O	-0.85007800	-2.92263600	-1.44067200
H	-1.35861600	-4.90110400	-1.16820600

H	-2.06846400	-4.16237100	-3.46068700
H	-3.47530000	-4.71732600	-2.52693700
H	-3.18901800	-2.98215900	-2.73614000
H	-0.26683100	-2.86312300	-0.63412200
H	0.20333800	2.37820600	-0.48079500
H	-1.29046600	-1.86903400	-1.34053400

Int1-B

C	3.20746100	1.95994500	1.55123000
H	3.08955000	2.97766400	1.92308900
C	2.14705500	1.12960600	1.60379100
H	2.25553900	0.12758300	1.19217300
C	4.53418700	1.63997000	1.01401700
C	5.39167200	2.68386300	0.62463200
C	4.98161100	0.31400400	0.86547700
C	6.64248300	2.41407100	0.07171400
H	5.05934400	3.71142500	0.74387100
C	6.23609600	0.04458700	0.32472500
H	4.34577300	-0.50591100	1.18230800
C	7.06858500	1.09253400	-0.08208800
H	7.28589300	3.23333900	-0.23545100
H	6.56883700	-0.98443900	0.22463300
H	8.04590300	0.87983400	-0.50517700
B	0.80195900	1.51089500	2.27612500
O	0.63370400	2.75157400	2.82339800
B	-0.46533500	3.43838800	3.29948100
O	-0.23651500	4.13449000	4.45700700
O	-0.19322800	0.57486000	2.40244900
B	-0.62498400	-0.43908200	1.59222900
C	-0.19956700	-1.91094600	1.76277300
H	-0.72691700	-2.68994600	1.21633400
C	-1.84810200	3.47191100	2.59012900
H	-2.68951000	3.88415400	3.15110900
C	-2.08207800	3.02338800	1.33965600
H	-1.25349600	2.61172800	0.76744400
C	0.90994700	-2.23644900	2.45865800
H	1.45424800	-1.43790000	2.96199700
C	1.53866800	-3.55600500	2.55459300
C	2.86545000	-3.64049300	3.01222800
C	0.88628600	-4.74020100	2.16391400
C	3.53168500	-4.86423100	3.05365500
H	3.37576200	-2.73202700	3.32142200
C	1.54714900	-5.96368300	2.21734800
H	-0.14338100	-4.69931000	1.82359000
C	2.87466900	-6.03003500	2.65489500

H	4.56028200	-4.90872400	3.39916500
H	1.02866600	-6.86957500	1.91700300
H	3.38922600	-6.98569200	2.69023900
C	-3.36895800	2.97685500	0.63389700
C	-3.41987900	2.36234600	-0.63002300
C	-4.56565200	3.47626000	1.18183400
C	-4.62517500	2.23539800	-1.31825600
H	-2.50795100	1.96304200	-1.05828800
C	-5.76824200	3.35230800	0.49263900
H	-4.55712500	3.95942300	2.15376200
C	-5.80522600	2.72752800	-0.75909900
H	-4.64207900	1.74160700	-2.28558100
H	-6.68261200	3.74046000	0.93228100
H	-6.74738200	2.62660800	-1.28994600
H	-1.01458400	4.63811500	4.73271400
O	-1.47533400	-0.02217900	0.58624100
C	-1.97865800	-0.79823000	-0.41214300
C	-1.20811800	-1.08987000	-1.52805100
C	-3.32717900	-1.23643100	-0.32937700
C	-1.79685100	-1.79919000	-2.62212300
C	0.22465700	-0.67628500	-1.57430500
C	-3.90751700	-1.93155100	-1.36028200
I	-4.44578300	-0.82319200	1.44142700
C	-3.16186300	-2.22307100	-2.53199900
C	-1.07454800	-2.09582400	-3.81018800
C	1.26410900	-1.64580300	-1.42907000
C	0.54849700	0.66090200	-1.77425900
H	-4.93683600	-2.26616500	-1.29366000
C	-3.74729500	-2.92935700	-3.61714100
C	-1.67052500	-2.78018500	-4.84623600
H	-0.04412600	-1.77057800	-3.89387900
C	0.99498100	-3.02267300	-1.19362500
C	2.63130000	-1.23038200	-1.51100700
C	1.91452600	1.05288800	-1.85574900
O	-0.47172500	1.54360100	-1.90545200
C	-3.01793700	-3.20520800	-4.75100900
H	-4.78325700	-3.24522000	-3.53184500
H	-1.10295800	-2.99522400	-5.74673500
C	2.02102400	-3.92656400	-1.03462400
H	-0.03423000	-3.35397600	-1.12850400
C	3.66894700	-2.18716500	-1.34374800
C	2.92791500	0.13721200	-1.73869300
I	2.40087600	3.11905800	-2.15100700
H	-3.47307400	-3.74509400	-5.57575800
C	3.37244500	-3.50896500	-1.10398100
H	1.79317400	-4.96918300	-0.83931200
H	4.69837400	-1.84765600	-1.40136200
H	3.96328100	0.44792100	-1.80045200
H	4.16853200	-4.23388000	-0.96539300
H	-0.12444300	2.44626100	-2.01074600

TS2-B

C	-0.54424800	-2.63729800	-1.22344800
C	-1.03956000	-3.91543600	-1.28872300
C	-1.38205700	-1.52726800	-0.91665600
C	-2.40877300	-4.16563200	-1.01136100
C	-2.75080200	-1.73518400	-0.73306800
C	-3.27857500	-3.06694700	-0.71488000
O	-0.86261800	-0.28850700	-0.83069500
B	-0.76565300	0.36632100	0.47540600
C	-0.50519100	1.91979400	0.31273400
H	-0.21988600	2.47207700	1.20924600
C	-0.55750500	2.58286300	-0.85454300
H	-0.81121100	2.02050400	-1.75301300
O	0.14956400	-0.33687500	1.38631700
H	-0.39043400	-4.75124000	-1.52595400
C	-3.61791700	-0.54594400	-0.51426100
C	-4.75027500	-0.25679000	-1.34495500
C	-3.29179100	0.33457100	0.50582300
C	-5.56849400	0.87795800	-1.03468000
C	-4.10250600	1.45720900	0.79955200
C	-5.22890100	1.71589000	0.05958000
H	-5.85860600	2.56933800	0.28483200
O	-2.14364900	0.12447900	1.23257000
C	-2.91897400	-5.49178600	-0.99517900
H	-2.24383500	-6.30906700	-1.23306000
C	-4.23332700	-5.73590800	-0.66980300
H	-4.61433400	-6.75237500	-0.65324400
C	-5.08774600	-4.65664600	-0.33891800
H	-6.11827800	-4.85436800	-0.05924700
C	-4.62574000	-3.35908700	-0.36178400
H	-5.29104200	-2.54688000	-0.09580100
C	-5.07987100	-1.02900900	-2.49239800
H	-4.44914900	-1.86576300	-2.76527900
C	-6.17703400	-0.71730800	-3.26398300
H	-6.40666900	-1.31725000	-4.13942100
C	-7.00505900	0.38207100	-2.93240800
H	-7.86996200	0.61173100	-3.54723800
C	-6.70161600	1.16550500	-1.84325400
H	-7.31532000	2.02454800	-1.58741700

C -0.22321400 3.99656300 -1.08515200
C -0.02428700 4.44450000 -2.40364200
C -0.04823100 4.92181500 -0.03836300
C 0.35715800 5.75976500 -2.66966500
H -0.16024400 3.74317200 -3.22301200
C 0.32981000 6.23517700 -0.30297500
H -0.21272000 4.60804900 0.98764000
C 0.53952500 6.66129700 -1.61900500
H 0.51113400 6.08004200 -3.69634500
H 0.46077600 6.93264900 0.51969900
H 0.83627700 7.68623700 -1.82131600
I 1.53318400 -2.29412400 -1.59154400
I -3.56764600 2.74318400 2.41736100
H -0.65761800 -1.26028900 2.62545400
O -1.59151600 -1.53183200 2.84531700
H -2.05616600 -0.78179400 2.06364800
C -1.74601400 -2.94725800 2.48528600
H -1.36754400 -3.08778900 1.47155200
H -2.82072000 -3.13259800 2.49618400
C -1.00171800 -3.80323300 3.48472000
H -1.38372500 -3.64894100 4.49786000
H -1.12923500 -4.85773000 3.22005800
H 0.06943400 -3.57590200 3.47213700
B 1.50997600 -0.25260200 1.46876400
O 2.06049000 -1.07979500 2.42737300
B 3.28972100 -1.67948000 2.56606900
O 3.53079500 -2.19967800 3.81009600
H 4.37105900 -2.67662500 3.84649900
C 2.31641400 0.74836000 0.59505300
H 1.86819700 1.05193100 -0.34684100
C 3.48249200 1.32598500 0.94205400
H 3.97457800 1.03177800 1.86815300
C 4.30938600 -1.75292500 1.38999200
H 3.92653900 -1.64052100 0.37845700
C 5.64417000 -1.83458700 1.55568100
H 6.05286000 -1.94533800 2.56199300
C 6.66411400 -1.75248900 0.50189100
C 7.99214500 -2.10447200 0.80094700
C 6.36784600 -1.30500500 -0.80001900
C 8.98909800 -2.03607900 -0.17130400
H 8.23671500 -2.43800600 1.80616800
C 7.36422200 -1.23234400 -1.76904100
H 5.35885000 -0.98943000 -1.04503900
C 8.67813200 -1.60120900 -1.46120800
H 10.00797100 -2.31733600 0.07865100
H 7.11912800 -0.87744200 -2.76585000
H 9.45355200 -1.53983300 -2.21905300
C 4.19914100 2.33932200 0.15541900
C 5.57849100 2.52321600 0.35545700
C 3.54552300 3.14128000 -0.79934300
C 6.29031900 3.45862500 -0.39469700
H 6.09173800 1.91335200 1.09423300
C 4.25480200 4.08310500 -1.53954900
H 2.47450700 3.04411800 -0.94068800
C 5.63089900 4.24226600 -1.34481300
H 7.35800100 3.57897300 -0.23505300
H 3.72938200 4.70201000 -2.26112000
H 6.18218600 4.97855300 -1.92242600

Int2-B

C -2.44461500 0.27697900 0.00277300
H -1.45343600 -0.17464200 0.03246000
C -2.51682600 1.62380200 -0.03109200
H -3.49871000 2.09810800 -0.05968400
C -3.54961900 -0.68670800 0.00653700
C -3.24766500 -2.05958100 0.05157100
C -4.90337000 -0.30169400 -0.03265700
C -4.25947200 -3.01792200 0.05789100
H -2.20665000 -2.36943000 0.08204800
C -5.91328000 -1.25806700 -0.02651600
H -5.16491000 0.75094000 -0.06814900
C -5.59658600 -2.62007000 0.01886300
H -4.00496800 -4.07291900 0.09316000
H -6.95228200 -0.94380000 -0.05719700
H -6.38812500 -3.36333300 0.02359200
B -1.24709900 2.51178800 -0.02481500
O -1.30856100 3.89077800 -0.03691300
O 0.00049400 1.93584100 -0.00469800
B 1.25343800 2.53730200 0.02331700
C 2.51515900 1.63379200 0.03021600
H 3.49218100 2.11611400 0.06191200
C 2.44892600 0.28771200 -0.00585000
H 1.46076500 -0.17081400 -0.03870600
C 3.56044500 -0.67044500 -0.00790500
C 3.26909900 -2.04553600 -0.05286100
C 4.91174500 -0.27709700 0.03314400
C 4.28744500 -2.99724200 -0.05711900
H 2.23028100 -2.36295000 -0.08479800
C 5.92839000 -1.22659400 0.02902000
H 5.16560500 0.77737800 0.06842200

C	5.62194100	-2.59088600	-0.01617300
H	4.03982300	-4.05399700	-0.09228800
H	6.96506700	-0.90456300	0.06111100
H	6.41861600	-3.32870100	-0.01930100
O	1.41474700	3.89253200	0.04438000
H	0.55206700	4.34164000	0.03016600
H	-2.21075200	4.23965700	-0.04646800

TS3-B

B	1.36451400	-0.87833200	-0.46682900
C	2.59260900	-1.83748700	-0.15062300
O	0.05805500	-1.50294800	-0.16805900
C	3.34573700	-2.45065600	-1.07881300
H	2.82707000	-2.02243500	0.90002000
O	1.43500500	0.31508000	0.60262300
O	1.32895300	-0.31564300	-1.80424900
B	-0.70728400	-2.25881200	-1.03302500
C	4.49923000	-3.33549300	-0.85100800
H	3.10962800	-2.28969400	-2.13245300
C	1.50439500	1.61962700	0.18916500
C	-2.06596500	-2.83830100	-0.54315500
O	-0.32477800	-2.48250200	-2.32600800
C	5.06824100	-4.01741800	-1.94130700
C	5.07737800	-3.52200200	0.41922200
C	0.35916400	2.32300100	-0.14895300
C	2.77730800	2.24948700	0.11429500
C	-2.69045600	-2.38700400	0.56157900
H	-2.57704900	-3.56107600	-1.17900100
C	6.16302700	-4.86467500	-1.77191800
H	4.63874800	-3.87972900	-2.93046300
C	6.17061300	-4.36716200	0.58965700
H	4.67179100	-2.99351600	1.27643200
C	0.46556000	3.67768200	-0.60055300
C	-0.99701600	1.71759900	0.00916100
C	2.90510300	3.54520400	-0.31852600
I	4.52155000	1.17520800	0.72703500
C	-4.02860200	-2.72695700	1.05543300
H	-2.19376600	-1.63317800	1.16832700
C	6.71933500	-5.04504900	-0.50416700
H	6.58226900	-5.38256700	-2.62996500
H	6.60186400	-4.49491200	1.57871200
C	-0.67382700	4.44433200	-0.96621500
C	1.75651900	4.28751900	-0.69614400
C	-1.61896400	1.11049100	-1.07745000
C	-1.68865200	1.85895600	1.25096600
H	3.87906000	4.01878200	-0.37309500
C	-4.47870100	-2.12127600	2.24298100
C	-4.89725900	-3.60718800	0.38395000
H	7.57332500	-5.70208800	-0.36843500
C	-0.53759200	5.74233600	-1.40653500
H	-1.65602200	3.99141300	-0.89659000
C	1.86482800	5.62834400	-1.15312300
C	-2.97719600	0.69775800	-0.95698400
O	-0.99695600	0.92121400	-2.25989800
C	-1.08410000	2.44369400	2.39775500
C	-3.03866800	1.39615100	1.35871900
C	-5.75147900	-2.38475300	2.74683400
H	-3.82002300	-1.43245600	2.76457300
C	-6.16733900	-3.87211100	0.88825900
H	-4.57756000	-4.07929500	-0.53946000
C	0.74136200	6.34226600	-1.50258700
H	-1.41920900	6.31242100	-1.68377500
H	2.85211700	6.07639000	-1.22039200
C	-3.66927800	0.83393400	0.21865200
I	-3.93792500	-0.18813400	-2.64570700
C	-1.77094000	2.53784200	3.58850900
H	-0.06509300	2.80823900	2.32823100
C	-3.72122700	1.50711500	2.59954100
C	-6.60123800	-3.26262700	2.07073900
H	-6.07985500	-1.90485600	3.66435000
H	-6.82567700	-4.55313300	0.35656800
H	0.83164900	7.36619000	-1.85227500
H	-4.69530600	0.49378900	0.29942900
C	-3.10018300	2.06092600	3.69668700
H	-1.28879500	2.98285700	4.45403200
H	-4.74281500	1.14340600	2.66299400
H	-7.59446900	-3.46968700	2.45806300
H	-3.62731700	2.13980200	4.64256200
C	0.67042800	-1.36373500	3.30426200
C	1.10681300	-0.31592800	4.30181900
H	1.51532600	-1.92262800	2.89205400
O	-0.02100500	-0.68423800	2.20193200
H	-0.05354700	-2.06279700	3.72619300
H	0.24721300	0.25017100	4.67057700
H	1.59439900	-0.80641800	5.14949600
H	1.82272500	0.37916400	3.85172900
H	-0.21544100	-1.30245800	1.42530800
H	-0.05184600	0.66962400	-2.11412500
H	0.66828000	-0.03941700	1.59787100
H	0.43275200	-1.91328000	-2.54577700

H 2.20923700 -0.05016400 -2.10429300

TS4-B

O -1.63155400 -0.46418500 -3.00117500
B -0.75066300 -1.11052600 -2.10610200
C 0.74582500 -0.56814700 -2.24250300
H 0.88869300 0.43296200 -2.65214300
C 1.84274800 -1.27244200 -1.91094500
H 1.71222400 -2.25903600 -1.46163300
C 3.24965500 -0.86382700 -2.04402200
C 4.24126100 -1.58001800 -1.35008900
C 3.65392700 0.22854200 -2.83241600
C 5.58184300 -1.20165700 -1.41076300
H 3.94601600 -2.42912800 -0.73976300
C 4.99320500 0.60390300 -2.90042300
H 2.91113600 0.78945000 -3.39005400
C 5.96497500 -0.10378700 -2.18529100
H 6.32703700 -1.76316800 -0.85412300
H 5.28105300 1.45428900 -3.51234300
H 7.00851500 0.19256500 -2.23870400
O -0.82643200 -2.60727700 -2.09207900
H -0.19428400 -2.94854900 -2.73912700
H -0.94133800 -1.83045300 0.15997200
O -0.58164200 -2.91682900 0.35789900
H -0.57842000 -3.09333500 -0.65946900
C -1.61817600 -3.71552500 1.00452900
H -1.23895600 -4.73941700 1.05020100
H -2.51513600 -3.69475100 0.37923100
C -1.89256300 -3.15790000 2.38379200
H -2.66697800 -3.76027600 2.86866100
H -0.99340700 -3.19008500 3.00651800
H -2.24629400 -2.12447100 2.32819400
H -2.48299000 -0.92206900 -3.00744800
O -1.28882200 -0.86676100 -0.56506000
C -1.98160600 0.21089400 -0.11308800
C -1.31020900 1.30051800 0.42967500
C -3.40453300 0.21212600 -0.14681000
C -2.04699800 2.40862300 0.95462400
C 0.17680200 1.28352300 0.47775900
C -4.13496600 1.27634500 0.31898500
I -4.44730800 -1.50736800 -0.88386200
C -3.47617100 2.40150800 0.87620700
C -1.41252200 3.52428900 1.56798300
C 0.95271400 2.17117400 -0.32554300
C 0.80891300 0.36059600 1.30237000
H -5.21867800 1.26577400 0.28243800
C -4.21215000 3.50788700 1.38012500
C -2.15231800 4.57984900 2.05237100
H -0.33251900 3.53341500 1.65368800
C 0.36161100 3.08495900 -1.24239300
C 2.38053000 2.13440200 -0.23536900
C 2.22865000 0.30668000 1.33845000
O 0.02185400 -0.45409800 2.04959200
C -3.56515300 4.57843400 1.95304600
H -5.29593500 3.48709900 1.30706800
H -1.64854100 5.42166000 2.51802200
C 1.14258400 3.92893800 -1.99993900
H -0.71645800 3.10115800 -1.34927000
C 3.15850400 3.02624000 -1.02121900
C 2.99620600 1.17417900 0.60400800
I 3.19568700 -1.19073000 2.53379800
H -4.13400000 5.42018700 2.33619700
C 2.55459600 3.91131300 -1.88363100
H 0.67109500 4.61456800 -2.69787000
H 4.23991500 2.97950000 -0.93775400
H 4.07808200 1.11878500 0.63578300
H 3.15508200 4.58666500 -2.48538400
H 0.56397100 -1.12802900 2.49274400

Int6-B

O -0.62506400 -0.88978400 -3.25287500
B -0.82494900 -0.93683800 -1.90473900
C 0.13129200 -1.71684600 -0.97756400
H -0.14509100 -1.84253400 0.06677300
C 1.34441700 -2.13963900 -1.38505900
H 1.62337700 -1.98437000 -2.42705000
C 2.39365800 -2.74486500 -0.55817300
C 3.63450900 -3.04160300 -1.14797500
C 2.23294600 -2.99343400 0.81910400
C 4.68588300 -3.55675500 -0.39127000
H 3.77196600 -2.85016000 -2.20883600
C 3.28232800 -3.50688700 1.57479800
H 1.28576400 -2.77591300 1.30109000
C 4.51518800 -3.78734400 0.97484200
H 5.63833100 -3.77309200 -0.86623900
H 3.14201400 -3.68807400 2.63654300
H 5.33305000 -4.18394600 1.56905600
H -1.25716200 -0.29540200 -3.68412300
O -1.93635000 -0.25304500 -1.42440000
C -2.17827800 0.05642500 -0.12042900

C -1.33453200 0.90394400 0.58995900
C -3.36243200 -0.44495200 0.48235100
C -1.68826600 1.29482300 1.92004200
C -0.07708700 1.39008900 -0.04613600
C -3.70854800 -0.10249400 1.76450900
I -4.60978200 -1.77170900 -0.63782000
C -2.88531200 0.77900600 2.51187100
C -0.89782700 2.19940900 2.67951000
C -0.11354700 2.27646500 -1.16546500
C 1.14622500 0.92653300 0.43432600
H -4.61098000 -0.49535700 2.21971400
C -3.23618700 1.16484800 3.83371600
C -1.26643000 2.56058600 3.95615800
H 0.00037200 2.61247500 2.23526300
C -1.32982900 2.80159900 -1.68276800
C 1.10966400 2.64989400 -1.80860100
C 2.35161700 1.33202700 -0.20345600
O 1.12951600 0.08738400 1.49634000
C -2.44289400 2.03590100 4.54445200
H -4.14593500 0.76026200 4.26836600
H -0.65036900 3.25615400 4.51828700
C -1.33037400 3.63352900 -2.78015200
H -2.26328400 2.54287100 -1.19672200
C 1.07725500 3.50986300 -2.93914900
C 2.33702100 2.15717900 -1.29896300
I 4.23345400 0.62331000 0.54391300
H -2.71826100 2.32734700 5.55348500
C -0.11843800 3.99030600 -3.42104700
H -2.27085700 4.02403600 -3.15760200
H 2.01701600 3.77882500 -3.41334900
H 3.26228000 2.44638400 -1.78484600
H -0.13712000 4.64562500 -4.28644800
H 2.01806900 -0.27786000 1.64827700

TS5-B

C -2.86060600 -1.34914700 -0.59038900
C -4.07577500 -0.71165900 -0.59577400
C -1.63754100 -0.62436900 -0.50752800
C -4.14084100 0.70150900 -0.47784400
C -1.67196100 0.77154700 -0.48636400
C -2.92668300 1.45816700 -0.40753300
O -0.46213500 -1.27636800 -0.48096100
B 0.30930200 -1.33647800 0.76202100
C 1.80522500 -1.77743900 0.49305700
H 2.42260000 -2.02825000 1.35866400
C 2.38226000 -1.77324700 -0.72047400
H 1.75861500 -1.52582800 -1.57950400
O -0.39185400 -2.03894800 1.83612400
H -4.99942800 -1.27618200 -0.66312300
C -0.37642800 1.50222200 -0.50595500
C -0.06313300 2.47913300 -1.50783200
C 0.57631000 1.19131700 0.45359100
C 1.18434600 3.17973700 -1.43084700
C 1.80799100 1.88840000 0.51612600
C 2.10163500 2.87423500 -0.39188400
H 3.03997000 3.41521800 -0.34202700
O 0.33340100 0.17584300 1.34291900
C -5.39216100 1.37049700 -0.40091300
H -6.30065300 0.77829300 -0.46655700
C -5.45174500 2.73459000 -0.23080600
H -6.41164200 3.23773000 -0.16646300
C -4.25461300 3.48249000 -0.12256500
H -4.30473700 4.55565700 0.03604800
C -3.02688800 2.86379000 -0.20973200
H -2.12121500 3.45026000 -0.11500000
C -0.92373100 2.76028500 -2.60441800
H -1.85192500 2.21132500 -2.70195100
C -0.58504900 3.70627600 -3.54609300
H -1.25517100 3.90028600 -4.37818200
C 0.63141300 4.42344900 -3.44541100
H 0.88160900 5.17034300 -4.19262900
C 1.49932300 4.15940300 -2.41140600
H 2.44557900 4.68679000 -2.33107400
C 3.78831200 -2.05515500 -1.04654500
C 4.17030900 -2.15287200 -2.39656000
C 4.78462700 -2.21457800 -0.06440800
C 5.49205200 -2.41438900 -2.75642600
H 3.41450800 -2.02300400 -3.16700300
C 6.10464700 -2.47512200 -0.42226500
H 4.52387400 -2.12163700 0.98502400
C 6.46624000 -2.57861700 -1.76981600
H 5.76136200 -2.48795400 -3.80635600
H 6.85812800 -2.59239300 0.35173600
H 7.49736600 -2.77963500 -2.04543600
I -2.78787000 -3.48236500 -0.72269700
I 3.21049600 1.40798900 2.05342900
H -1.05710700 -1.01257100 2.92288100
O -1.19876100 -0.03542000 3.13487400
H -0.47750800 0.28687900 2.25525200
C -2.60336300 0.31840300 2.91653300

H	-2.95949500	-0.20276500	2.02602600
H	-2.60972100	1.39234000	2.72393400
C	-3.41642500	-0.04248800	4.13958200
H	-3.04529800	0.47969000	5.02620900
H	-4.45992800	0.24602200	3.97676800
H	-3.38738200	-1.12094000	4.32675000
H	0.12023000	-2.79442800	2.14891700

TS6-B

C	-2.82748700	-0.29946400	-1.01808200
H	-2.74368000	-0.26607000	-2.10449100
C	-1.85457900	-0.93002100	-0.33067500
H	-1.92774200	-0.97735200	0.75631500
C	-4.01935600	0.36838300	-0.48117400
C	-4.95379600	0.91252000	-1.38013500
C	-4.27190500	0.49364500	0.89839200
C	-6.10417200	1.55310900	-0.92270300
H	-4.76910000	0.82615200	-2.44750100
C	-5.41951800	1.13348700	1.35537900
H	-3.56323700	0.09228100	1.61580100
C	-6.34189300	1.66542800	0.44785700
H	-6.81314700	1.96453000	-1.63499700
H	-5.59750600	1.22128500	2.42317500
H	-7.23597300	2.16478700	0.80878200
B	-0.61317400	-1.56443600	-1.01985600
O	-0.50090100	-1.62457700	-2.37521300
O	0.40295900	-2.05780000	-0.21505900
B	1.84809100	-2.32762800	-0.65380500
C	2.70014200	-0.97132100	-0.60798300
H	3.77187500	-1.05763800	-0.81150900
C	2.21146900	0.25233900	-0.34198800
H	1.14519400	0.34427100	-0.13146000
C	2.93476100	1.53333700	-0.28225600
C	2.21464300	2.70284100	0.02001400
C	4.31944900	1.65116100	-0.50595100
C	2.84703800	3.94314000	0.09546500
H	1.14440500	2.62959900	0.19621500
C	4.95223400	2.88876900	-0.43113300
H	4.90448300	0.76694300	-0.73905400
C	4.22074700	4.04233400	-0.13010800
H	2.26728200	4.83117800	0.33041500
H	6.02199700	2.95612700	-0.60786600
H	4.71816500	5.00580800	-0.07214200
O	1.78016100	-2.97531800	-1.96015700
O	2.40435800	-3.27601800	0.38205300
H	1.64322300	-2.95545700	1.59174800
O	0.83790700	-2.48186700	2.09275300
C	1.30821400	-1.33153500	2.87119600
H	1.68084700	-1.73847300	3.81324000
H	2.13277900	-0.86159700	2.32901400
C	0.16157000	-0.36909100	3.08530800
H	-0.67330000	-0.86021200	3.59421900
H	0.50242200	0.46537100	3.70555600
H	-0.19423200	0.03843700	2.13416400
H	2.21463700	-4.18140200	0.09016000
H	2.58284500	-2.78885400	-2.46481400
H	0.33501200	-2.10406800	-2.57445900

TS7-B

C	3.06272800	-1.18904600	0.66702100
H	2.83609700	-0.38027700	1.36272900
C	2.04176800	-1.96818600	0.27231300
H	2.24969100	-2.77679900	-0.43417000
C	4.47532900	-1.25781800	0.25993400
C	5.35905200	-0.25578000	0.69971000
C	4.99273900	-2.27649500	-0.56253300
C	6.70355000	-0.26230100	0.32996200
H	4.97751600	0.53725300	1.33812900
C	6.33494500	-2.28535000	-0.93138700
H	4.33781300	-3.06855600	-0.91206700
C	7.19888300	-1.27811100	-0.48897800
H	7.36392400	0.52524000	0.68178800
H	6.71197100	-3.08227100	-1.56628200
H	8.24532100	-1.28836100	-0.77874900
B	0.52476000	-1.77458100	0.73416000
O	-0.34578400	-1.54137300	-0.53080100
B	-0.50380500	-0.36728400	-1.23341000
O	-1.53597600	-0.38350400	-2.13505700
O	0.37615900	-0.70238700	1.69095300
B	-0.67496100	0.11307200	1.96843600
C	-2.12809100	-0.14474300	1.45292900
H	-2.43254000	-1.16370600	1.22127700
C	0.42273200	0.85653200	-0.98791300
H	1.43571700	0.66436800	-0.64337300
C	0.00248100	2.13198200	-1.08869000
H	-1.02629800	2.32657100	-1.39681700
C	-2.99402500	0.85062200	1.18345900
H	-2.69764600	1.87186600	1.42281600
C	-4.32360800	0.72630400	0.57087600
C	-5.18020800	1.84115300	0.56651500

C	-4.77502400	-0.46304300	-0.03299900
C	-6.45238700	1.76927800	0.00100700
H	-4.83931600	2.76875700	1.01870400
C	-6.04446500	-0.53402200	-0.59878900
H	-4.12283700	-1.32922800	-0.07501400
C	-6.89088400	0.57947100	-0.58233600
H	-7.09942100	2.64149100	0.01360700
H	-6.37459700	-1.45878100	-1.06312500
H	-7.87954100	0.52016900	-1.02744600
C	0.77423800	3.34458700	-0.78780800
C	0.15938900	4.59863500	-0.94547900
C	2.10874100	3.30765700	-0.34026200
C	0.84963900	5.77808200	-0.66994800
H	-0.87142600	4.64086700	-1.28706700
C	2.79768000	4.48455300	-0.06568400
H	2.60873800	2.35382100	-0.20607600
C	2.17258700	5.72548800	-0.22911200
H	0.35500400	6.73622500	-0.79878900
H	3.82669100	4.43674500	0.27836600
H	2.71375900	6.64155800	-0.01238600
O	-0.45510900	1.24244200	2.72511100
H	-1.59451100	0.44035500	-2.63792600
O	-0.04811900	-3.06453000	1.28975600
H	-1.23916700	-3.43275300	0.56972700
O	-1.90954300	-3.31822600	-0.24490700
H	-1.31658500	-2.43712800	-0.58087100
C	-1.78354900	-4.43024800	-1.18492600
H	-2.29486900	-4.10922100	-2.09461300
H	-0.72205200	-4.57784700	-1.40891300
C	-2.41240600	-5.66987000	-0.58989600
H	-3.46730000	-5.49774400	-0.35734600
H	-2.34471000	-6.49349000	-1.30737300
H	-1.89382300	-5.97248700	0.32561600
H	0.49264200	1.34128400	2.90097800
H	-0.15223600	-2.96929900	2.24637400

Int7-B

C	0.91815800	2.11804000	0.54432600
H	0.46126000	2.49168700	1.46060300
C	0.56834500	0.88665000	0.11971200
H	1.01210700	0.50192800	-0.79663000
C	1.86314300	3.04502000	-0.08702300
C	2.00418500	4.33786800	0.44776000
C	2.64432600	2.69579700	-1.20530200
C	2.88521900	5.25746800	-0.11851700
H	1.41089600	4.61645700	1.31450800
C	3.52524900	3.61286100	-1.76909800
H	2.56786900	1.69874900	-1.62686700
C	3.64866700	4.89812000	-1.23026500
H	2.97726500	6.25179200	0.30813500
H	4.12228200	3.32616800	-2.62976200
H	4.33845600	5.61045600	-1.67272100
B	-0.42714900	-0.01069300	0.90159400
O	-1.02650000	0.47068000	2.03594900
B	-2.21154900	0.16434400	2.66605600
O	-2.15688700	0.13990600	4.03294000
O	-0.73954500	-1.28327400	0.50042200
B	-0.29408200	-2.15179200	-0.47399000
C	1.19859100	-2.51910300	-0.67788700
H	1.45201000	-3.16453100	-1.51898500
C	-3.53434400	-0.08216300	1.88141800
H	-4.39272500	-0.48840100	2.41900100
C	-3.67285000	0.19182200	0.56902000
H	-2.82033700	0.61320100	0.03645200
C	2.18695600	-2.08347800	0.12961600
H	1.92248600	-1.44160700	0.96933400
C	3.62533800	-2.34682800	0.01734300
C	4.49893800	-1.70592300	0.91374200
C	4.17517000	-3.20487500	-0.95373800
C	5.87602600	-1.90811000	0.84274500
H	4.08545900	-1.04055300	1.66680600
C	5.54947700	-3.40846100	-1.02312300
H	3.52223300	-3.71658200	-1.65337200
C	6.40632500	-2.76082100	-0.12653500
H	6.53362100	-1.40169300	1.54288300
H	5.95718600	-4.07526300	-1.77713700
H	7.47841600	-2.92294500	-0.18422700
C	-4.85048900	-0.01594400	-0.28063200
C	-4.74426300	0.26145200	-1.65536400
C	-6.07962600	-0.49489700	0.21097100
C	-5.82406600	0.06353200	-2.51406600
H	-3.80087100	0.63365000	-2.04597600
C	-7.15789400	-0.69048100	-0.64576700
H	-6.19103000	-0.71220200	1.26842300
C	-7.03548900	-0.41359000	-2.01174100
H	-5.72018600	0.28217300	-3.57258700
H	-8.09933800	-1.05954700	-0.24985500
H	-7.87978800	-0.56804100	-2.67669000
O	-1.22438400	-2.75098400	-1.27807900
H	-3.02603800	-0.01856200	4.42662800

H -2.11935100 -2.43998500 -1.07168700

TS8-B

C 2.15849900 2.67775100 -0.53975100
H 2.60639000 1.83454700 -0.01404000
C 0.96321600 2.48180100 -1.13210600
H 0.48885700 3.30839400 -1.66173000
B 0.21727700 1.11976000 -1.08838300
O 0.82263400 0.05033700 -0.41414400
B 0.32325700 -1.22373100 -0.52105000
O -0.91771600 -1.40561900 -1.09981600
O -0.97823000 0.96368000 -1.69447400
B -1.81882500 -0.20695400 -1.52219000
C -2.95392100 -0.01125300 -0.41194400
H -3.58774900 -0.87364500 -0.18288200
C 1.12667200 -2.44868300 -0.02393900
H 0.68282900 -3.44101800 -0.10866900
C 2.35933000 -2.32258800 0.50971300
H 2.77943400 -1.31940700 0.57933400
C -3.18504000 1.13244100 0.25459000
H -2.55467100 1.99116400 0.02007500
O -2.38303700 -0.66455700 -2.82840900
H -2.28628800 -2.18392500 -2.87246300
O -1.90791000 -3.06369100 -2.47427400
H -1.31963400 -2.46260000 -1.70963200
C -2.98572900 -3.81734800 -1.83307500
H -2.49409300 -4.62249900 -1.28400700
H -3.49434600 -3.16157600 -1.11900400
C -3.93361500 -4.34612200 -2.88565500
H -3.40726100 -4.98769800 -3.59804400
H -4.72121100 -4.93363900 -2.40390500
H -4.40863700 -3.52595100 -3.43397700
C 3.23379000 -3.38211500 1.02216800
C 4.50094500 -3.03026600 1.52049700
C 2.86354700 -4.74040000 1.03888200
C 5.37207300 -3.99823800 2.01693500
H 4.79764600 -1.98499900 1.51385100
C 3.73269500 -5.70662900 1.53464200
H 1.89031700 -5.03919500 0.66313500
C 4.99067100 -5.34071200 2.02558100
H 6.34630900 -3.70522100 2.39652600
H 3.43101700 -6.74981800 1.54041200
H 5.66606100 -6.09803000 2.41195800
C 2.96128500 3.90594200 -0.50388300
C 4.17813700 3.89852500 0.20129000
C 2.57265700 5.09663700 -1.14699900
C 4.98020300 5.03663000 0.26596600
H 4.49072700 2.98602900 0.70209300
C 3.37312000 6.23273500 -1.08326300
H 1.64059900 5.13062500 -1.70156600
C 4.58036700 6.20912700 -0.37681000
H 5.91560800 5.00820800 0.81691300
H 3.05734900 7.14178400 -1.58674200
H 5.20230600 7.09799200 -0.33019100
C -4.20213000 1.39472500 1.28623900
C -4.30404700 2.68883400 1.82716200
C -5.08573700 0.40929400 1.76616000
C -5.25054700 2.99247900 2.80502500
H -3.62847200 3.46181500 1.47001600
C -6.03087500 0.71050600 2.74258600
H -5.02902100 -0.60141100 1.37407600
C -6.12003200 2.00381100 3.26782200
H -5.30834800 4.00060300 3.20533500
H -6.70156400 -0.06638500 3.09874800
H -6.85815300 2.23501600 4.02992900
H -3.26368600 -0.28703700 -2.95549900

TS8(*S*)

C -2.91980300 4.55293700 -3.04145600
C -1.85686000 4.78542200 -2.19956800
C -1.37368400 3.76264200 -1.33925100
C -2.01851800 2.48337200 -1.32747300
C -3.09748000 2.27146600 -2.22989400
C -3.53447100 3.27789400 -3.06244600
H 0.22881600 4.96227300 -0.51599100
H -3.27992700 5.33810400 -3.69922600
H -1.35966000 5.75127500 -2.18501200
C -0.24664800 3.98768900 -0.50643800
C -1.53464400 1.46706600 -0.43911400
H -3.57136000 1.29847200 -2.26830600
H -4.35577700 3.08923100 -3.74741500
C -0.37634300 1.69513200 0.29771900
C 0.24768300 2.97051800 0.26961600
C -2.22331400 0.15634400 -0.29073300
C -3.60581900 0.07097000 0.08116000
C -1.49257200 -1.01614700 -0.47049000
C -4.37373100 1.20860900 0.45668500
C -4.24338300 -1.21153200 0.12225400
C -2.14957300 -2.27606300 -0.44183300
C -5.69862100 1.08522900 0.81085700

H	-3.90094000	2.18235000	0.47570000
C	-5.61660600	-1.30478100	0.47652500
C	-3.49069800	-2.37703700	-0.17310000
C	-6.33451900	-0.17978700	0.80917100
H	-6.26089400	1.96793600	1.10049300
H	-6.08204000	-2.28627600	0.48942400
H	-3.98471200	-3.34214000	-0.15673600
H	-7.38194500	-0.25921800	1.08363300
O	-0.15144400	-0.98275900	-0.67452200
O	0.15756200	0.70403100	1.05697900
B	0.70993400	-0.42148800	0.32504400
C	2.21573200	-0.34929400	-0.10895600
H	2.90272000	0.19036700	0.53975600
C	2.68422600	-0.89571200	-1.24645900
H	1.98462200	-1.43654700	-1.88327700
C	4.06086000	-0.84562500	-1.75590200
C	4.36675300	-1.51517500	-2.95442300
C	5.09689600	-0.15590300	-1.09676900
C	5.65965300	-1.50377000	-3.47660700
H	3.57583700	-2.04910900	-3.47487300
C	6.38715700	-0.14329200	-1.61806700
H	4.88802200	0.37631900	-0.17408700
C	6.67624600	-0.81764900	-2.80968900
H	5.87295200	-2.02918700	-4.40311700
H	7.17284500	0.39509200	-1.09561600
H	7.68448400	-0.80518000	-3.21309600
I	-1.01321500	-4.05093100	-0.82174400
I	1.98469600	3.29753700	1.47391300
C	0.70837900	-1.73680900	1.77677400
H	0.67722800	-2.70865100	1.28239500
C	1.87744600	-1.60626300	2.73963900
F	2.07452200	-0.33298900	3.12473900
F	1.69764700	-2.33659800	3.86433800
F	2.99007400	-2.04953800	2.13908400
N	-0.47514700	-1.51715700	2.42578200
N	-1.45628200	-1.18835000	2.85016600

TS8(*R*)

C	1.31475900	5.13723200	2.88176100
C	0.33576800	5.09962200	1.91595700
C	0.22637500	3.99028900	1.03449200
C	1.16477900	2.91225600	1.13285000
C	2.14666100	2.97371800	2.15974000
C	2.21797700	4.05500300	3.00990700
H	-1.51030700	4.75930300	-0.00494000
H	1.38782100	5.98580700	3.55510800
H	-0.37962700	5.91128900	1.81753800
C	-0.81492100	3.93074600	0.07122600
C	1.05406900	1.80690700	0.22396400
H	2.83783700	2.14891700	2.27969800
H	2.97183800	4.07461500	3.79134300
C	-0.03807800	1.73790000	-0.63323500
C	-0.95345700	2.81941600	-0.72019200
C	2.07140900	0.72224200	0.17466100
C	3.46124900	1.03430600	-0.01964700
C	1.68404000	-0.61305800	0.29123600
C	3.91704800	2.34117200	-0.34954600
C	4.43866300	-0.00618500	0.08075200
C	2.67805000	-1.62514400	0.41782000
C	5.25765200	2.60393200	-0.52688300
H	3.19466200	3.13706000	-0.47830400
C	5.81591500	0.29729700	-0.09063900
C	4.01453200	-1.33557100	0.33516500
C	6.22241700	1.57879300	-0.38278000
H	5.57659500	3.60894000	-0.78685700
H	6.53863800	-0.50848200	0.00294000
H	4.75956000	-2.11685800	0.43663800
H	7.27609700	1.80312800	-0.51775400
O	0.38323900	-0.99112200	0.34432100
O	-0.23478000	0.61489200	-1.37776900
B	-0.62199900	-0.51572500	-0.55207300
C	-2.07950300	-0.55893800	0.02251100
H	-2.88301900	-0.18404200	-0.60925100
C	-2.37166300	-1.01010800	1.25685200
H	-1.55212100	-1.37988200	1.87233900
C	-3.68966600	-1.05812100	1.90189500
C	-3.78119700	-1.53679100	3.22141400
C	-4.87383700	-0.64503600	1.26115900
C	-5.00780700	-1.60192600	3.88108100
H	-2.87501300	-1.85888200	3.72780000
C	-6.09839400	-0.70978000	1.91916200
H	-4.83462500	-0.27133000	0.24285600
C	-6.17251800	-1.18816400	3.23212400
H	-5.05419100	-1.97496700	4.90017200
H	-7.00095300	-0.38674000	1.40814500
H	-7.13010300	-1.23714300	3.74208000
I	2.07392800	-3.64884700	0.75414900
I	-2.56175500	2.70848300	-2.12617600
C	-0.74920700	-1.78685700	-2.07652300
N	0.54004900	-1.98955800	-2.45683800

N	1.63999900	-2.06978800	-2.65058100
H	-1.25234300	-1.17215600	-2.82279600
C	-1.48867100	-3.08081900	-1.78869300
F	-1.24349200	-4.02868600	-2.72388400
F	-2.80638300	-2.82543700	-1.79720500
F	-1.16885500	-3.61269500	-0.59946500

Int8(*S*)

C	1.51536400	5.13346500	2.86051900
C	0.47761800	5.05643200	1.96058700
C	0.32183600	3.91624900	1.12652300
C	1.27278300	2.84773900	1.20265900
C	2.31789400	2.95104600	2.16222500
C	2.43440000	4.06192000	2.96789400
H	-1.48419800	4.63316500	0.17404300
H	1.62425900	6.00552100	3.49810800
H	-0.24857400	5.86043200	1.87925600
C	-0.77533900	3.81453800	0.23229000
C	1.11511900	1.71199900	0.34239100
H	3.02233600	2.13503900	2.26566000
H	3.23683600	4.11408400	3.69783400
C	-0.01986800	1.60532700	-0.45748000
C	-0.94810200	2.67914900	-0.51813300
C	2.12291600	0.61904200	0.28584100
C	3.49969700	0.88081700	-0.02039700
C	1.70439900	-0.69540400	0.48730100
C	3.97188800	2.16605600	-0.40830900
C	4.44563400	-0.19438800	0.02199700
C	2.66428000	-1.74396100	0.52818300
C	5.30223600	2.37804900	-0.69405000
H	3.26814100	2.98452100	-0.49150400
C	5.81474700	0.05857800	-0.26307900
C	3.99911800	-1.50663200	0.32424900
C	6.23943200	1.32041000	-0.60865000
H	5.63523300	3.36705900	-0.99454900
H	6.51537900	-0.77025500	-0.21291900
H	4.72141400	-2.31466600	0.36176800
H	7.28618200	1.50535500	-0.82976000
O	0.39680100	-0.99965800	0.65615200
O	-0.23440700	0.47742800	-1.18110400
B	-0.57269600	-0.70003500	-0.37932500
C	-2.05700700	-0.79579200	0.14848400
H	-2.85351500	-0.48451900	-0.52422100
C	-2.37767800	-1.19666100	1.39191800
H	-1.57097700	-1.50802500	2.05505800
C	-3.71849100	-1.25410400	1.99086200
C	-3.85646400	-1.74207600	3.30279900
C	-4.88025800	-0.83796700	1.31212500
C	-5.10650600	-1.81981900	3.91589100
H	-2.96786100	-2.06245400	3.84062700
C	-6.12810300	-0.91451700	1.92377200
H	-4.80414600	-0.44712600	0.30231400
C	-6.24893800	-1.40674900	3.22807400
H	-5.18821700	-2.20132700	4.92969800
H	-7.01219500	-0.58744400	1.38374300
H	-7.22441900	-1.46440900	3.70196300
I	1.98761700	-3.74415700	0.88642600
I	-2.63794000	2.52412000	-1.82088300
C	-0.40437500	-2.03232000	-1.62481100
H	-0.29443300	-2.96889700	-1.07407300
C	-1.52191200	-2.12958900	-2.66051100
F	-1.82283300	-0.93651000	-3.19507400
F	-1.18991900	-2.95348100	-3.67934800
F	-2.61673400	-2.62564500	-2.06952400
N	0.79736900	-1.77861300	-2.26808500
N	1.77087600	-1.42144000	-2.67649500

Int8(*R*)

C	-0.61519700	5.16480900	2.73490100
C	-1.47781600	4.79464700	1.72904200
C	-1.16270600	3.71355200	0.86207600
C	0.08161000	3.02059900	1.01753400
C	0.93705700	3.41563400	2.08290300
C	0.59667400	4.45661900	2.91807100
H	-3.00751100	3.84271600	-0.26422100
H	-0.86632800	5.98794500	3.39689500
H	-2.42165900	5.31364900	1.58695900
C	-2.07546000	3.30149900	-0.14449700
C	0.39495000	1.94169500	0.12526300
H	1.86254400	2.87697700	2.24423100
H	1.26265300	4.73335300	3.73001000
C	-0.56659000	1.50076600	-0.77667600
C	-1.78857100	2.20898100	-0.92215400
C	1.72202300	1.26897900	0.13588700
C	2.92578700	2.03805300	-0.02075800
C	1.80712400	-0.11923700	0.26789000
C	2.91693500	3.42041500	-0.35967200
C	4.19749600	1.39928400	0.13031700
C	3.08750200	-0.72244700	0.44362500
C	4.09094200	4.12746600	-0.49902700

H 1.97018300 3.91851700 -0.52532800
C 5.39182700 2.15687600 -0.00338400
C 4.24593000 0.00747200 0.39710600
C 5.34378000 3.49809800 -0.30617600
H 4.05406900 5.17939100 -0.76648900
H 6.34351000 1.64940900 0.12799500
H 5.20940300 -0.46981800 0.53758300
H 6.26032900 4.07041700 -0.41190400
O 0.72003000 -0.91911000 0.29294000
O -0.33784500 0.37193600 -1.49610900
B -0.38334800 -0.82509900 -0.64743800
C -1.77923000 -1.15578000 0.01635500
H -2.66349500 -1.13132400 -0.61941300
C -1.92805700 -1.41560300 1.32737400
H -1.03140600 -1.44744100 1.94550600
C -3.18201600 -1.66181500 2.05243300
C -3.12269500 -2.00480100 3.41516600
C -4.44967900 -1.56603500 1.44676400
C -4.28293800 -2.25483000 4.14715500
H -2.15110500 -2.07732800 3.89706300
C -5.60830200 -1.81369100 2.17716700
H -4.52694400 -1.28900000 0.40006200
C -5.53191900 -2.16106800 3.53054200
H -4.21179000 -2.52104800 5.19792200
H -6.57703700 -1.73291300 1.69213700
H -6.43833400 -2.35313100 4.09715400
I 3.20940200 -2.82844300 0.80934200
I -3.19507500 1.55419900 -2.39647900
C -0.16376500 -2.07326200 -1.93396400
N 1.13787800 -1.92456000 -2.38485600
N 2.19928000 -1.68405500 -2.62319300
H -0.82497600 -1.77099600 -2.74986300
C -0.44493200 -3.51990100 -1.53103700
F -1.77354200 -3.70240500 -1.53528000
F 0.01980800 -3.82065600 -0.31280000
F 0.10252000 -4.39727800 -2.40086800

TS9(*S*)

C 2.82035300 4.51916600 3.11596700
C 1.77078200 4.75208500 2.25752200
C 1.31861100 3.73830200 1.37008300
C 1.98058800 2.46819700 1.34764100
C 3.04439100 2.25506900 2.26809700
C 3.45159700 3.25205900 3.12664400
H -0.28108500 4.93115700 0.53195900
H 3.15696900 5.29729400 3.79427000
H 1.26061900 5.71129300 2.25013600
C 0.20620700 3.96247300 0.51792900
C 1.52813100 1.45956000 0.43415700
H 3.53005500 1.28769200 2.29929900
H 4.26177500 3.06187100 3.82438700
C 0.37681100 1.68049500 -0.31983200
C -0.25831400 2.95161000 -0.28409900
C 2.24305400 0.16462700 0.27611200
C 3.63511000 0.10669800 -0.06546900
C 1.52145200 -1.02025500 0.42276300
C 4.39543600 1.26162000 -0.40136200
C 4.29391800 -1.16481200 -0.11193300
C 2.20113600 -2.26906800 0.36928700
C 5.73162000 1.16508800 -0.72074200
H 3.90797500 2.22821900 -0.41738300
C 5.67737700 -1.23090800 -0.43003100
C 3.54939400 -2.34768500 0.13363500
C 6.38723800 -0.08956000 -0.72317000
H 6.28723100 2.06142000 -0.98013500
H 6.15814000 -2.20496500 -0.44851200
H 4.05548400 -3.30615500 0.10027500
H 7.44284300 -0.14852800 -0.97024000
O 0.18753000 -0.99967500 0.63029400
O -0.12933900 0.69931800 -1.10303900
B -0.69187900 -0.45583100 -0.41189800
C -2.18882400 -0.31475500 0.14314000
H -2.84444400 0.39454200 -0.35625100
C -2.65587900 -1.06356200 1.16810300
H -1.98062900 -1.79198600 1.61583400
C -3.97364300 -0.97914600 1.79668100
C -4.23467400 -1.77252100 2.93012000
C -4.99827900 -0.13889800 1.31585800
C -5.47382700 -1.72932900 3.56538000
H -3.45110000 -2.42359400 3.30825000
C -6.23471300 -0.09673400 1.95091000
H -4.82438400 0.47867100 0.44063700
C -6.47824900 -0.89023000 3.07809300
H -5.65645100 -2.34839800 4.43871000
H -7.01469900 0.55470500 1.56786800
H -7.44550100 -0.85404000 3.57035800
I 1.06178300 -4.06303500 0.62374800
I -1.96646300 3.29236700 -1.52821100
C -1.09401500 -1.60399600 -1.50691100
H -1.20146600 -2.60747400 -1.09606400

C -2.09869100 -1.38111600 -2.63754300
F -2.00000300 -0.15467700 -3.17352600
F -1.92133300 -2.27395800 -3.63482000
F -3.35178400 -1.53834400 -2.17579900
N 0.36882500 -1.83660100 -2.44022700
N 1.39889300 -1.44316000 -2.58267500

TS9(*R*)

C 0.28040200 5.17605400 2.95656900
C -0.62413900 4.98870500 1.93690000
C -0.49543200 3.89059100 1.04383900
C 0.60445000 2.98434600 1.19055500
C 1.50738200 3.19725600 2.26872900
C 1.34794800 4.26183600 3.12773700
H -2.26461700 4.37928100 -0.10476300
H 0.17189500 6.01431000 3.63808300
H -1.45916800 5.67069900 1.80262300
C -1.44867700 3.67484700 0.01423200
C 0.73314500 1.88995600 0.27419800
H 2.32290100 2.50019600 2.41787400
H 2.04558100 4.39899500 3.94863300
C -0.27798400 1.64605900 -0.64968000
C -1.34781100 2.56962500 -0.79224200
C 1.92687900 1.00241700 0.25484800
C 3.24098900 1.55657400 0.08863800
C 1.76225700 -0.38335000 0.33619400
C 3.47056200 2.93541000 -0.18248600
C 4.38235500 0.69563500 0.16019200
C 2.92036200 -1.21324700 0.40275900
C 4.74778700 3.43043800 -0.32621000
H 2.62499700 3.60216300 -0.29141600
C 5.68919100 1.23675200 0.02293900
C 4.18881500 -0.69613900 0.34264300
C 5.87328200 2.58003100 -0.20857700
H 4.89227100 4.48550300 -0.53949200
H 6.53819600 0.56231800 0.09322400
H 5.05543300 -1.34513100 0.40387000
H 6.87411500 2.98679500 -0.31691800
O 0.54178900 -0.95732500 0.39950400
O -0.24103100 0.51808600 -1.40038900
B -0.48034000 -0.69921600 -0.61682600
C -1.98149800 -0.82316100 -0.04946400
H -2.77093000 -0.34985800 -0.62747400
C -2.27957700 -1.45160100 1.11007900
H -1.47293700 -1.95043300 1.64462800
C -3.58020000 -1.50638800 1.77529900
C -3.67611800 -2.16803600 3.01443600
C -4.74490900 -0.92606900 1.23280600
C -4.89208000 -2.25409200 3.68881200
H -2.78348000 -2.61632500 3.44201000
C -5.95761000 -1.01183500 1.90748800
H -4.69809900 -0.40669700 0.28116100
C -6.03737100 -1.67581500 3.13739100
H -4.94673700 -2.77066900 4.64241400
H -6.84649400 -0.56061400 1.47654700
H -6.98691400 -1.74019500 3.66031600
I 2.69203800 -3.32989200 0.59922000
I -2.80941500 2.23638800 -2.32192900
C -0.68259600 -1.89422800 -1.69669700
N 0.86829100 -1.94901800 -2.54255800
N 1.80810800 -1.37918500 -2.70307900
H -1.28810700 -1.60644600 -2.55863700
C -0.97720800 -3.35397500 -1.35446700
F -0.37007400 -4.19897000 -2.21289200
F -0.60782300 -3.70255000 -0.11578100
F -2.30673300 -3.55874000 -1.45612600

TS9$_{Int3}$

C -0.09110700 -0.49298000 0.69818800
H -0.25076200 0.32735500 1.39897100
C -1.11227000 -0.89918600 -0.08697800
H -0.91613000 -1.70661000 -0.79262700
C -2.49489700 -0.41829600 -0.06716800
C -3.47485400 -1.13576900 -0.77828400
C -2.88847700 0.74255600 0.62738900
C -4.80443000 -0.72027300 -0.78462400
H -3.18113100 -2.02850000 -1.32372400
C -4.21572900 1.15786700 0.61875400
H -2.14648400 1.32793400 1.16071800
C -5.18027600 0.42750000 -0.08394300
H -5.54639800 -1.28935900 -1.33666800
H -4.50169000 2.05732800 1.15587600
H -6.21506500 0.75613900 -0.08928800
C 1.80739200 -0.04992400 -0.52977700
H 1.35385500 -0.19636700 -1.51116000
C 1.91634200 1.44164400 -0.23054100
F 2.32087200 1.67548800 1.03714200
F 2.79335800 2.06015200 -1.04703500
F 0.72413800 2.04969800 -0.39117500
N 3.41667200 -0.50992100 -0.93457400

N	4.03887400	-1.43160400	-0.82055000
B	1.40129000	-1.17765200	0.63827800
O	1.48075700	-2.45519500	-0.00429400
O	2.16678200	-1.13288400	1.84460900
H	1.97489000	-0.34222300	2.36317600
H	1.73787100	-3.10900800	0.65820600

$TS9_{Int2\text{-}B}$

C	-2.01726500	-0.02605300	0.70678600
H	-3.05287800	0.03873300	1.04142600
C	-1.54384500	-1.20236700	0.23684300
H	-0.51313900	-1.22517300	-0.11603400
C	-2.23817500	-2.48840600	0.19226000
C	-1.50537900	-3.64282300	-0.14215300
C	-3.61457700	-2.62269900	0.46203400
C	-2.12079700	-4.89108000	-0.19551600
H	-0.44421600	-3.54923600	-0.35601200
C	-4.22825000	-3.86905500	0.40650000
H	-4.20399300	-1.74422600	0.70359400
C	-3.48459500	-5.00857000	0.08000600
H	-1.53877000	-5.77078400	-0.45293500
H	-5.29067400	-3.95600000	0.61341000
H	-3.96820900	-5.97966400	0.03667600
C	-1.60795000	1.71757500	-0.76897900
H	-1.17379400	1.13096500	-1.57910300
C	-3.05560900	2.07415700	-1.09362800
F	-3.67472800	2.66857600	-0.05221200
F	-3.14806400	2.91227100	-2.14476200
F	-3.75250800	0.96347400	-1.40053100
N	-0.75853900	3.20680700	-1.06785100
N	0.09045900	3.79690900	-0.65246100
B	-1.10521800	1.31766300	0.76143200
O	0.30986300	1.08950900	0.67027700
O	-1.44031600	2.28521800	1.78248700
H	-2.37258800	2.25231200	2.03042500
B	1.24724100	1.22716100	1.65870200
O	0.95508100	1.76301600	2.88620300
H	0.02178300	2.05595300	2.86591500
C	2.70990600	0.75067500	1.38472700
H	3.45062000	0.86221100	2.17766800
C	3.08736500	0.21196700	0.20909300
H	2.32777400	0.11644100	-0.56730600
C	4.41630600	-0.27574700	-0.18182900
C	4.59284000	-0.80050500	-1.47463200
C	5.52983400	-0.24506000	0.67927400
C	5.83295700	-1.27719500	-1.89670400
H	3.74215800	-0.83086400	-2.15050100
C	6.76794700	-0.72077200	0.25895200
H	5.42340100	0.15326600	1.68312500
C	6.92675500	-1.23899000	-1.03073600
H	5.94517200	-1.67753300	-2.90004600
H	7.61490100	-0.68863300	0.93819000
H	7.89474600	-1.60904200	-1.35496000

$TS9_{Int6\text{-}B}$

O	0.83694300	-0.77013700	-1.84118700
B	0.58034100	-1.32054800	-0.54179600
C	-0.92941000	-1.55241500	-0.03830700
H	-1.68824100	-1.77591400	-0.78594500
C	-1.26602100	-1.58371500	1.27282500
H	-0.47439000	-1.43755100	2.00565400
C	-2.60387500	-1.74090200	1.83596200
C	-2.74630800	-1.80944500	3.23390900
C	-3.76538900	-1.79229000	1.03925800
C	-4.00397700	-1.93308800	3.82079300
H	-1.85790700	-1.75530500	3.85627900
C	-5.02038700	-1.90608700	1.62586700
H	-3.68350100	-1.72138400	-0.04024500
C	-5.14584000	-1.97853300	3.01891900
H	-4.09379300	-1.98458800	4.90176300
H	-5.90700200	-1.93551300	0.99933900
H	-6.12850500	-2.06723800	3.47256400
C	0.88655700	-2.95586500	-0.34992900
H	1.27612300	-3.13521000	0.65374500
C	0.03872600	-4.17088300	-0.74320300
F	-0.73998100	-3.95895300	-1.82293500
F	0.81516200	-5.23947600	-1.01988600
F	-0.77101200	-4.52065900	0.27294800
N	2.34336000	-3.20187800	-1.27907000
N	2.95179600	-2.64993800	-2.03374600
O	1.45438100	-0.69443900	0.45877000
C	1.99264700	0.53439500	0.35182400
C	1.22208700	1.69174300	0.25514800
C	3.41343100	0.66698900	0.41869200
C	1.84856200	2.97313300	0.13805000
C	-0.26442900	1.62468400	0.36755700
C	4.03915500	1.88260300	0.32284200

I	4.60662300	-1.08232400	0.74908100
C	3.27574900	3.06672200	0.15742600
C	1.09853900	4.17436600	0.00987400
C	-0.88138900	1.63446500	1.65417500
C	-1.04564200	1.59675000	-0.77932600
H	5.11996100	1.95597300	0.37399900
C	3.89930500	4.33733700	0.04016400
C	1.73022000	5.39357700	-0.10239000
H	0.01599800	4.11886900	-0.00095000
C	-0.12754500	1.63058100	2.85948800
C	-2.30960600	1.64331800	1.74768500
C	-2.46279500	1.58486400	-0.66450300
O	-0.49004600	1.57724400	-2.01480100
C	3.14336200	5.48046800	-0.08930900
H	4.98463400	4.38687900	0.05674300
H	1.13865300	6.29886600	-0.20255000
C	-0.75655500	1.63388000	4.08553000
H	0.95507500	1.62357100	2.80102900
C	-2.92869700	1.64891200	3.02458600
C	-3.08228700	1.61814100	0.55793000
I	-3.63632600	1.43983400	-2.44687800
H	0.29676200	0.99035100	-1.96928400
H	3.62614500	6.44882300	-0.17955400
C	-2.16980200	1.64346700	4.17301600
H	-0.16324400	1.62791400	4.99523100
H	-4.01366800	1.64003900	3.07541700
H	-4.16360600	1.59693700	0.63380400
H	-2.65092800	1.63969400	5.14628200
C	0.14398700	-1.23734800	-3.00412000
H	-0.92216400	-0.99230700	-2.92464800
H	0.22891800	-2.32497700	-3.07870500
C	0.75256500	-0.58797200	-4.23320000
H	1.82101000	-0.81744300	-4.29810400
H	0.26052100	-0.96327200	-5.13638500
H	0.63225100	0.49949800	-4.21035700

TS9$_{Int1-B}$

O	-0.76814700	0.21794500	-0.49837000
B	-0.60511400	-0.34946800	0.79741500
C	0.60205700	0.07608500	1.76984300
H	1.04005200	1.06461500	1.67026200
C	1.01108300	-0.71625600	2.78867200
H	0.50062900	-1.66654000	2.93293600
C	2.11673400	-0.46791400	3.70864600
C	2.31736900	-1.35402500	4.78279800
C	3.01691800	0.60329400	3.54497900
C	3.37356400	-1.17283100	5.67300600
H	1.63739900	-2.19185700	4.90754500
C	4.07561400	0.77945300	4.42899500
H	2.89632400	1.28451600	2.70909300
C	4.25809600	-0.10660700	5.49750300
H	3.51155600	-1.86609900	6.49739900
H	4.76598600	1.60545600	4.28556600
H	5.08739200	0.03296400	6.18455000
C	-1.69663800	0.22975100	1.91105700
H	-1.89238200	-0.45172900	2.73880800
C	-1.74933500	1.67536200	2.40616800
F	-1.48652100	2.55740400	1.41704100
F	-2.96945300	1.98391900	2.89766800
F	-0.86218800	1.88966300	3.39015900
N	-3.22555700	0.12978300	1.09055100
N	-3.66910500	-0.14122600	0.10553000
O	-0.84236600	-1.80697900	0.78241600
C	-0.71557800	-2.60621900	-0.28561700
C	0.44586300	-2.68198400	-1.05963800
C	-1.79775000	-3.48549400	-0.60038500
C	0.50136800	-3.55036000	-2.20093400
C	1.68295500	-1.95744400	-0.64528600
C	-1.77126500	-4.31983800	-1.68653300
I	-3.48724000	-3.56855300	0.71551400
C	-0.63475000	-4.35402100	-2.53394300
C	1.65695000	-3.65664400	-3.02485300
C	2.47113800	-2.44365600	0.44246100
C	2.12196100	-0.86420100	-1.38134500
H	-2.61216000	-4.96759400	-1.90886500
C	-0.59279700	-5.20041000	-3.67326400
C	1.66996300	-4.48795000	-4.12352000
H	2.53869400	-3.07724900	-2.77762700
C	2.09451100	-3.57831400	1.21053100
C	3.69013500	-1.77451200	0.78050200
C	3.34286500	-0.21903700	-1.04400900
O	1.43178700	-0.38585000	-2.44553500
C	0.53512700	-5.26412700	-4.45948900
H	-1.46934500	-5.79821100	-3.90760200
H	2.56281600	-4.54915300	-4.73873600
C	2.88103500	-4.01902600	2.25289700
H	1.17698400	-4.09983000	0.96362100

C	4.47749500	-2.24943100	1.86207200
C	4.10043400	-0.65316300	0.01390700
I	4.04333500	1.40408900	-2.24322000
H	0.59256000	-0.87224700	-2.51985400
H	0.56070900	-5.91160800	-5.33062200
C	4.08361700	-3.35109900	2.58633900
H	2.57480900	-4.88908200	2.82638300
H	5.39312400	-1.72007700	2.10918100
H	5.03035200	-0.15567800	0.26681900
H	4.68708800	-3.70490300	3.41620500
B	-0.39410900	1.30757200	-1.21475200
O	-1.05242700	1.51091900	-2.41892600
B	-2.35443800	1.81657000	-2.68961000
O	-2.76459600	1.61814100	-3.98320600
H	-3.66452400	1.93726400	-4.13253300
C	-3.27743900	2.38107000	-1.56383900
H	-2.79314400	2.74484400	-0.65904600
C	-4.62476300	2.37969700	-1.60636200
H	-5.12219800	2.00110600	-2.50132200
C	0.68965800	2.33326100	-0.78079800
H	1.42376500	2.05524800	-0.03048200
C	0.73796600	3.58008900	-1.28869400
H	0.00837200	3.85539600	-2.05015100
C	1.68624300	4.64498200	-0.94326300
C	1.59061500	5.88256800	-1.60435200
C	2.70268500	4.48127700	0.01777100
C	2.47647500	6.92123500	-1.32042200
H	0.81162600	6.02234100	-2.34935600
C	3.58728700	5.51711500	0.30162300
H	2.79970100	3.53740000	0.54380000
C	3.47927200	6.74227000	-0.36576800
H	2.38438500	7.86844900	-1.84392600
H	4.36537300	5.37137700	1.04551500
H	4.17123600	7.54868500	-0.14144800
C	-5.55006100	2.79882100	-0.54695300
C	-6.93389300	2.75144400	-0.79250000
C	-5.11121600	3.22418400	0.72171400
C	-7.85125100	3.12129200	0.19007400
H	-7.28599200	2.41853800	-1.76539900
C	-6.02693600	3.58856900	1.70371200
H	-4.05202600	3.26286400	0.94562600
C	-7.40085800	3.54094400	1.44310100
H	-8.91575300	3.07901500	-0.02110700
H	-5.66902500	3.90784100	2.67825800
H	-8.11217600	3.82580000	2.21260100

TS9$_1$

C	-2.81218500	-0.51171900	-0.36736400
H	-3.08016000	-1.48646200	-0.77146700
C	-3.63031400	0.54844900	-0.55467500
H	-3.33897200	1.49786800	-0.10537500
C	-4.85855700	0.59832900	-1.34680500
C	-5.47046900	1.84672200	-1.56786200
C	-5.46315300	-0.55043200	-1.89523400
C	-6.64022400	1.94931200	-2.31665700
H	-5.01406000	2.73817500	-1.14647500
C	-6.63177500	-0.44679100	-2.64140300
H	-5.02090400	-1.52670100	-1.72629600
C	-7.22451500	0.80222100	-2.85737700
H	-7.09637300	2.92142500	-2.47745400
H	-7.08741500	-1.34156400	-3.05472300
H	-8.13766900	0.87752200	-3.43986100
C	-2.24770700	-0.70605800	1.89165100
H	-2.85812500	0.12393300	2.24920900
C	-2.91239400	-2.03669000	2.23755200
F	-2.13206200	-3.08584300	1.93089600
F	-3.18998300	-2.11413500	3.55733600
F	-4.07478400	-2.17063000	1.57480600
N	-1.00015800	-0.63256800	3.09119800
N	0.05313300	-0.29812100	3.23053200
B	-1.43693200	-0.41973300	0.47495700
O	-0.87252200	0.91280700	0.56002100
O	-0.48612700	-1.46165700	0.16641200
B	0.43429800	1.15873000	0.27076100
B	0.82086800	-1.19027900	-0.09127200
O	1.28886000	0.11883200	-0.07252900
C	0.98597700	2.60889000	0.33283600
C	1.81022000	-2.34681300	-0.39919900
H	0.29748500	3.41274500	0.59440200
C	2.27703900	2.90267100	0.07823100
C	3.12171300	-2.13556300	-0.63041900
H	1.42170000	-3.36523900	-0.41851000
H	2.94127500	2.07714100	-0.17732700
C	2.92890000	4.21731600	0.10079100
H	3.47900300	-1.10607800	-0.60619800
C	4.16322200	-3.12899700	-0.91727100
C	4.30594300	4.29326000	-0.17493900
C	2.24059600	5.41217400	0.38583200
C	5.47677300	-2.68284200	-1.14898900
C	3.91209600	-4.51352500	-0.96959300

C	4.97605100	5.51535100	-0.16569300
H	4.84916000	3.37863700	-0.39754600
C	2.90898900	6.63230800	0.39484700
H	1.17693900	5.38509300	0.59935400
C	6.50503700	-3.58238400	-1.42496400
H	5.68462900	-1.61678300	-1.11081900
C	4.93822100	-5.41181000	-1.24479500
H	2.90906500	-4.88783700	-0.79225300
C	4.27940600	6.69051500	0.11965700
H	6.03983900	5.55024400	-0.38115300
H	2.36149400	7.54374900	0.61645800
C	6.23928100	-4.95157900	-1.47401700
H	7.51183300	-3.21527800	-1.60079900
H	4.72561000	-6.47626400	-1.28058200
H	4.79719200	7.64488200	0.12756700
H	7.03720700	-5.65626200	-1.68790000

Int9(*S*)

C	-5.86621400	2.74031900	0.77077800
C	-5.81295600	1.43056400	0.35429300
C	-4.56551800	0.80314600	0.09046400
C	-3.35255400	1.55229900	0.22433800
C	-3.44461300	2.89350100	0.68723400
C	-4.66720200	3.46905500	0.95381500
H	-5.43419500	-1.12522200	-0.36974300
H	-6.82241600	3.21168000	0.97598700
H	-6.72137800	0.84750700	0.23356500
C	-4.50951100	-0.56753400	-0.27205500
C	-2.09875500	0.91485800	-0.07502800
H	-2.53865100	3.46370900	0.84687100
H	-4.71048500	4.49222600	1.31454500
C	-2.09030700	-0.44531200	-0.33456300
C	-3.29649700	-1.18188100	-0.44800500
C	-0.82553900	1.68377900	-0.08222400
C	-0.68374700	2.88006000	-0.86611000
C	0.25192500	1.24991300	0.67621400
C	-1.65061200	3.29123400	-1.82417900
C	0.49333000	3.67968000	-0.71278600
C	1.40580900	2.05995400	0.83453400
C	-1.47723100	4.44613400	-2.55428800
H	-2.52656700	2.67753100	-1.99165500
C	0.63460700	4.87651600	-1.46587200
C	1.51384000	3.25938400	0.17754500
C	-0.33325500	5.25774900	-2.36561200
H	-2.22483600	4.73435700	-3.28700800
H	1.53020400	5.47451700	-1.32379300
H	2.39062800	3.88390500	0.30711900
H	-0.21635500	6.17011900	-2.94222100
O	0.26109700	0.00908400	1.28037400
O	-0.91742500	-1.15212400	-0.47070100
B	0.05063000	-1.10750500	0.50000100
I	2.97962400	1.43264000	2.13182400
I	-3.20999900	-3.27399500	-0.86817500
C	1.00478600	-2.36762700	0.61691200
H	0.57928600	-3.17447300	0.01340900
C	2.32140300	-1.90711000	0.04104400
H	2.97117600	-1.36778600	0.72319200
C	2.64232200	-2.03928900	-1.25504000
H	1.95863400	-2.58220400	-1.90715500
C	3.84904100	-1.51712700	-1.90775800
C	4.11988800	-1.89525400	-3.23452700
C	4.74638100	-0.64059200	-1.26743100
C	5.25381700	-1.42776900	-3.89814600
H	3.43148000	-2.56480100	-3.74351700
C	5.87796800	-0.17500600	-1.93042900
H	4.55032800	-0.31129300	-0.25216800
C	6.13932400	-0.56665000	-3.24795700
H	5.44409900	-1.73454900	-4.92257500
H	6.55702900	0.50260200	-1.42079600
H	7.02202700	-0.19839500	-3.76229800
C	1.13676600	-2.90040900	2.02079200
F	1.71312400	-2.01524500	2.86678900
F	1.88429000	-4.02830500	2.07257700
F	-0.07884000	-3.20815000	2.54082700

Int9(*R*)

C	-5.10137900	0.38535100	-3.64576700
C	-4.75441200	1.47461400	-2.88101100
C	-3.79548400	1.35392900	-1.83914400
C	-3.21126600	0.07727300	-1.55750400
C	-3.57094600	-1.01890400	-2.38853200
C	-4.48842300	-0.86702600	-3.40448300
H	-3.84233100	3.45342100	-1.31362800
H	-5.83028100	0.48618100	-4.44398200
H	-5.19525300	2.44954100	-3.06803500
C	-3.38943500	2.49249100	-1.09619000
C	-2.26494800	-0.03823800	-0.48095800
H	-3.10421600	-1.98228700	-2.22840200
H	-4.74002000	-1.71752800	-4.03078700
C	-1.82808700	1.11731400	0.14587300

C	-2.40415000	2.37862200	-0.14935000
C	-1.74191800	-1.36235100	-0.04950900
C	-2.63242500	-2.44023900	0.28615800
C	-0.37522600	-1.57488500	0.04585900
C	-4.03787500	-2.26624000	0.41374600
C	-2.09051500	-3.74014500	0.54258500
C	0.15092400	-2.86724500	0.29978100
C	-4.85712500	-3.32660000	0.73211800
H	-4.46641200	-1.28216400	0.27402400
C	-2.96324800	-4.81917400	0.84877600
C	-0.68553400	-3.93267100	0.51354200
C	-4.32135900	-4.62037400	0.93610900
H	-5.92616000	-3.16594800	0.83347600
H	-2.53120900	-5.79980000	1.02628400
H	-0.28505000	-4.92370000	0.69541900
H	-4.98177700	-5.44766300	1.17692100
O	0.53528400	-0.54746200	-0.07615400
O	-0.79469600	1.10196300	1.05796700
B	0.42376500	0.58510300	0.69033900
I	2.26552700	-3.15779500	0.35059000
I	-1.71071800	4.11285500	0.88687200
C	1.75066700	1.33443500	1.15269000
C	2.93600600	0.89995900	0.33563900
H	3.26018900	-0.12581400	0.48344500
C	3.53765600	1.68396100	-0.56870800
H	3.19333000	2.71200300	-0.67962800
C	4.64924400	1.29578900	-1.44767800
C	5.32159800	2.29295000	-2.17554700
C	5.07065400	-0.03909500	-1.60125700
C	6.38965000	1.97526800	-3.01423800
H	5.00122100	3.32660900	-2.07441900
C	6.13709300	-0.35560400	-2.43808400
H	4.55123500	-0.83367100	-1.07468000
C	6.80422300	0.64901600	-3.14724300
H	6.89629200	2.76318500	-3.56414900
H	6.44579800	-1.39163500	-2.54500400
H	7.63370700	0.39729900	-3.80139900
H	1.58129400	2.41184000	1.04371000
C	1.98360000	1.09676300	2.63144400
F	3.10855300	1.69549500	3.08255500
F	2.10779800	-0.22759400	2.91098900
F	0.95985300	1.56062700	3.38472800

TS10(*S*)

C	-3.30485000	0.84589300	-0.38119900
C	-4.01216800	1.67408200	0.45304900
C	-1.91090000	0.61774200	-0.19692400
C	-3.35946200	2.32212700	1.53421700
C	-1.21805800	1.38029800	0.73950700
C	-1.94170400	2.18555400	1.67920900
O	-1.26260700	-0.27243500	-0.97195300
B	-0.63004200	-1.44661100	-0.38197800
C	0.24047600	-2.11063700	-1.61128800
O	-1.50746000	-2.31032000	0.37910000
C	-2.66383300	-2.92322300	-0.19770600
H	-2.47904200	-3.99814200	-0.28915500
H	-2.83265500	-2.52581700	-1.20188900
H	-5.07309100	1.84090000	0.30201400
C	0.26958700	1.39859800	0.70943900
C	0.97572900	2.63872100	0.53284700
C	1.00182700	0.22955700	0.81939000
C	2.40193700	2.65417400	0.64791600
C	2.41077800	0.26004800	0.97851600
C	3.09569800	1.44607700	0.91069900
H	4.17236800	1.47233000	1.03108200
O	0.35263000	-0.99004500	0.80113200
C	-4.08646800	3.12150100	2.45642400
H	-5.16035400	3.21883200	2.32335100
C	-3.44597300	3.75134200	3.49898200
H	-4.00925300	4.35658200	4.20269600
C	-2.04785100	3.59973200	3.66034400
H	-1.54853700	4.08267900	4.49509100
C	-1.31460000	2.84209800	2.77380800
H	-0.24682400	2.72767800	2.91698400
C	0.31832200	3.85645000	0.20772400
H	-0.75610400	3.85934100	0.07564300
C	1.03195600	5.02220600	0.03575900
H	0.50903400	5.93745800	-0.22500800
C	2.43831800	5.03968200	0.18986400
H	2.98534900	5.96811100	0.05771000
C	3.10853000	3.87543100	0.48562400
H	4.19005100	3.86400800	0.58170800
C	-3.87172200	-2.66791400	0.68664400
H	-3.71375800	-3.08553800	1.68766500
H	-4.06401700	-1.59650600	0.78580300
H	-4.76182800	-3.14175100	0.25899600
I	-4.28763100	-0.01541200	-2.07561700
I	3.49339300	-1.53176500	1.39257800
H	-1.20282300	-2.38323200	2.05966900
O	-0.48832900	-2.13967800	2.71186800

H	0.08073800	-1.47135400	1.81430600
C	0.25041800	-3.35818000	3.03462500
H	1.11067800	-3.02936400	3.61995400
H	0.60758200	-3.80625800	2.10374600
C	-0.63264700	-4.30904900	3.81410100
H	-1.49296400	-4.62641800	3.21483700
H	-0.06074100	-5.20292300	4.08322400
H	-0.99774700	-3.84010100	4.73259300
H	-0.43270300	-2.11102300	-2.47655200
C	0.63778200	-3.54596400	-1.40643800
F	1.48331000	-3.99956900	-2.37318400
F	-0.42370400	-4.39043100	-1.41791400
F	1.28431100	-3.75904300	-0.22669300
C	1.44556000	-1.25762200	-1.89682200
H	2.37867600	-1.58779800	-1.44957700
C	1.39830400	-0.07544500	-2.52725000
H	0.44069600	0.27149700	-2.90971800
C	2.50509600	0.88125400	-2.63999100
C	2.21219300	2.21797800	-2.96478100
C	3.84688000	0.54009700	-2.38510000
C	3.21540800	3.18408900	-3.01388400
H	1.18013000	2.49907300	-3.15602300
C	4.84944100	1.50522500	-2.43466300
H	4.10565100	-0.48667300	-2.14659400
C	4.54002500	2.83330600	-2.74577300
H	2.96074300	4.21301600	-3.25031900
H	5.87830100	1.22058700	-2.23230000
H	5.32397800	3.58406700	-2.78122800

Int10(*S*)

C	-3.70190100	0.36979200	-0.21081800
C	-4.34930200	1.57856400	-0.14512800
C	-2.29887500	0.30785500	-0.40488000
C	-3.61915600	2.78924500	-0.26868200
C	-1.54733700	1.46808700	-0.52376000
C	-2.20112900	2.74005900	-0.46357000
O	-1.69704900	-0.91232400	-0.53125800
B	-0.85678600	-1.43579600	0.43295600
C	-0.06817700	-2.74005300	-0.05826700
O	-0.78600900	-0.78911300	1.61358700
C	0.12188400	-1.05102200	2.70063900
H	1.08131800	-0.58491500	2.45830200
H	0.26948300	-2.12585700	2.81027200
H	-5.42202600	1.62431100	0.00736100
C	-0.06901500	1.37769800	-0.70900300
C	0.50213600	1.03823700	-1.97238800
C	0.75638400	1.64287600	0.37844800
C	1.92649400	1.00884700	-2.10984900
C	2.16794100	1.59002200	0.22700600
C	2.74063800	1.29404700	-0.98293300
H	3.81815600	1.24892500	-1.08844200
O	0.26295300	1.95971100	1.59940100
C	-4.27089600	4.05015800	-0.20419600
H	-5.34669500	4.07187200	-0.05538500
C	-3.55301200	5.21766700	-0.32724200
H	-4.05821300	6.17724800	-0.27620800
C	-2.15137000	5.17075800	-0.52372100
H	-1.59322600	6.09668500	-0.62447300
C	-1.49049600	3.96424200	-0.58960000
H	-0.41804700	3.93602600	-0.74620400
C	-0.29273400	0.72475800	-3.10790100
H	-1.37305200	0.74880500	-3.01783600
C	0.29486100	0.39311600	-4.30921800
H	-0.32910300	0.15143900	-5.16462000
C	1.70447700	0.36688400	-4.44313900
H	2.15170200	0.10646800	-5.39758500
C	2.50203500	0.67442100	-3.36384200
H	3.58398000	0.65597300	-3.44775300
C	-0.45965700	-0.45710700	3.96688300
H	-0.58199500	0.62534300	3.86983800
H	-1.43307100	-0.90327200	4.19338300
H	0.21453000	-0.65092500	4.80727800
I	-4.79839500	-1.45158500	0.00045800
I	3.40559900	1.94689500	1.93174800
H	-0.68355400	1.73897900	1.61635600
H	-0.33372200	-2.85000500	-1.11477500
C	-0.54570800	-4.01491000	0.59642100
F	-0.06434500	-5.11920300	-0.01959900
F	-1.89924800	-4.10457800	0.56818100
F	-0.17927200	-4.12119100	1.90124800
C	1.42057500	-2.54308900	0.05612100
H	1.84770700	-2.61962600	1.05185300
C	2.19619800	-2.22909300	-0.99166300
H	1.74270200	-2.18923900	-1.98099100
C	3.63074600	-1.92091200	-0.94720600
C	4.36397400	-1.91930000	-2.14646400
C	4.29972000	-1.58264700	0.24315800
C	5.72161700	-1.60137300	-2.15799700
H	3.85568700	-2.16340100	-3.07471700
C	5.65520800	-1.26492600	0.23243600

H	3.74988800	-1.53620200	1.17697500
C	6.37363700	-1.27213000	-0.96751900
H	6.26912200	-1.60653600	-3.09611500
H	6.14966600	-0.99434400	1.16076600
H	7.42896900	-1.01627000	-0.97402600

TS11(*S*)

C	3.21070300	0.40532800	-1.15856200
C	3.68895000	1.43962200	-1.92571700
C	2.04019200	0.54791300	-0.35624000
C	3.03207600	2.69379700	-1.93248800
C	1.38031100	1.77463500	-0.34332000
C	1.86783400	2.86993300	-1.12223200
O	1.57553200	-0.48547300	0.40955500
B	0.35347200	-1.46813000	-0.05730700
O	-0.09674500	-1.07077600	-1.32834900
O	1.04200600	-2.76699000	0.03798300
C	0.30792800	-3.95196400	-0.26446000
H	-0.24535500	-4.28235600	0.62354900
H	-0.42621900	-3.73785600	-1.04894300
C	0.53871000	-1.46337900	-2.53150700
H	0.02955800	-2.35090600	-2.93914200
H	1.58036200	-1.74999500	-2.35595500
H	4.57627700	1.30950100	-2.53513600
C	0.17542700	1.95479200	0.51119900
C	0.29873700	2.00524400	1.93024100
C	-1.07738900	2.05796800	-0.08794100
C	-0.88019300	2.09173100	2.73655500
C	-2.23519200	2.16776200	0.73329600
C	-2.14562900	2.16474100	2.10307700
H	-3.03905800	2.22180800	2.71463700
O	-1.13509900	2.03850000	-1.43892200
H	-2.06112900	2.03056800	-1.73373200
C	3.51647400	3.77628000	-2.71608400
H	4.40002400	3.61981500	-3.32862000
C	2.88017500	4.99549900	-2.69359400
H	3.25416900	5.81938700	-3.29384400
C	1.73584700	5.18040400	-1.87895600
H	1.24418700	6.14845800	-1.85791900
C	1.24251300	4.14741900	-1.11424000
H	0.37068700	4.30257200	-0.48970200
C	1.55973000	1.95980000	2.58614000
H	2.46206400	1.91548400	1.98722500
C	1.64203300	1.97777500	3.95961000
H	2.61515600	1.94786500	4.44121100
C	0.47059900	2.03523400	4.75506700
H	0.55263800	2.03846200	5.83769800
C	-0.76435500	2.09323900	4.15275700
H	-1.67218700	2.14485400	4.74735900
C	1.27452900	-5.03038800	-0.72171800
H	2.01187500	-5.24817200	0.05937700
H	0.73593100	-5.95667800	-0.94856000
H	1.81192600	-4.71092300	-1.62024300
C	0.46633400	-0.32834600	-3.54108100
H	-0.57473600	-0.03991600	-3.71859600
H	1.00243800	0.55159800	-3.17708300
H	0.90709200	-0.63737800	-4.49586600
I	4.29890800	-1.43757500	-1.23365000
I	-4.16976000	2.29355100	-0.18683800
H	2.09587100	-2.71699900	1.27335300
O	2.53982400	-2.05203700	1.89644000
H	2.29085400	-1.14792200	1.25228100
C	3.89873100	-2.32844600	2.33171000
H	3.81768200	-3.07237300	3.12802800
H	4.46550300	-2.75063600	1.49925800
C	4.50845500	-1.03709300	2.83086700
H	5.50493000	-1.23594500	3.23616300
H	4.60875900	-0.31165400	2.01733500
H	3.89110900	-0.59941600	3.62084900
C	-0.91301300	-1.25614500	1.00095900
H	-1.11224000	-0.18643900	0.97512300
C	-0.66004300	-1.57496100	2.44516000
F	0.34490800	-0.85649800	2.99181300
F	-0.35088000	-2.89022400	2.65492200
F	-1.75396800	-1.33416000	3.22955100
C	-2.14770500	-1.98076600	0.54042700
H	-2.37788800	-2.92154300	1.03637600
C	-2.93862900	-1.53743500	-0.44877700
H	-2.67037400	-0.60155300	-0.93127400
C	-4.14125400	-2.18865500	-0.97882400
C	-4.87304000	-1.53510000	-1.98798500
C	-4.61147800	-3.43947400	-0.53175800
C	-6.02863500	-2.09979500	-2.52667600
H	-4.52441700	-0.57006900	-2.34582500
C	-5.76533800	-4.00343200	-1.06894400
H	-4.06838800	-3.97580100	0.24033800
C	-6.48272300	-3.33812100	-2.06923700
H	-6.57405800	-1.57221300	-3.30433100
H	-6.10790900	-4.96925100	-0.70762000
H	-7.38203100	-3.78202100	-2.48592700

4(*S*)

O	-1.84721300	1.84359300	-0.24820200
O	-2.50809500	-0.00287400	1.03618100
B	-1.80308900	0.50593400	-0.00748400
C	-0.88758700	-0.50429600	-0.85360000
H	-0.76638600	-0.12625000	-1.87373800
C	0.45716900	-0.57314100	-0.17619800
H	0.46339200	-1.04258800	0.80477500
C	1.58204100	-0.05453100	-0.69171300
H	1.53727700	0.38609600	-1.68693700
C	2.90701900	-0.02381800	-0.05898300
C	4.00651700	0.41848000	-0.81525700
C	3.13257000	-0.41544800	1.27429200
C	5.28805800	0.45836500	-0.26789700
H	3.84805800	0.72937700	-1.84442500
C	4.41203400	-0.37663700	1.82015500
H	2.30092600	-0.74420500	1.88925500
C	5.49715000	0.05887700	1.05267700
H	6.12219800	0.80172900	-0.87270600
H	4.56466000	-0.68245600	2.85107700
H	6.49339300	0.09006900	1.48324500
C	-1.50761700	-1.87360000	-0.99742800
F	-1.58707400	-2.55021000	0.17128200
F	-0.80102900	-2.66484900	-1.84740000
F	-2.76657700	-1.80009100	-1.50067800
C	-1.10935500	2.49474600	-1.29267600
H	-0.03723700	2.33667100	-1.13230000
H	-1.37498500	2.05663200	-2.26185600
C	-3.29725700	0.85430900	1.87947500
H	-2.65645400	1.63384400	2.30674400
H	-4.06379600	1.35233100	1.27498600
C	-1.44374500	3.97260800	-1.26647700
H	-0.89286400	4.49598700	-2.05440600
H	-2.51453800	4.13024800	-1.42941200
H	-1.17123800	4.41345500	-0.30251500
C	-3.92689600	0.00795800	2.96720300
H	-4.53764800	0.63416400	3.62558800
H	-4.56823600	-0.76590700	2.53366600
H	-3.15709900	-0.48151000	3.57227300

TS12(*S*)

O	-0.35090000	2.06715300	0.34162800
B	-1.10733200	0.91235400	-0.00910100
O	-1.28578200	0.07489100	1.22405300
C	-0.33494800	0.15175900	2.29478100
H	-0.54881300	1.02966900	2.91691900
H	0.66323200	0.28672500	1.87251400
C	-0.41119600	-1.12486200	3.11108000
H	-1.41039900	-1.26042300	3.54029400
H	-0.18644400	-1.99630000	2.48760200
H	0.30944400	-1.09173300	3.93490800
O	-2.53307700	1.30297200	-0.43066400
H	-2.75078200	0.97279200	-1.31315000
H	-2.64736400	0.03453900	1.55492200
O	-3.68588800	0.23446700	1.37010600
H	-3.36594200	0.81833100	0.46437200
C	-4.35977400	-0.99347400	0.95846900
H	-4.39260300	-1.63833000	1.83981800
H	-3.75790200	-1.48388400	0.18831300
C	-5.74753800	-0.65972500	0.45792600
H	-6.26798700	-1.58123500	0.17916800
H	-6.33013200	-0.15249100	1.23244200
H	-5.70246100	-0.01461600	-0.42579200
C	-0.38424200	0.00637200	-1.22109300
H	-0.22350900	0.65914000	-2.08737800
C	-1.21449100	-1.13565100	-1.73169100
F	-2.40085900	-0.71277400	-2.28326700
F	-1.55420500	-2.02961500	-0.76542700
F	-0.60594200	-1.85889900	-2.70797900
C	0.93120800	-0.50291600	-0.71848200
H	0.88658000	-1.40450200	-0.11102000
C	2.10407000	0.12245200	-0.89859500
H	2.12324600	1.01874100	-1.51745600
C	3.39990600	-0.26536600	-0.32801600
C	4.54958400	0.45447100	-0.69979600
C	3.55318100	-1.32381800	0.58878800
C	5.80427700	0.12874100	-0.18646600
H	4.44989200	1.27823400	-1.40179100
C	4.80580500	-1.65058500	1.09956300
H	2.68472000	-1.89110800	0.90801000
C	5.93979900	-0.92740200	0.71562500
H	6.67587700	0.70106600	-0.49087100
H	4.89904800	-2.47079900	1.80573100
H	6.91485900	-1.18338000	1.11897200
C	-0.17888700	3.11114200	-0.59276100
H	0.23869700	2.73330500	-1.53984000
H	-1.14846000	3.57069500	-0.83830700
C	0.76227000	4.14989700	-0.00595700
H	1.73761200	3.70145500	0.21113600

H	0.90962300	4.98092100	-0.70413900
H	0.35672600	4.55092500	0.92894000

4-OH(*S*)

O	1.10449900	-2.05327700	-0.15341500
O	3.11321900	-0.81410100	-0.10844100
B	1.76680400	-0.86456600	-0.24155000
C	0.95415700	0.49319200	-0.54935000
H	0.91852800	0.59124600	-1.64272100
C	-0.44734800	0.40245800	-0.00805600
H	-0.55592500	0.59239600	1.05780300
C	-1.50734400	0.03734800	-0.74627800
H	-1.35799700	-0.12724300	-1.81336100
C	-2.88696000	-0.15508700	-0.27744100
C	-3.89183300	-0.41121200	-1.22628400
C	-3.25369900	-0.09744700	1.08021200
C	-5.21856000	-0.59341200	-0.83844200
H	-3.62338100	-0.46363400	-2.27803800
C	-4.57817700	-0.27803300	1.46702200
H	-2.49820700	0.08294900	1.83817200
C	-5.56815700	-0.52597500	0.51067700
H	-5.97795000	-0.78810300	-1.58994100
H	-4.84060600	-0.22970700	2.51979000
H	-6.59996600	-0.66863900	0.81704200
C	1.65211400	1.72911900	-0.03569200
F	1.86455700	1.66950300	1.30582100
F	0.92295700	2.85613700	-0.25197700
F	2.85188300	1.93209100	-0.62034800
C	3.87496600	-2.00814700	0.15087000
H	3.48071600	-2.50431500	1.04465600
H	3.76000000	-2.70011000	-0.69123500
C	5.32661600	-1.61740700	0.34012500
H	5.93238800	-2.50702700	0.54048500
H	5.71722000	-1.12816400	-0.55773400
H	5.43504800	-0.92816300	1.18349900
H	0.14514300	-1.93158000	-0.23145700

TS13(*S*)

O	-3.45237700	1.01247900	-0.53903900
B	-2.13577200	0.71452600	-0.07487200
O	-1.19109800	1.73579000	-0.63204900
C	-1.24122300	2.10166400	-2.00864300
H	-2.10369900	2.75440400	-2.18762900
H	-1.38389000	1.19799200	-2.61251900
C	0.05537800	2.79376500	-2.38868800
H	0.20138600	3.70312600	-1.79504500
H	0.91154700	2.13204400	-2.22290000
H	0.03741600	3.07983000	-3.44523500
O	-1.96661800	0.92017900	1.43039300
H	-2.00707700	0.09114600	1.92344600
H	-0.52374600	2.40890900	0.34590500
O	-0.20763600	2.55308600	1.37862500
H	-0.93671500	1.79824100	1.67189700
C	1.15610800	2.05062200	1.53176000
H	1.81522200	2.87119000	1.23998200
H	1.29743300	1.22142900	0.83627300
C	1.39420600	1.62549200	2.96357600
H	2.42646300	1.27958000	3.07156000
H	1.23313800	2.45870700	3.65398300
H	0.72879700	0.80291100	3.24653700
H	-4.02028200	0.23521600	-0.50441800
C	-1.58801300	-0.81963400	-0.49710200
H	-1.69398100	-0.90096100	-1.58501300
C	-2.43217800	-1.91871300	0.07724100
F	-3.74184200	-1.82681200	-0.29313600
F	-2.43254100	-1.90745400	1.45049200
F	-2.03606300	-3.16785900	-0.27266100
C	-0.15536700	-1.01145000	-0.11162000
H	0.03355500	-1.20106700	0.94357100
C	0.87920300	-0.87963700	-0.95588300
H	0.66554800	-0.69057000	-2.00734800
C	2.30609700	-0.92285200	-0.61179400
C	3.25632500	-0.71343600	-1.62722600
C	2.77860700	-1.14138500	0.69769100
C	4.62297700	-0.71874800	-1.35084600
H	2.91112300	-0.54233400	-2.64349700
C	4.14272000	-1.14651100	0.97368900
H	2.07354000	-1.30804100	1.50569000
C	5.07411400	-0.93417400	-0.04801300
H	5.33521800	-0.55325200	-2.15398400
H	4.48254900	-1.31697000	1.99125700
H	6.13744300	-0.93729700	0.17129300

4-2OH(*S*)

O	-2.60870400	1.47439600	1.46779900
O	-2.12006300	2.69814300	-0.48194200
B	-2.16244200	1.50678300	0.18263300
C	-1.62570000	0.14825100	-0.47655000
H	-1.61736500	0.25447400	-1.56704300
C	-0.22076800	-0.07935600	0.02131300

H	-0.13606500	-0.41249500	1.05270800
C	0.87393400	0.17115000	-0.71157200
H	0.73976100	0.50175400	-1.74132600
C	2.27261600	0.04586100	-0.28185100
C	3.29043200	0.29765700	-1.21851300
C	2.64893700	-0.31549400	1.02579800
C	4.63552700	0.18924600	-0.86826800
H	3.01663800	0.57933200	-2.23178600
C	3.99147000	-0.42495600	1.37482500
H	1.88773000	-0.50903900	1.77471700
C	4.99254400	-0.17359300	0.43083500
H	5.40350300	0.38805500	-1.60996900
H	4.26039100	-0.70518700	2.38910300
H	6.03883600	-0.25866200	0.70816800
C	-2.52011900	-1.03296200	-0.18671600
F	-2.54154500	-1.37130200	1.12312300
F	-2.13609200	-2.14533200	-0.86356000
F	-3.80382700	-0.78177400	-0.55140700
H	-2.86459700	2.35498100	1.78292300
H	-1.78656000	2.62341100	-1.38673000
